# Macrophage Signaling Pathways in Health and Disease: From Bench to Bedside Applications

**DOI:** 10.1002/mco2.70256

**Published:** 2025-06-16

**Authors:** Yongquan Chi, Haipeng Jiang, Yiyuan Yin, Xinyu Zhou, Yiyouyou Shao, Yongsheng Li, Jianhua Rao

**Affiliations:** ^1^ Hepatobiliary Center Key Laboratory of Liver Transplantation Chinese Academy of Medical Sciences NHC Key Laboratory of Hepatobiliary Cancers The First Affiliated Hospital of Nanjing Medical University Nanjing Jiangsu China; ^2^ Nanjing Medical University Nanjing Jiangsu China; ^3^ Department of Medical Oncology Chongqing University Cancer Hospital Chongqing China

**Keywords:** injury and repair, macrophage, micro‐environment, polarization, signaling pathways

## Abstract

Macrophages exhibit remarkable functional plasticity by dynamically polarizing into proinflammatory or antiinflammatory subsets in response to microenvironmental cues. This duality underpins their pivotal roles in immune defense, tissue homeostasis, and disease progression; however, the molecular mechanisms governing their polarization and crosstalk across various pathologies remain incompletely defined. This review systematically delineates macrophage biology, emphasizing the interplay between subset‐specific signaling networks and their context‐dependent activation in both health and disease. The heterogeneity of macrophages is characterized by detailing the distinctions between tissue‐resident and monocyte‐derived origins, as well as their polarization states. Core pathways regulating phagocytosis, tissue repair, immune modulation, and neuroprotection are dissected, along with their dysregulation in autoimmune disorders, neurodegeneration, cancers, and cardiovascular diseases. Notably, microenvironmental factors such as damage‐associated molecular patterns, pathogen‐associated molecular patterns, and metabolic intermediates dynamically reshape macrophage phenotypes through NLR Family Pyrin Domain Containing 3 (NLRP3) inflammasome activation or signal transducer and activator of transcription (STAT)‐mediated transcriptional control. Preclinical and clinical evidence underscores potential therapeutic targets and emerging strategies. The significance of this review lies in its integrative analysis of signaling crosstalk, paradoxical pathway roles, and translational implications for precision therapies. These insights into macrophage functions and signaling pathways provide a robust foundation for future disease intervention and personalized medicine.

## Graphical abstract

Macrophage functions in phagocytosis, tissue repair, immune regulation, and neuronal network protection. And graphic abract image in the attachment.

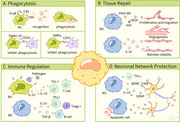

## 1 Introduction

Since Élie Metchnikoff's discovery of macrophages at the end of the 19th century [1], these cells have been recognized as pivotal in immune regulation, tissue homeostasis, and disease genesis [2, 3]. Initially identified as innate immunophagocytic sentinel cells, macrophages are now known for their remarkable plasticity, capable of dynamically polarizing into proinflammatory (M1) or antiinflammatory (M2) states in response to environmental cues. Over the past two decades, advancements in molecular biology and single‐cell histology have unveiled the complexity of macrophage signaling networks. Through the activation of various receptors, such as Toll‐like receptor (TLR), NLR, and interleukin 1 receptor (IL‐1R), macrophages detect pathogen‐ or injury‐associated molecules and modulate immune responses via signal transduction pathways, including mitogen‐activated protein kinase (MAPK), nuclear factor kappa B (NF‐κB), and JAK–STAT. Macrophages play a crucial role in coordinating immune responses, maintaining homeostasis, participating in angiogenesis and neuronal networks, and driving cancer progression. They are involved in immune diseases and contribute to cardiovascular and other pathological processes. These studies highlight the essential functions of macrophages in health and offer new insights into their roles in chronic inflammation, cancer, autoimmunity, and other diseases.

Macrophages express multiple pattern‐recognition receptors (PRRs), regulatory receptors, chemotaxis/activation‐associated cytokine receptors, antigen‐processing and presentation molecules, and the characteristic CD14 surface marker. These receptors have multiple functions, including phagocytosis, involvement in inflammatory responses, antigen processing and presentation, and immunomodulation. However, despite significant advancements in mechanistic studies, the translation of these findings to clinical practice remains fragmented, underscoring the necessity for a systematic integration of macrophage signaling pathways and their complex roles. The development of therapeutic strategies targeting macrophages is motivated by their dual roles in both protection and destruction within various disease contexts. For example, the dysregulation of signaling pathways, including TLR, JAK–STAT, PI3K–AKT, and NF‐κB, is closely associated with chronic inflammatory diseases such as rheumatoid arthritis (RA) and atherosclerosis, as well as with the immunosuppressive effects mediated by tumor‐associated macrophages (TAMs) [4, 5]. A thorough examination of the association between these pathways and the macrophages and the complex roles of these pathways and macrophages, this review provides insights into macrophage signaling pathways and their roles in health and disease, particularly the key molecular mechanisms involved in the regulation of their polarization and function. The focal point of this study will be the examination of the impact that disparate signaling pathways exert on the polarized state of macrophages. Additionally, this review will discuss the functional effects of these pathways in various diseases, including inflammatory responses, tumor immunity, and autoimmunity. Furthermore, it will introduce the potential clinical applications of macrophage signaling pathways, with a particular emphasis on developing novel therapeutic strategies that target these pathways.

The review systematically integrates the environment‐dependent activation features of macrophage signaling pathways in physiology and pathology, providing a comprehensive overview. By combining preclinical and clinical evidence, it identifies interferable targets (e.g., colony stimulating factor 1 receptor, STAT3, NLRP3 inflammatory vesicle inhibitors) and explores their potential application in clinical therapy. The study aims to provide a theoretical basis and experimental support for future disease intervention and individualized therapy.

## 2 Overview of Macrophage Biology

Macrophages can be classified into two distinct types: tissue‐resident macrophages (TRMs) and monocyte‐derived macrophages (MoMFs) [6, 7, 8]. The prevailing perspective suggests that macrophages originate from the mononuclear phagocyte system [9] and that tissue macrophages are continuously replaced by monocytes in the circulation [10]. However, recent advancements in fate‐tracing animal models and single‐cell sequencing technologies have facilitated a more nuanced understanding of the origins of TRMs [11, 12]. These studies have revealed that TRMs predominantly originate from yolk sacs, fetal liver, and bone marrow hematopoietic stem cells [13]. The origins of TRMs vary slightly across different tissues and organs; some are exclusively derived from the yolk sac (e.g., brain microglia), while others arise from both the yolk sac and the fetal liver (e.g., dermal Langerhans cells, alveolar macrophages, hepatic Kupffer cells (KCs), and cardiac macrophages) [14]. However, intestinal and cutaneous dermal macrophages are derived primarily from bone marrow‐derived monocytes and are continuously replenished after birth by circulating monocytes [15, 16, 17].

**FIGURE 1:**
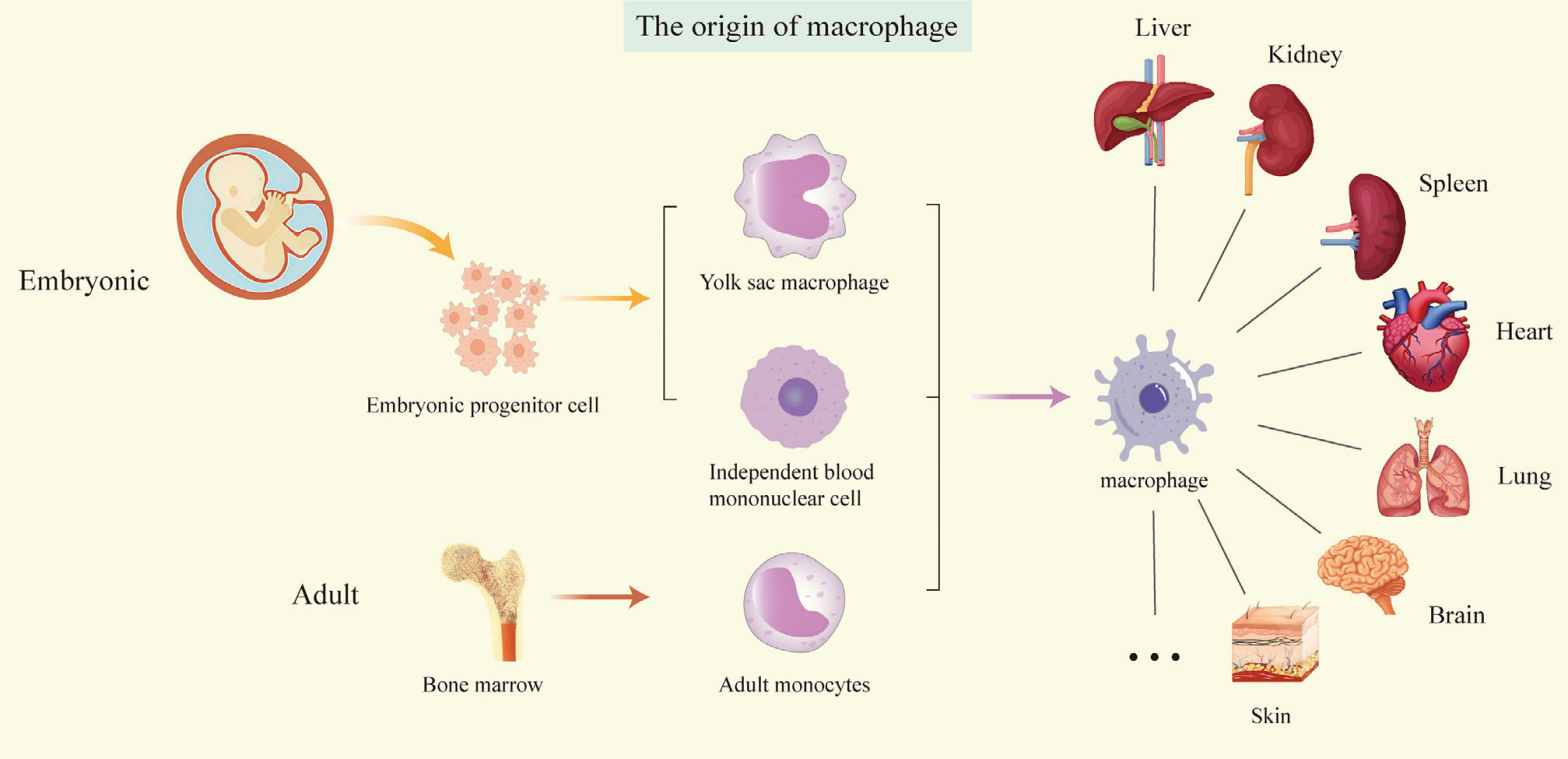
The M1/M2 macrophage origin. Macrophage ontogeny primarily originates from embryonic precursor populations, which include yolk sac‐derived macrophages, autonomous blood monocytes, and bone marrow‐derived adult monocytes. These progenitor cells disseminate throughout the circulatory system to various organ systems, including hepatic, renal, splenic, cardiac, pulmonary, cerebral, and cutaneous tissues, where they undergo terminal differentiation into specialized tissue‐resident macrophage subsets.

The role of TRMs in antitumor effects is multifaceted, exhibiting both pro‐ and antitumorigenic functions. As demonstrated by María Casanova‐Acebes et al., TRMs enhance tumor immunity and response to immunotherapy, promote epithelial–mesenchymal transition (EMT) and invasiveness of tumor cells, induce potent regulatory T‐cell (Treg) responses, and shield tumor cells from adaptive immune attack [18]. Additionally, TRMs gather near tumor cells during the early stages of tumor formation [3] (Figure 1).

### 2.1 Macrophage Plasticity and Polarization

Macrophages are a key type of immune cells that fulfill a dual role in the inflammatory response by transitioning between proinflammatory and antiinflammatory subtypes, depending on the prevailing context.

#### 2.1.1 M1 Macrophages

When exposed to Th1 cytokines such as interferon ‐ γ (IFN‐γ), tumor necrosis factor ‐ α (TNF‐α), and IL‐2 or lipopolysaccharide (LPS), macrophages undergo a shift toward the M1 phenotype. Metabolically, glycolysis is a major source of energy. Stimulation of LPS leads to proinflammatory polarization of macrophages and a pro‐Th1 cellular response accompanied by metabolic reprogramming [19], characterized by increased aerobic glycolysis and disruption of the tricarboxylic acid (TCA) cycle. In contrast, CD40 signaling has been shown to promote proinflammatory and antitumor polarization through an as yet poorly understood metabolic programming involving fatty acid oxidation without inhibiting TCA cycle, suggesting therapeutic potential [20]. A number of factors contribute to M1 polarization, including LPS, IFN‐γ, and TNF‐α. M1 macrophages are polarized through a variety of signaling pathways, such as NF‐κB, interferon regulatory factors (IRFs), PI3K/Akt, and STAT1 [21, 22]. Based on our previous studies, macrophages can be converted to an M1 phenotype under the influence of the action of FSTL1 and binding to PKM2, thereby attenuating liver inflammation and fibrosis [23].

#### 2.1.2 M2 Macrophages

In addition, as a reply to Th2‐type cytokines, macrophages are known to polarize toward the M2 phenotype, such as IL‐4, IL‐10, and IL‐13, as well as certain TLR ligands. Metabolically, oxidative phosphorylation (OXPHOS) dominates in these cells. M1 macrophages are known to inhibit tumor growth, whereas specific subpopulations of M2 macrophages have been observed to promote tumor progression, metastasis, angiogenesis, and tissue repair. The M2 macrophage population has been further classified into M2a, M2b, M2c, and M2d subtypes.

##### 2.1.2.1 M2a

M2a macrophages represent a prominent line of research within the broader category of M2 macrophages [24]. The initial identification of these cells was made in a 1992 study that demonstrated increased activity and CD206 expression in surface mouse peritoneal macrophages stimulated by IL‐4 [25]. The macrophage‐polarized M2a phenotype is characterized by elevated expression levels of cell‐surface markers, notably CD206, in response to cytokines such as IL‐13. In contrast, the expression levels of CD163 and CD86 are comparatively low to moderate [26]. Additionally, M2a cells have been shown to produce various cytokines, including IL‐10, TGF‐β, chemokine (C‐C Motif) Ligand (CCL17, CCL18, CCL22, and CCL24) [24, 25, 27].

Additionally, M2a macrophages have been demonstrated to exhibit profibrotic functions [28]. The polarization of M2a macrophages is facilitated by signaling pathways mediated by PI3K/Akt and STAT6. Macrophages express both type I and type II IL‐4 receptors. The activation of these receptors leads to the phosphorylation and subsequent dimerization of STAT6. Once activated, the STAT6 dimer translocates to the nucleus, where it initiates the expression of target genes. In contrast, type I IL‐4 receptors activate only insulin receptor substrates (IRSs), which do not translocate to the nucleus [24]. However, activated IRSs have been observed to initiate signaling pathways, including PI3K/Akt‐mediated signaling pathways. The STAT6 protein has been demonstrated to regulate the expression of genes related to M2 polarization, a downstream target of peroxisome proliferators‐activated receptor (PPAR‐γ), and to have a positive regulatory role [29].

##### 2.1.2.2 M2b

The binding of LPS to antiovalbumin IgG/OVA or antisheep erythrocyte IgG/erythrocyte immune complexes (ICs) has been shown to promote a shift from M1 to M2 in the macrophage phenotype, characterized by a decrease in IL‐12 production and an increase in IL‐10 levels. The distinctive M2 subtype, initially described in 2002, has been designated as M2b, and LPS + IC has been identified as a conventional M2b inducer [30]. M2b macrophages are distinguished by their unique features that set them apart from other M2 subpopulations. They are capable of transferring the Th1‐cell response to that of Th2‐cells, primarily through the secretion of IL‐4 and IL‐13. A subsequent study revealed that whole‐body irradiation‐induced miR‐122 decreased GAS5 expression, leading to increased CCL1 levels and promoting macrophage conversion to the M2b phenotype, although the mechanism of radiation‐induced miR‐122 remains to be elucidated [31].

M2b cells are distinguished by their elevated CCL1 production, a characteristic that distinguishes them from other M2 cell types. CCL1 interacts with the cell surface receptor CCR8, attracting monocytes, dendritic cells, and natural killer (NK) cells. The release of CCL1 is crucial for the maintenance of M2b identity, as its inhibitory effect can lead to the transformation of M2b cells into M0 or M1 macrophages [32]. Other markers associated with M2b macrophages include CD86, IL‐10, and TNF‐α. CD86, also known as B7‐2, serves as a marker for both M1 and M2b macrophages, allowing M2b to differentiate from other M2 macrophages. Conversely, IL‐10's efficacy in differentiating M2b from other subtypes is limited due to its coproduction by M2c and M2d cells [33]. Finally, although TNF‐α is recognized for its paradoxical antiinflammatory effects, it has been identified as a marker for M2b in several studies [30].

##### 2.1.2.3 M2c

IL‐10, TGF‐β, and glucocorticoids stimulate M2c macrophages, leading to increased expression of CD163, TLR1/8, and the Tyro3–Axl–MerTK pathway, while decreasing CD86 levels. These macrophages secrete IL‐10, TGF‐β, CCL16, CCL18, and CXCL13 [34, 36]. M2c macrophages are polarized through STAT3, MAPK/ERK, and PI3K/Akt mediated signaling pathways. GAS6, produced by M2c macrophages, interacts with MerTK to induce IL‐10 production, which further activates M2c macrophages, creating a positive feedback loop that enhances M2c polarization. This polarization is critical for regulating tissue repair, as signaling through these pathways leads to the activation of multiple genes associated with anti‐inflammation, matrix remodeling, angiogenesis, and phagocytosis [30, 37]. Furthermore, M2c macrophages possess the capacity to stimulate and induce Treg in both in vitro and in vivo assays, indicating that the effects of M2c macrophages are mediated by Treg [37].

##### 2.1.2.4 M2d

The initial identification of M2d macrophages was made in the ascites fluid of ovarian cancer patients in 2007. These cells are distinguished by their low expression of CD86 and high expression of CD163, and upon stimulation with LPS, IL‐6, and A2R ligands, they produce M‐CSF, IL‐10, and CCL18. The binding of IL‐6 to the IL‐6 receptor/gp130 receptor complex initiates the recruitment of JAK1/2, leading to the phosphorylation of STAT3. This process results in the dimerization of STAT3, which subsequently translocates to the nucleus to activate gene transcription. Leukemia inhibitory factor, a member of the IL‐6 family, induces the production of IL‐6 by binding to its receptor. This mechanism allows M2d macrophages to utilize IL‐6 in an autocrine manner [38]. Additionally, M2d macrophages are involved in tumor progression, angiogenesis, and extracellular matrix (ECM) remodeling through the secretion of IL‐10, TGF‐β, vascular endothelial growth factor (VEGF), and matrix metalloproteinase 9 (MMP9), while exhibiting low levels of IL‐12, TNF, and IL‐1β expression [39]. Moreover, other macrophage subpopulations, including Mhem, Mox, M4, and MHb, have been postulated (Table 1).

**TABLE 1:** Differential macrophage subtypes and functions.

Macrophage subset	Stimuli	Markers	Secreted molecules	Functions	References
M1	IFN‐γ, LPS, CD40	MHCII, CD40, CD68, CD80, CD86, TLR2/4, IL‐1R	TNF‐α, IL‐1α, IL‐1β, IL‐6, IL‐12, IL‐23, COX‐2, iNOS	Proinflammation, pro‐Th1 cellular response, inhibit cancer growth	[19, 20, 21, 22, 23]
M2a	IL‐4, IL‐13	CCL17, CD206, CD209, HLA‐DR, Dectin‐1	IL‐10, TGF‐β, CCL17, CCL18, CCL22, CCL24	Anti‐inflammation, tissue repair, metastasis	[24, 25, 26, 27, 28]
M2b	LPS + ICs	CD86, CCL1, SPHK1, TNF‐α	IL‐10, SPHK1, CCL1, TNF‐α,	Immunoregulation, tumor progression	[30, 31, 32, 33]
M2c	IL‐10, TGF‐β, glucocorticoids	CD163, TLR1/8, Tyro3–Axl–MerTK	IL‐10, TGF‐β, CCL16, CCL18, CXCL13	Immunosuppression, phagocytosis, angiogenesis	[30, 34–37]
M2d	LPS, IL‐6/A2R ligands	CD163	IL‐10, IL‐6, CCL18, M‐CSF	Tumor progression, angiogenesis	[38, 39]

In summary, macrophages, as a critical component of the immune system, can exhibit a dual role in inflammatory regulation by transitioning between proinflammatory M1 and antiinflammatory M2 subtypes. M1 macrophages are formed under the stimulation of Th1 cytokines or LPS and are powered by glycolysis and polarized through various signaling pathways. In contrast, M2 macrophages respond to Th2 cytokines and are iso‐polarized, predominantly oxidative phosphorylated, and subdivided into subtypes such as M2a, M2b, M2c, and M2d, each with unique markers and polarization signaling pathways. M2 macrophages respond to Th2‐type cytokine iso‐polarization by oxidative phosphorylation, which is subdivided into four subtypes, each with unique markers, functions, and polarization signaling pathways. The polarization process of these subtypes involves specific signaling pathways.

## 3 Macrophage Signaling Pathways

**FIGURE 2:**
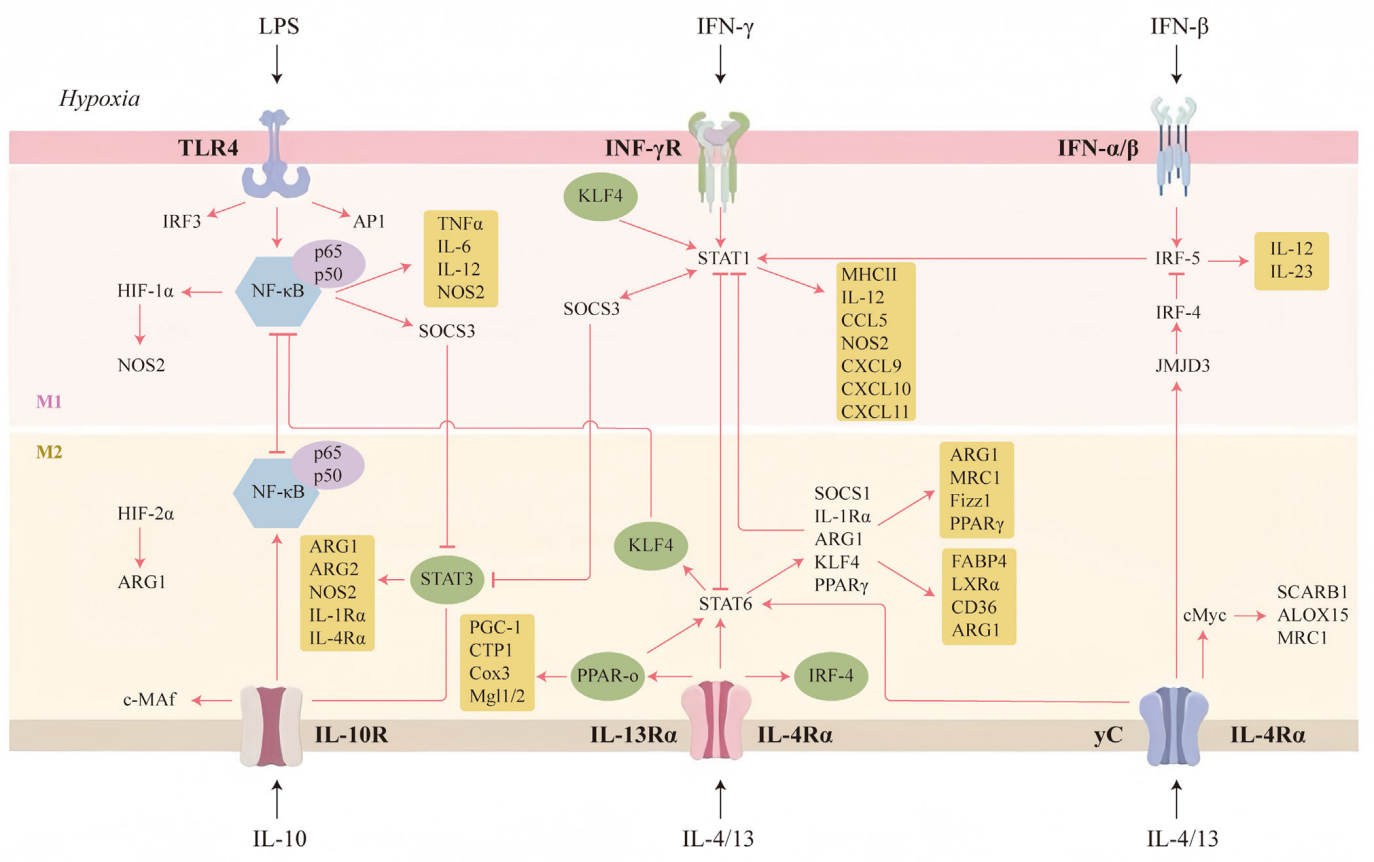
Cytokines and signaling pathways in the regulation of macrophage polarization. Exogenous stimuli, such as LPS and IFN‐γ, along with endogenous signals like IFN‐β, in conjunction with metabolic stressors such as hypoxia, initiate signaling cascades through the engagement of PRRs, particularly TLR4 activation. This molecular interplay orchestrates the transcriptional activation of pivotal regulators, including IRFs, NF‐κB, and STAT family members. Collectively, these factors serve as molecular determinants of macrophage differentiation, guiding cellular commitment toward either M1 proinflammatory or M2 immunoregulatory polarization states. Furthermore, the figure delineates the specific functions of various M2 macrophage subsets (M2a, M2b, M2c, M2d) and their associated molecules, thereby clarifying their distinct roles in immune responses and tissue repair.

Macrophage polarization phenotype and functional regulation are contingent on the integrated action of complex signaling networks. Among them, core pathways such as NF‐κB, JAK/STAT, PI3K/Akt, and MAPK regulate key transcription factors through cascade reactions, forming molecular switches for different polarization phenotypes. The molecular mechanisms of the signaling networks and their interactions require systematically analyzed (Figure 2).

### 3.1 Classical Polarization Regulatory Pathways

#### 3.1.1 JAK–STAT Pathway

The JAK–STAT signaling pathway represents a pivotal signal transduction pathway for the activation of cytokines (e.g., IL‐4, IL‐6, IFN‐γ, etc.), and its core mechanism involves the triggering of the phosphorylation of the JAK kinase upon the binding of transmembrane receptors to ligands. This, in turn, activates the STAT proteins to form a dimer and enter the nucleus to regulate the expression of target genes [40]. In the context of macrophage polarization, this pathway exerts a regulatory role in M1 and M2 phenotypic transitions through the activation of distinct STAT isoforms. For instance, IFN‐γ drives M1 polarization and induces the secretion of proinflammatory factors such as TNF‐α and IL‐6 through the activation of STAT1. In contrast, IL‐4 and IL‐13 upregulate anti‐inflammatory markers, including arginase 1 (Arg1) and YM1, via STAT6. Additionally, IL‐10 promotes M2 polarization and inhibits inflammatory responses through the action of STAT3 [41]. Differential activation of STAT is the molecular basis of macrophage functional plasticity, which directly affects the dynamic balance of inflammatory diseases and tumor microenvironment (TME).

It has been established that STAT1 and STAT5 are core drivers of M1 polarization. JAK1 and JAK2 are activated by the binding of IFN‐γ to its receptor, which then leads to the phosphorylation of STAT1 and the formation of a homodimer that directly binds to the promoter regions of proinflammatory genes (e.g., NOS2), while repressing M2‐associated gene expression [42]. In contrast, STAT5 enhances the antigen‐presenting ability of macrophages by regulating the secretion of cytokines such as IL‐12. Finally, STAT3 and STAT6 dominate M2 polarization. Notably, IL‐4/IL‐13 upregulates markers such as CD206 and Arg1 through the activation of STAT6, thereby promoting tissue repair. In addition, IL‐10 induces STAT3 suppressor of cytokine signaling 3 (SOCS3) expression, creating a negative feedback loop to inhibit JAK1 activity, thus suppressing M1 polarization and enhancing immune tolerance. The sustained activation of STAT3 is also associated with a protumorigenic phenotype in TAMs promoting angiogenesis and immune escape through the secretion of factors such as VEGF, TGF‐β, and so on [43, 44].

#### 3.1.2 NF‐κB Pathway

NF‐κB is a family of pleiotropic transcription factors involved in numerous physiological and pathological processes by regulating inflammation, immune responses, cell proliferation, and apoptosis. The family members include RelA (p65), RelB, c‐Rel, p50 (derived from p105), and p52 (derived from p100) [45], all of which have Rel homology domains that function through the formation of heterodimers or homodimers, and whose classical and nonclassical activation pathways play different roles in macrophage polarization. The classical pathway is triggered by TLR4/LPS, TNF‐α, and so on, which degrades IκB protein through phosphorylation by IKK complex [46, 47, 48], releasing the p50–RelA dimer into the nucleus, driving the expression of proinflammatory genes (e.g., inducible nitric oxide synthase [iNOS], IL‐6), and directly promoting the polarization of M1‐type macrophages. In contrast, the nonclassical pathway is activated by stimuli such as CD40 and BAFF‐R, and it relies on NIK kinase to process p100 into p52. This processed p52 then binds to RelB, thereby regulating genes associated with lymphoid organ development. While it can be hypothesized that the nonclassical pathway may play a role in the regulation of M2‐type polarization, its precise function remains to be elucidated.

In M1‐type polarization, NF‐κB activation is closely associated with glycolytic metabolic reprogramming. For instance, enhanced NF‐κB signaling in BCL3 knockout macrophages resulted in the upregulation of glycolysis rate‐limiting enzymes (e.g., HK2, LDHA), along with a significant increase in M1 markers (IL‐6, iNOS) [49], suggesting that NF‐κB reinforces the proinflammatory phenotype by activating glycolysis. In addition, the TLR/NF‐κB signaling pathway plays a crucial importance in the polarization of macrophages. TLR, a member of the PRR family, recognizes pathogen‐associated molecular patterns (PAMPs) and is integral to the immune response to pathogens. Phosphorylation of MyD88 is the first step in TLR signaling, but TLR3 operates through a TRIF‐dependent pathway [50, 51]. Subsequent to this, TLR activation triggers the expression of various downstream factors, including NF‐κB, AP1, CREB, and IRF3/7, which subsequently facilitate the transcription of genes associated with inflammatory factors, adhesion molecules, and other substances [51, 52]. M1 polarization can be induced by bacterial LPS or viral RNA, both of which activate the TLR/NF‐κB pathway and lead to the expression of proinflammatory cytokines.

The effects of NF‐κB signaling pathway on macrophage M2 polarization are multifaceted and environment‐dependent. During the process of M2 polarization, NF‐κB activation by IL‐1β induces the expression of the STAT6‐dependent factor VEGF‐A, which promotes tissue repair. Conversely, the antiinflammatory properties of macrophages can be enhanced by inhibiting NF‐κB phosphorylation, for instance, with a JSH‐23 inhibitor [53]. The term “LPS‐tolerant” refers to a specific type of macrophages characterized by diminished restimulation potential, which are thought to exhibit M2‐like properties. These macrophages are distinguished by the accumulation of (inhibitory) p50 NF‐κB homodimers. Research has demonstrated that the inhibition of the p50 NF‐κB subunit can hinder the progression of tolerance and the expression of cytokines associated with the M2 phenotype [54]. The NF‐κB pathway is a central hub for macrophage polarization through a dual mechanism of inflammatory signaling dominance and metabolic regulation. Its targeted intervention (e.g., inhibition of the classical pathway or modulation of metabolic enzymes) may develop a novel strategy for the management of inflammatory diseases.

#### 3.1.3 PI3K–Akt–mTOR Pathway

The PI3K/Akt signaling pathway is critically important, as its activation leads to the phosphorylation of tyrosine residues, facilitates various signaling processes for extracellular cytokines and other signals in the ECM, and ultimately enhances cell viability while inhibiting cellular senescence and death [55, 56]. Moreover, this pathway plays a pivotal role in regulating various aspects of macrophages, including survival, migration, proliferation, and the coordination of their responses to diverse metabolic and inflammatory signals [55, 57]. Multiple studies have consistently emphasized the pivotal function of PI3K/Akt in macrophage polarization and exosome promotion of the transition of M1 macrophages to M2 through the PI3K/Akt pathway [58, 59]. Researchers developed a novel therapeutic nanofiber based on rhubarbic acid, a unique drug that activates the PI3K/AKT/mTOR pathway, inhibits the NF‐κB and STAT3 pathways, and progresses the conversion of M1 macrophages to M2, leading to effective treatment of retinal ischemia/reperfusion injury [60]. Another significant finding is the role of Saikosaponin D, which was shown to downregulate STAT6 and PI3K/AKT/mTOR pathways in IL‐4‐stimulated RAW 264.7 cells, thereby inhibiting cellular M2 polarization [61].

#### 3.1.4 IRFs Family

An important family of transcription factors is known as the IRFs, comprising 9 members (IRF1‐9) in humans, with IRF3, IRF5, and IRF7 exhibiting particularly notable functions in innate immunity and macrophage polarization [62, 63, 64], characterized by a conserved N‐terminal DNA‐binding domain featuring a unique “tryptophan pentad repeat” structure that recognizes interferon‐stimulated response elements in target gene promoters. Functionally, IRFs are pivotal regulators of innate and adaptive immunity, particularly in mediating IFN‐α/β responses, antiviral defense, and inflammation.

In macrophages, IRFs play a crucial role in maintaining immune homeostasis by regulating the polarization phenotypes and functional plasticity of these cells. For instance, IRF5 has been shown to drive the expression of proinflammatory factors such as IL‐12 and TNF‐α in M1‐type macrophages, while IRF4, in conjunction with STAT6, has been observed to synergistically induce an antiinflammatory/restorative phenotype (e.g., upregulation of IL‐10, Arg1) in M2‐type macrophages [65]. Additionally, IRF3 and IRF7 are significantly important in regulating immune responses. IRF1 and IRF8 are known to have complex functions that reinforce NF‐κB‐mediated inflammatory signaling while also mediating the virally triggered type I interferon response [66]. A functional imbalance of IRFs (e.g., IRF5 overexpressing systemic lupus erythematosus (SLE), IRF4 defects and immune dysregulation) has been shown to lead to aberrant macrophage activation and promote autoimmune diseases, chronic inflammation, and tumor progression. It has become a core target of the macrophage immunoregulatory network by integrating signaling pathways such as JAK–STAT and TLR [67].

IRF5 has been shown to possess a multitude of functions, such as activating genes that encode inflammatory cytokines, type I interferons, and tumor suppressors. Recently, IRF5 has been recognized as a crucial transcription factor in M1 macrophage the differentiation of M1 macrophages because M1 macrophages express IRF5 in large numbers, and the expression of IRF5 in M2 macrophages is associated with the overall expression of genes characteristic of M1 macrophages [68]. Furthermore, research indicates that the knockdown of IRF5 in iNOS^−/−^ M1 macrophages results in a decreased expression of M1‐characteristic cytokines. These findings identify tyrosine residues 74th and 104th of IRF5 as being critical for inducing IL‐12/p40 promoter activation. This research unveils a novel mechanism that regulates M1 macrophage differentiation through nitration of IRF5 tyrosine residues [69].

Additionally, evidence suggests that IRF4 functions as a negative regulator of inflammation in adipose tissue macrophages during diet‐induced obesity, partially by modulating M2‐like macrophage polarization [70]. Consequently, distinct isoforms of IRF evidently exhibit varied roles in regulating macrophage phenotypes.

#### 3.1.5 Notch Signaling Pathway

The Notch signaling pathway, which is highly conserved in both vertebrates and invertebrates, significantly influences various aspects of postnatal development and tissue renewal. It plays a crucial role in regulating cell fates during proliferation, differentiation, and apoptosis [71, 72, 73]  . Mammals express four transmembrane Notch receptors (Notch1 to Notch4) and five classical transmembrane ligands, divided into two families: Delta‐like 1, 3, and 4, and the JAG‐type ligands Jagged‐1 and Jagged‐2. These homologous genes exhibit highly conserved structural domain architectures with high structural fidelity. Notch proteins represent a family of single‐pass transmembrane receptors, distinguished by three primary modular components: the Notch extracellular domain, the Notch intracellular domain, which contains essential regulatory motifs such as ankyrin repeats and a transactivation domain [74, 75]. The cellular specificity and spatial distribution patterns of both Notch receptors and their corresponding ligands fundamentally regulate the dynamics of signal transduction, acting as critical determinants of signaling amplitude and temporal duration. Additionally, RBP‐J plays a pivotal role in orchestrating context‐dependent transcriptional regulation.

Macrophages have been shown to stably express Notch ligands and receptors Notch1, 2, and 4, suggesting a potential role for Notch signaling in the activation of these cells and the regulation of their multifaceted biological properties [76]. Subsequent studies have confirmed that LPS can specifically enhance the expression of Notch1 through the activation of macrophage pathways that are either MyD88‐dependent or independent. This activation leads to the expression of downstream genes. When Notch signaling is activated, there is an increase in the secretion of inflammatory factors such as IL‐6 and iNOS, a reduction in the release of IL‐10, and a polarization of macrophages toward the M1 phenotype [77]. In a subsequent study, Keewan and Naser [78] investigated the roles of RBP‐J‐mediated Notch signaling and TLR signaling as essential regulators of macrophage function, demonstrating a synergistic regulatory effect on these cells. Feedback mechanisms, which follow the expression of downstream genes Hes1 and Hey1, modulate the regulatory influence of Notch signaling on macrophages.

Xu et al. [79] clearly demonstrated that the RBP‐J‐dependent classical Notch pathway activates the TLR4 molecule, which is activated via the MAP kinase‐interacting kinase–eIF4E–interleukin‐1 receptor. The activation of the TLR4 molecule by the RBP‐J‐dependent classical Notch pathway is associated with the MAP kinase‐interacting kinase–eIF4E–IRAK2 pathway. This pathway has been shown to induce high expression of IRF8, which activates the expression of molecular markers related to M1 polarization. Notably, this pathway has been shown to progress M1 inflammatory responses in macrophages.

In the study by Wang et al. [80], it was observed that SOCS3 functions as a downstream molecule of the Notch signaling pathway, regulating the polarization of macrophages. The RBP‐J‐mediated Notch signaling pathway has been found to regulate M1 and M2 macrophages through SOCS3. Emerging evidence from recent investigations highlights the pivotal regulatory role of the Notch–RBP‐J signaling axis in orchestrating lineage commitment, spatial distribution, and effector functions of distinct hepatic macrophage subsets during metabolic dysfunction‐associated steatohepatitis (MASH). This offers novel mechanistic insights into myeloid cell dynamics under inflammatory conditions. These findings propose that pharmacological modulation of RBP‐J‐mediated transcriptional programs could serve as a potential strategy for mitigating disease progression in the pathogenesis of MASH [81]. Complementary research further elucidates the capacity of Notch‐mediated signaling to influence macrophage polarization states in hepatic disorders, particularly concerning the remodeling of the inflammatory microenvironment [82]. Xu et al. [83] utilized a combined obesity‐ethanol steatohepatitis murine model to demonstrate a concurrent elevation of Notch1 receptor activation and proinflammatory M1‐polarization markers within liver‐resident macrophages. In contrast, genetic ablation of Notch1 resulted in an inability to upregulate M1‐associated transcriptional signatures in hepatic myeloid populations.

Collectively, the functional polarization and effector activities of macrophages are coordinated through the interplay of multiple signaling cascades. These cascades include key regulators such as the JAK–STAT axis, the NF‐κB network, the PI3K–Akt–mTOR pathway, the IRF transcription factor family, and Notch‐mediated signaling. Notably, the JAK–STAT cascade plays a pivotal role in determining M1/M2 phenotypic commitment through the differential phosphorylation of STAT1 and STAT6 isoforms. In contrast, the NF‐κB system exerts a dual regulatory influence over inflammatory responses and tissue repair mechanisms, which is mediated through its canonical and alternative activation routes, respectively. The PI3K–Akt–mTOR pathway exerts a crucial function in metabolic reprogramming, while the IRF5/8 and IRF4/3 pathways exhibit an antagonistic relationship in regulating inflammatory and antiinflammatory genes. The Notch signaling pathway, through its interaction with the RBP‐J axis, contributes to M2‐like polarization within the microenvironment, thereby facilitating tumor development. The intricate interplay among these pathways is crucial for maintaining physiological homeostasis, with their imbalance potentially leading to various pathologies, including tumors, neurodegenerative diseases, and autoimmune diseases.

## 4 Divergent Functions of Macrophages

### 4.1 Phagocytosis

**FIGURE 3:**
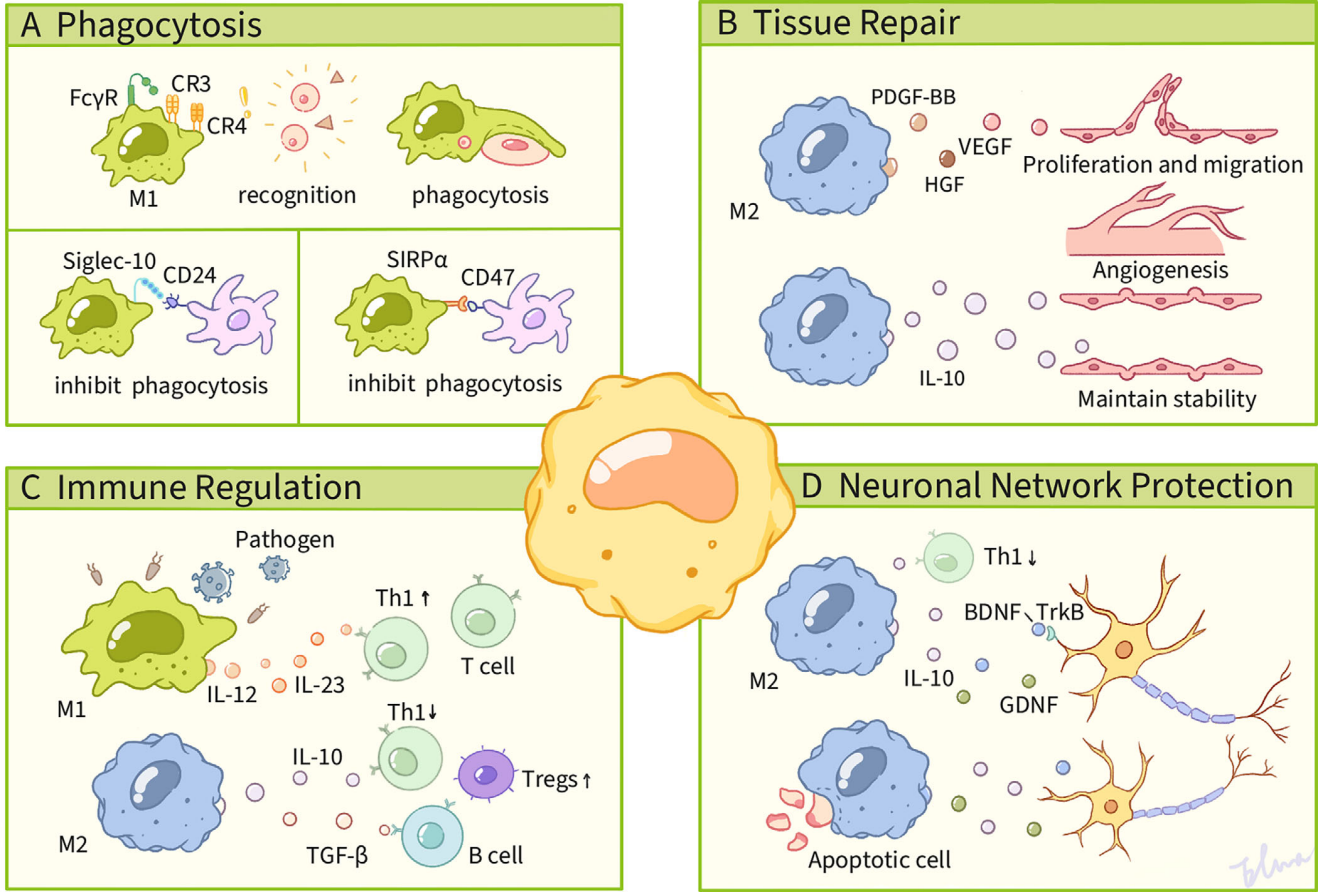
Macrophage functions in phagocytosis, tissue repair, immune regulation, and neuronal network protection. The mechanisms of macrophage action in various functional states can be categorized into four main processes: (A) phagocytosis, (B) tissue repair, (C) immune regulation, and (D) neuronal network protection. M1‐type macrophages identify and engulf pathogens through FcγR, CR3, and CR4 receptors, while secreting cytokines such as IL‐12 and IL‐23 to enhance Th1‐type T‐cell activation. In contrast, M2‐type macrophages facilitate tissue repair and angiogenesis through factors including PDGF‐BB, VEGF, and HGF, while also maintaining immune homeostasis via IL‐10. Additionally, M2‐type macrophages provide protection to neuronal networks and suppress Th1‐type T‐cell activity through neurotrophic factors like BDNF and GDNF. Moreover, the Siglec‐10/CD24 and SIRPα/CD47 signaling pathways have been implicated in the inhibition of phagocytosis, with Tregs and B cells recognized as significant contributors to immunoregulation.

As myeloid‐derived immune sentinels, macrophages perform dual immunological roles as professional phagocytes and antigen‐presenting cells. These versatile cells orchestrate the elimination of pathogens, initiate inflammatory cascades, and clear cellular debris through their engulfment processes. Recent research underscores their essential contributions to tissue development and metabolic homeostasis, mediated by finely tuned phagocytic activity and cytokine‐mediated intercellular communication. Phagocytosis, a specialized mechanism of engulfment, enables both professional phagocytes (e.g., macrophages) and nonspecialized cell types (e.g., epithelial cells) to detect, internalize, and degrade particulate matter larger than 0.5 µm in diameter, serving as a critical defense strategy for maintaining homeostatic equilibrium during pathogenic incursions [84]. The ability of macrophages to recognize foreign pathogens or foreign objects is based on multiple receptors (e.g., FcγR) on their surface. Among them, PRRs play a critical role. These immune surveillance receptors specifically detect PAMPs—evolutionarily conserved structural motifs exclusive to microbial pathogens., such as LPS and peptidoglycan in bacteria, and nucleic acids in viruses. In addition to these mechanisms, macrophages can recognize pathogens labeled by complement proteins via complement receptors (CR). When the complement system is activated, complement components, such as C3b, are deposited on the surface of pathogens, and macrophage CRs (e.g., CR3, CR4) recognize these markers, thereby initiating phagocytosis. A 2007 investigation by Green and colleagues [85] demonstrated the recruitment of autophagy‐related (Atg) proteins, including Beclin 1, LC3, Atg5, and Atg7, to maturing phagosomes in macrophages following TLR‐mediated signaling. This phenomenon was subsequently characterized as the noncanonical LC3‐associated phagocytosis pathway, which is distinct from classical autophagy processes. In addition, recent findings have demonstrated that rhamnose, produced by prokaryotic metabolism, promotes SLC12A4 activation through binding to SLC12A4. This, in turn, induces modulation of Rac1 and CDC42 activity and promotes phagocytosis by macrophages, thereby contributing to the alleviation of sepsis [86] (Figure 3).

The macrophage‐expressed inhibitory receptor Siglec‐10 engages with tumor‐associated CD24 through interactions at its extracellular domain, which triggers intracellular signaling via immunoreceptor tyrosine‐based inhibitory motifs (ITIMs) located within its cytoplasmic tail [87]. This molecular recognition event facilitates the recruitment of Src‐family tyrosine kinases (SFKs) that phosphorylate the ITIM tyrosine residues, thereby initiating a phosphorylation cascade that mobilizes Src homology 2 (SH2) domain‐containing phosphatases, including SHP‐1 and SHP‐2. The subsequent binding of SHP‐1 to the phosphorylated ITIM domains mediates substrate dephosphorylation through its catalytic activity, ultimately leading to actin cytoskeletal reorganization that enables phagocytic clearance. Additionally, SHP‐1 negatively regulates intracellular signaling involving cell adhesion molecules, ECM factors, hormones, cytokines, and growth factors [88]. Consequently, the interaction of CD24 with Siglec‐10 impedes phagocytosis by macrophages and fosters evasion from tumors. Blocking CD24 or Siglec‐10 expression by gene or antibody facilitates macrophage phagocytosis and inhibits tumor growth [89, 90].

SIRPα serves as the endogenous ligand for CD47 [91], a surface‐expressed transmembrane glycoprotein predominantly localized on myeloid lineage cells, including macrophages, monocytes, and dendritic cells. The architecture of the SIRPα receptor consists of an extracellular region with three immunoglobulin‐like domains, a single transmembrane module, and cytoplasmic tyrosine kinase phosphorylation motifs. Essential to its inhibitory function, the intracellular segment contains ITIMs that mediate the suppression of signal transduction [92, 93]. This counter‐regulatory mechanism operates through the competitive antagonism of ITAM‐coupled receptor activation. Effective signal inhibition requires the phosphorylation of ITIM tyrosine residues within cytoplasmic sequences, which facilitates the recruitment and enzymatic activation of SH2 domain‐containing phosphatases SHP‐1 and SHP‐2. These effector molecules, equipped with SH2 phosphotyrosine‐binding modules, execute downstream dephosphorylation cascades [92, 93]. The enzymatic activation of SHP‐1 and SHP‐2 phosphatases triggers the phosphorylation of myosin IIA, acting as a molecular switch that antagonizes nonmyosin IIA activity. These nonmuscle myosin isoforms play a critical role in regulating phagocytic machinery by coordinating phagolysosome maturation and orchestrating target engulfment in macrophages. When myosin IIA is dephosphorylated in macrophages, actin depolymerization occurs, resulting in reduced phagocytosis [94, 95]. The CD47–SIRPα interaction between malignant cells and phagocytic effector cells initiates tyrosine phosphorylation of ITIMs within SIRPα’s cytoplasmic domain through SFK‐mediated activation. This signaling cascade facilitates recruitment of protein tyrosine phosphatases SHP‐1/SHP‐2, which contributes to reduced phagocytosis [96].

### 4.2 Tissue Repair

Tissue regeneration and repair are fundamental biological mechanisms crucial for maintaining organismal homeostasis and ensuring survival [97]. Following tissue damage caused by pathogen invasion or physical trauma, necrotic cells release endogenous damage‐associated molecular patterns (DAMPs), while invading microbes secrete PAMPs. Together, these signals activate innate immune signaling cascades that drive inflammatory responses [98]. This molecular alert system mobilizes a complex inflammatory cascade characterized by the sequential recruitment, clonal expansion, and functional maturation of diverse effector populations. These include immune effectors such as neutrophils, macrophages, innate lymphoid cells, NK cells, and adaptive lymphocytes, as well as stromal components like fibroblasts, epithelial and endothelial lineages, and progenitor cells. Collectively, these elements establish an integrated cellular network that promotes regenerative processes [99]. Under physiological conditions, a self‐limited inflammatory phase facilitates the precise architectural restoration of native tissue matrices. In contrast, persistent dysregulation of healing mechanisms can lead to maladaptive fibroproliferative responses, which are characterized by excessive ECM deposition that compromises parenchymal functionality and may culminate in terminal organ insufficiency [100]. Therefore, the spatiotemporal regulation of repair pathways is essential. Among the various contributors to tissue restoration, macrophages are identified as pivotal regulators due to their plastic functional adaptability and context‐dependent polarization states [101]. They have been shown to play crucial regulatory roles throughout all phases of repair and fibrosis [102]. Recent strides in regenerative medicine and molecular biology have underscored the pivotal role of macrophages in promoting regeneration across diverse tissues, including the heart, liver, kidney, muscle, and nervous system. The capacity of macrophages to transition between distinct phenotypes in response to microenvironmental signals renders them promising therapeutic targets for enhancing tissue repair and regeneration [103].

The modulation of chemokine receptor signaling in mononuclear phagocytes alters their migratory patterns and functional roles in regenerative processes. Experimental evidence demonstrates that M2‐polarized macrophages act as crucial regulators of wound resolution and neovascularization [104], thereby highlighting their therapeutic potential in regenerative medicine. In contrast, clinical analyses indicate that the dynamics of TAM infiltration correlate with negative clinical outcomes in cohorts treated with antiangiogenesis therapies [105]. Yang et al. [106] established the proangiogenic functions of M2‐like macrophages through a multiplatform analysis that incorporated human pancreatic ductal adenocarcinoma specimens, murine xenograft models, and The Cancer Genome Atlas genomic datasets. In contrast to M0 macrophage‐derived exosomes (MDEs), M2 MDEs promote ex vivo and in vivo angiogenesis and tumor progression. Given that conventional anti‐VEGF drugs are often ineffective in pancreatic cancer, proangiogenic M2 MDEs associated with TAMs represent emerging therapeutic targets in pancreatic carcinogenesis. Guo et al. [107] demonstrated that Runt‐related transcription factor 1 (RUNX1) drives CCL2 expression in colorectal adenocarcinoma through transcriptional upregulation of hematological cell lineage 2 (HCL2), establishing RUNX1 as a critical transcriptional mediator of HCL2‐dependent chemokine signaling. In their study of colorectal cancer (CRC), Guo et al. [107] found that RUNX1 recruits macrophages and induces M2‐polarized TAM in CRC by promoting the production of CCL2 and the activation of the hedgehog pathway. Emerging evidence reveals macrophages induce endothelial activation through paracrine secretion of angiogenic mediators such as VEGF, driving pathological neointimal hyperplasia. Furthermore, these immune sentinels preserve vascular homeostasis through dual mechanisms: production of immunomodulatory cytokines (e.g., IL‐10) and regulation of ECM composition and dynamics, effectively attenuating inflammatory cascades. A recent research by Zenget al. found that macrophages in skin wounds in a diabetic state polarize to M1 type, targeting vascular endothelial cell HELZ2 protein by secreting extracellular vesicles carrying miR‐ERIA, thereby inhibiting vascular endothelial cell migration and tube‐forming ability and ultimately leading to delayed wound healing [108]. Furthermore, macrophages have been shown to maintain vascular endothelial cell stability by secreting antiinflammatory cytokines (e.g., IL‐10) and regulating ECM components to reduce inflammatory responses.

### 4.3 Immune Regulation

Immunoregulation is defined as the complicated interactions between immune molecules, immune cells, and the immune system with other bodily systems during immune responses. These interactions establish a regulatory network that coordinates and constrains various processes, thereby ensuring that the immune response is appropriately calibrated in terms of strength and quality to maintain the stability of the body's internal environment. Typically, systematic analysis of immunometabolic pathways focuses on macrophages, which are central to both pro‐ and anti‐inflammatory immune responses [109]. It is now widely acknowledged that these cells play a pivotal role in immune regulation, a process that is critical for maintaining health. A mounting body of evidence suggests a critical functional linkage between the bioenergetic reprogramming of polarized macrophages (M1/M2 phenotypes) and their immunomodulatory capacities. Systematic elucidation of the dynamic interactions governing metabolic reprogramming dynamics and PRR cascades within these immune cells may establish a conceptual foundation for designing targeted intervention strategies against chronic inflammatory pathologies [110].

IFN‐γ and LPS have been shown to trigger macrophages, leading to the TCA cycle displayed through integrated transcriptional and metabolic pathways. Inhibition of succinate dehydrogenase by the citric acid metabolite succinate in the TCA cycle is a key feature of IFN‐γ/LPS‐polarized macrophages. Emerging pharmacological studies have demonstrated that succinate, acting as an immune‐regulating metabolite, possesses dual therapeutic properties, which include both significant attenuation of inflammatory cascades and effective suppression of pathogenic microorganisms. It is noteworthy that alterations in metabolite concentrations have the capacity to directly modify the function of signaling pathways. HIF‐1α stabilization, induced by LPS‐induced succinate accumulation in macrophages, has been shown to promote proinflammatory cytokine IL‐1β expression [111]. The proteostatic maintenance of HIF‐1α drives glycolytic reprogramming in proinflammatory macrophages. This oxygen‐sensitive transcription factor orchestrates the transcriptional activation of essential enzymatic components involved in carbohydrate metabolism, such as the lactate export machinery (MCT4/SLC16A3) and hexose uptake systems (GLUT1/SLC2A1). M1 macrophages have also been shown to exhibit an enhanced pentose phosphate pathway, which produces NADPH [112]. It has been established that NADPH plays a crucial role as a cofactor for LPS‐induced iNOS, facilitating the catabolism of arginine into NO and l‐citrulline. It is noteworthy that NADPH not only generates NO and ROS, but also contributes to the production of the antioxidant glutathione, which is vital for maintaining redox homeostasis and averting cellular damage from ROS [113]. Additionally, NO serves as a central regulatory mediator that governs metabolic reprogramming in classically activated macrophages. Through dual molecular mechanisms involving the nitrosylation of the [4Fe–4S] enzymatic complex and the consequent irreversible disruption of mitochondrial electron transfer, this gaseous signaling molecule fundamentally compromises mitochondrial bioenergetic efficiency. It effectively uncouples the progression of the TCA cycle from ATP synthase activity, while imposing energetic constraints on oxidative metabolism [114].

Distinct bioenergetic configurations characterize macrophage polarization states, with M2 variants exhibiting significant divergence from their proinflammatory M1 counterparts. This dichotomy reflects their specialized roles in immunoregulation and stromal maintenance. Central to this metabolic compartmentalization is the differential engagement of ATP synthesis pathways: M1 polarization preferentially drives flux through the glycolytic pathway, often accompanied by the accumulation of itaconate, whereas M2 activation coordinates mitochondrial oxidative metabolism through the TCA cycle, coupled with mitochondrial OXPHOS, thus optimizing the electron transport chain for energy production. This process is facilitated by the glutamate metabolic pathway, which involves β‐fatty acid oxidation and α‐ketoglutarate. The IL‐4/IL‐13 signaling axis orchestrates metabolic reprogramming in macrophages by transcriptionally enhancing fatty acid β‐oxidation and mitochondrial biogenesis. This process is mechanistically dependent on the integrated activation of STAT6, members of the PPAR nuclear receptor family, and PGC‐1β. M1 and M2 macrophages exhibit opposing arginine metabolism; M1 macrophages upregulate iNOS to metabolize l‐arginine into the antimicrobial substances NO and l‐citrulline, while M2 macrophages catalyze l‐arginine to urea and l‐ornithine through the induction of Arg1 [117, 118].

The M1/M2 polarization state of macrophages has been demonstrated to exhibit bidirectional roles in immunomodulation. It has been shown that M1‐type macrophages activate antitumor immunity by secreting proinflammatory factors such as TNF‐α and IL‐12, whereas M2‐type macrophages promote immunosuppression and tissue repair through mediators such as IL‐10 and TGF‐β [119]. Notably, 3,3′,5‐triiodothyronine (T3) has been found to have a dual regulatory role, promoting M1 polarization to enhance inflammatory response and inhibiting M2 activity to maintain immune homeostasis, illustrating a dynamic regulatory mechanism with implications for autoimmune disease and tumor therapy [120]. M2‐type polarization of TAMs is a fundamental factor in tumor immune escape, which promotes angiogenesis and suppresses T cell function by secreting molecules such as VEGF and PD‐L1. Recent studies have developed pH‐responsive nanoparticles, which can reprogram TAM to the M1 phenotype by precisely modulating lysosomal function, significantly enhancing antigen presentation efficiency and activating CD8^+^ T cells [121]. In addition, a combination of GM‐CSF secretion mediated by engineered bacteria and SIRPα–siRNA delivery has been shown to synergistically block the CD47–SIRPα immune checkpoint pathway, thereby significantly enhancing the antitumor efficacy [122].

### 4.4 Neuronal Network Protection

The neuronal network is comprised of highly specialized neurons and their synaptic connections, which achieve information integration and regulation through electrochemical signaling. It is a central carrier of nervous system function. Macrophages (e.g., microglia, which are resident macrophages in the central nervous system [CNS]) can regulate the microenvironment of neural stem cells. These cells play a critical role in safeguarding neuronal networks across multiple levels, including the regulation of the inflammatory microenvironment, the maintenance of neurohomeostasis, the execution of phagocytic clearance and damage repair, and the regulation of metabolism and energy homeostasis. M2‐type macrophages, in particular, have been shown to inhibit excessive inflammatory responses and reduce neuronal apoptosis by secreting antiinflammatory factors such as IL‐10 and TGF‐β.

In a spinal cord injury model, TREM2 knockout macrophages significantly improved neuronal survival by decreasing levels of proinflammatory factors TNF‐α and IL‐1β [123]. Macrophages also promote neuronal survival and axonal regeneration by secreting BDNF) and NT‐3 [124]. Following spinal cord injury, an increase in the number of dorsal root ganglion macrophages (DRGMacs) is observed, primarily through self‐renewal. A subset of DRGMacs undergoes a transformation into a microglial‐like state following nerve injury, with these cells potentially migrating from the spinal cord to DRG and contributing to neuroprotection and repair. A further subset of DRGMacs displays characteristics analogous to satellite glial cells, which have been observed to express macrophage‐associated genes post‐nerve injury and potentially contribute to immune responses and neuroprotection [125]. Research has demonstrated that fumarate hydratase (FH) modulates the proinflammatory and reparative functions of macrophages by regulating mitochondrial RNA release and interferon‐β signaling pathways. Inhibition of FH enhances the antiinflammatory phenotype of macrophages and attenuates neuronal damage in neurodegenerative lesions [126, 127]. Furthermore, the study identified that MDE carry noncoding RNA species, such as miR‐155 and miR‐21, which traverse the neurovascular unit, achieving cerebral parenchymal infiltration through selective modulation of tight junction complexes and endothelial transcytosis pathways, thereby modulating synaptic plasticity and neuronal electrical activity [128].

Accumulating experimental evidence delineates the pathophysiological contributions of myeloid phagocytes, particularly circulating monocytes, TRMs, and microglial cells, in mediating neuroimmune interactions that drive inflammation‐associated axonal degeneration, CNS structural compromise, and resultant neurobehavioral alterations in mammalian models [129, 130]. Notably, cerebral phagocytic populations constitutively express PRRs, including TLR4 and NLRP3 inflammasomes, which mechanistically enable their capacity to mediate structural plasticity through dendritic spine pruning and synaptic remodeling [131, 132]. The functional polarization of these immunoregulatory cells is not merely determined by passive cytokine exposure; rather, it is shaped by the dynamic equilibrium between neurodestructive mediators (e.g., IL‐1β/TNF‐α) and neurotrophic factors (e.g., glial cell‐derived neurotrophic factor [GDNF]/insulin‐like growth factor 1), which collectively dictate their dichotomous roles in neural circuit disruption versus restoration. Furthermore, the adrenergic signaling–Arg1–polyamine metabolic axis in muscular macrophages mediates neuroprotective adaptations during infectious challenges, where activation of the β2‐adrenergic receptor triggers pharmacological induction of Arg1 enzymatic activity, thereby elevating polyamine biosynthesis to prevent infection‐associated neurodegeneration [133].

During the process of tissue healing following acute injury, macrophages are responsible for the removal of cellular debris from the affected area through a process known as thrombospondin‐1 (TSP‐1)‐dependent phagocytosis. In addition to this primary function, these cells also release metabolites, such as adenosine, which play a crucial role in promoting angiogenesis and axon regeneration. The activation of calcitonin gene‐related peptide (CGRP) neurons has been observed to enhance the phagocytic activity of macrophages. It has been demonstrated that CGRP neurons are responsible for accelerating the repair of skin and muscle injuries. The neuropeptide CGRP exhibits dual immunomodulatory functions through coordinated molecular mechanisms. It mediates neutrophil efferocytosis via Rho GTPase‐dependent cytoskeletal reorganization and drives macrophage phenotypic switching toward an immunoregulatory (M2‐like) state through TSP‐1‐mediated autocrine/paracrine signaling loops [124].

Macrophages, a fundamental component of organismal homeostasis, function as a regulatory axis that orchestrates a multifaceted physiological barrier through phagocytosis. This process encompasses the elimination of metabolic byproducts, the orchestration of tissue repair microenvironments, the maintenance of immune dynamic homeostasis, and the engagement in neural synaptic pruning. However, their high degree of plasticity endows macrophages with the capacity to dynamically transform in pathological microenvironments. In such microenvironments, macrophages can either initiate a protective program by sensing injury signals through PRRs or be driven by abnormal microenvironments to develop proinflammatory or profibrotic phenotypes. These phenotypes ultimately influence the direction of disease progression.

## 5 Macrophages in Diseases

### 5.1 Autoimmune Diseases

Macrophages play a crucial role in the progression of various autoimmune disorders due to their diverse functional repertoire, which includes immunoregulation, proinflammatory activation, and stromal remodeling. These myeloid sentinels orchestrate complex chemokine networks through the dynamic secretion of soluble mediators that recruit and prime adaptive immune cell populations, thereby establishing self‐perpetuating inflammatory circuits via paracrine signaling mechanisms.

**FIGURE 4:**
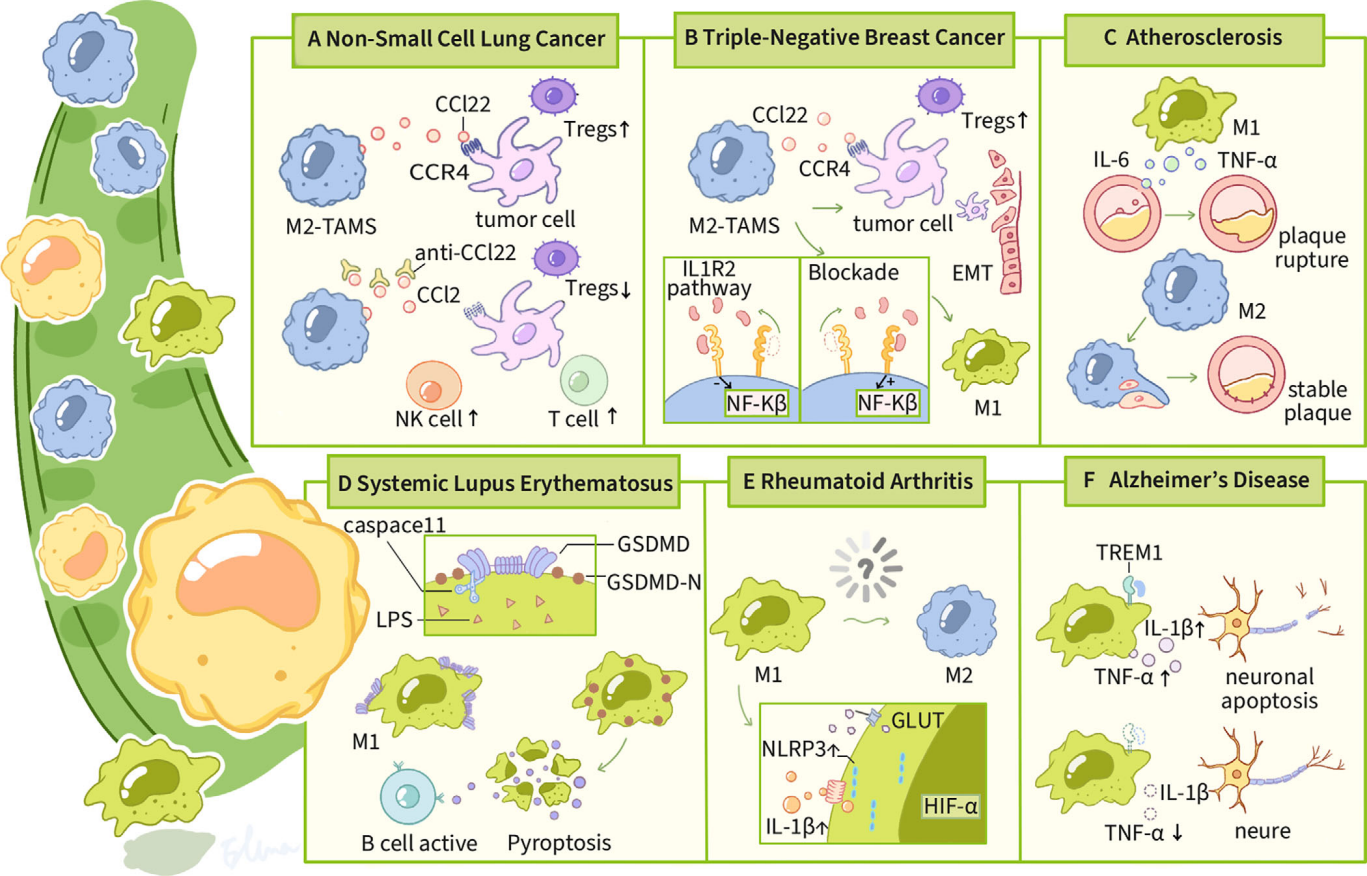
The functionality of macrophages in neoplastic microenvironments. This paper explores the molecular pathways through which immunocompetent cells interact with neoplastic microenvironments across various clinical conditions. The diagram systematically outlines the functional contributions of distinct immune cell populations, specifically M1 and M2 macrophages, lymphocyte subsets (T and B cells), and cytotoxic NK cells, within multiple disease contexts, including NSCLC, TNBC, atherosclerosis, SLE, RA, and AD. Key signaling pathways and molecules, such as CCL22, CCR4, IL‐1R2, NF‐κB, NLRP3, and HIF‐α, are labeled alongside their roles in immune regulation, inflammatory response, and apoptosis.

SLE is an autoimmune disease characterized by an overactive immune system, the presence of autoantibodies, and multisystem damage, resulting in a wide heterogeneity of clinical manifestations  .The etiology of SLE is complicated and involves both genetic susceptibility and environmental factors [134, 135]. One hypothesis is that residual cellular debris generated by increased cell death initiates immune overactivity and induces immunologic loss [136]. Research efforts have increasingly concentrated on the immunomodulatory effects of regulated cell death modalities, particularly their ability to undermine immunological tolerance and dynamically reconfigure adaptive immune circuits. The study of the role of innate immune cells in the pathogenesis of SLE has also attracted considerable interest, as there is a disruption in the homeostasis between macrophage subtypes in SLE, which is more pronounced in the affected organs. LPS leakage from damaged intestinal barriers was found to induce GSDMD‐mediated cellular pyroptosis through the TLR4/caspase 11 pathway in MRL/lpr mice. Moreover, pyroptotic macrophages promoted the differentiation of naive B cells into plasmoblasts and plasma cells, which may exacerbate the pathogenesis of lupus. Additionally, inhibition of caspase 11 and intestinal barrier repair with antibiotics effectively inhibited the caspase 11/GSDMD pathway and attenuated the manifestation of lupus in mice [137] (Figure 4) .

RA, a prevalent immune‐mediated disorder, is characterized by a complex disease progression involving inflammatory cascades that target the synovium and lead to destructive polyarticular involvement. This often results in irreversible joint degradation and significant functional impairment [138]. Pathological analyses indicate a marked dysregulation in macrophage polarization dynamics, with a shift in the balance from proinflammatory (M1) to antiinflammatory (M2) phenotypes, favoring dominant M1 activation throughout the pathogenesis of RA [139, 140]. Macrophages are critical in the initiation and maintenance of synovitis in RA, where they can act as antigen‐presenting cells leading to T‐cell‐dependent B‐cell activation and production of damaging cytokines, but macrophages are also involved in maintaining tissue homeostasis/repair [141]. Pathological and physiological macrophages differ in phenotype and function (e.g., cytokine secretion) and exhibit polymorphism. Reprogramming M1 macrophages to M2 using targeted IL‐10 gene therapy prevents joint inflammation and damage associated with arthritis [142]. Rosmarinic acid, a polyphenolic derivative derived from Prunus serotina, orchestrates immunometabolic reprogramming through the dual‐axis suppression of the ERK/hypoxia‐inducible factor 1 alpha (HIF‐1α)/GLUT1 signaling cascade. This targeted kinase modulation attenuates Warburg‐like glycolysis, facilitating a phenotypic transition from classically activated macrophages to alternatively polarized phenotypes. This metabolic switch not only ameliorates synovitis‐mediated joint destruction but also enhances chondroprotective repair mechanisms in RA models [143]. Thus, modulation of joint‐associated macrophage subtypes has significant therapeutic potential.

Synovial tissue is the primary site of joint inflammation in RA patients. Chronic synovitis results in irreversible damage to cartilage and bone. TAMs are evolutionarily conserved innate immune effectors that originate from fetal liver‐derived precursors or bone marrow myeloid progenitors. These sentinel cells perform organ‐specific homeostatic functions, ranging from the efferocytic clearance of apoptotic debris to the sequestration of pathogens, as exemplified by the alveolar surveillance mechanisms that maintain sterility during respiratory gas exchange. Crucially, macrophages act as immunoregulatory rheostats, coordinating a biphasic inflammatory cascade that initiates neutrophil recruitment and subsequently resolves sterile injury. During parenchymal damage, local macrophage pools are augmented by circulating monocyte‐derived counterparts, establishing heterotypic cellular crosstalk networks that calibrate the intensity of inflammation [144]. The dynamic equilibrium between CD169+ synovial‐resident macrophages and CCR2^+^ monocyte‐derived infiltrating macrophages dictates the temporal progression of synovitis and the fidelity of articular regeneration processes in rheumatoid joints [145]. Landmark clinical observations, particularly the pathognomonic association of STM (CD68^+^CD163^−^) density gradients with radiographic joint space narrowing [146], coupled with spatial transcriptomic evidence demonstrating the colocalization of macrophage activation markers (CD86/MHC‐II) with subchondral bone erosion foci in high‐DAS28 joints [147], have provided mechanistic insights into macrophage‐mediated synoviocyte hyperplasia and osteoclast activation during the progression of RA.

### 5.2 Neurodegenerative Diseases

As the predominant neurodegenerative dementia subtype, Alzheimer's disease (AD) clinically manifests as relentless mnestic dysfunction and multidomain cognitive deterioration. Advancing chronological age emerges as the primary nonmodifiable determinant in AD pathogenesis, and both are associated with chronic inflammation (sometimes referred to as “inflammatory aging”) and an impaired immune response. Microglia are the major resident macrophages in the brain, accounting for 10% of all glial cells. In the early stages of AD, they play a protective role by phagocytosing β‐amyloid (Aβ) and tau proteins, but as the disease progresses, prolonged activation releases proinflammatory factors (e.g., IL‐1β, TNF‐α) that exacerbate neuroinflammation [148, 149, 150]. In recent years, it has been found that macrophages of peripheral origin can enter the AD brain through the blood–brain barrier and synergize with central microglia to regulate pathological processes. For example, CX3CR1^+^ BAMs are enriched around Aβ plaques, but their clearance is affected by lipid metabolic status [150, 151, 152]. Microglia rely on the TREM2 receptor to recognize and phagocytose Aβ, while secreting insulin‐degrading enzyme to directly degrade Aβ. Knockdown of the myeloid triggering receptor TREM1 protects glucose metabolism in peripheral macrophages and oxidative phosphorylation in brain neurons. Restoration of immune responses that support healthy brain function [153]. In addition, a study shows that MDM activation is independent of TREM2 and that blocking monocyte migration with anti‐Ccr2 antibody completely abolishes the cognitive‐improving effects of anti‐PD‐L1 treatment in Trem2^−/−^5XFAD mice [154]. On the other hand, high‐fat diet and APOE4 genotype exacerbate lipid droplet deposition in microglia, leading to decreased phagocytosis through inhibition of the PI3K/AKT pathway. Knockdown of Fit2, a key gene for lipid droplet formation, was shown to increase Aβ clearance by 40% [150]. Activated microglia are converted to a glycolytic phenotype, leading to lactate accumulation and disruption of neuronal mitochondrial function [155, 156].

Macrophages play a dynamic, double‐edged role in AD and their function is coregulated by genetic, metabolic and microenvironmental factors. Future studies need to combine single‐cell multiomics, organoid models, and clinical cohorts to analyze the spatio‐temporal specific functions of different subpopulations of macrophages and to develop stage‐appropriate therapeutic strategies.

### 5.3 Cancers

TAMs constitute critical stromal constituents of the neoplastic niche, exerting pleiotropic regulatory effects on malignant proliferation, VEGF‐mediated neovascularization, and PD‐L1‐dependent immune checkpoint activation [157, 158, 159]. Ontogenetically stratified, these myeloid populations segregate into embryonically derived tissue‐resident and hematopoietic progenitor‐derived monocytic lineages. Functionally, these myeloid infiltrates pervasively colonize solid neoplasms, driving oncogenic processes, angiogenic switching, metastatic dissemination, and desmoplastic stromal barrier formation, ultimately facilitating immune‐privileged tumor evolution.

Triple‐negative breast carcinoma (TNBC), defined by the immunohistochemical absence of estrogen receptor, progesterone receptor, and HER2/neu expression, represents the most therapeutically challenging breast cancer subtype with aggressive metastatic potential. While bidirectional tumor–stroma signaling has been extensively characterized in mammary malignancies [160], the precise molecular circuitry through which TNBC cells reprogram TAMs remains enigmatic. Emerging data delineate a feedforward TNBC–TAM communication IL‐6/TGF‐β1‐mediated paracrine signaling induces constitutive HLF activation in neoplastic cells. Mechanistically, HLF orchestrates ferroptosis resistance via transcriptional upregulation of GGT1, thereby enabling glutathione‐mediated redox homeostasis and fueling TNBC malignant progression. These findings unveil the HLF/GGT1 regulatory node as a promising therapeutic vulnerability in TNBC pathobiology [161]. A monocyte‐derived subpopulation of STAB1^+^TREM2^high^ lipid‐associated macrophages (LAM) expanded in patients resistant to immune checkpoint blockade (ICB) therapy; this LAM subpopulation is immunosuppressive and acquires tumor‐promoting capacity upon recruitment to the tumor site via the cancer‐associated fibroblast (CAF)‐driven CXCL12–CXCR4 axis, which supports an immunosuppressive microenvironment [162]. IL‐1 receptor type 2 (IL1R2) orchestrates breast tumor‐initiating cell (BTIC) stemness maintenance and neoplastic expansion in TNBC. Mechanistically, preclinical TNBC models revealed that pharmacological IL1R2 antagonism potently suppressed CCL2‐mediated myeloid cell infiltration and M2‐like TAM polarization, concomitantly impairing BTIC clonogenicity while mitigating CD8^+^ T‐lymphocyte dysfunction. This multimodal immunomodulation consequently attenuated oncogenic progression and conferred significant overall survival extension in murine TNBC systems through STAT3 signaling axis inactivation and glutathione peroxidase 4‐mediated ferroptosis sensitization. This suggests that targeting this molecule may improve patient treatment [163]. In addition, TAM secretes IL‐1β and TNF‐α, which activate the IKK/NF‐κB pathway in TNBC cells via IL1R/TNFR, inducing increased autophagy and promoting cell migration and invasion. Animal models showed that targeted inhibition of this pathway significantly reduced lung metastasis [164]. In conclusion, TAM plays a central role in TNBC progression through polarization regulation, metabolic interaction and immune escape mechanisms.

Non‐small cell lung cancer (NSCLC) is the most prevalent form of lung cancer, constituting 80–85% of all lung cancers. It encompasses primarily squamous cell carcinoma, adenocarcinoma, and large cell carcinoma. TAMs in the interstitium of NSCLC are predominantly of the M2 phenotype (CD68^+^TGF‐β1^+^), which is significantly higher than that in paracancerous tissues, and the M2 phenotype inhibits antitumor immunity by secreting factors such as IL‐10 and TGF‐β, promoting angiogenesis and stromal remodeling [165]. The infiltration level and spatial distribution of TAMs were found to have a significant impact on the outcome of NSCLC patients treated with ICB [166]. It is also significant that the degree of TAM infiltration in NSCLC was associated with upregulation of CD27, ITGAM, and CCL5 gene expression. The protein products of these genes play a key role in controlling tumor macrophage polarization and may be novel immunotherapeutic targets [165]. An in vitro coculture model study showed that NSCLC cells are capable of inducing macrophages to express an immunosuppressive M2‐type phenotype, particularly high expression of Arg1. This model provides an effective tool to study the biological functions of macrophages in NSCLC and to develop therapeutic strategies against macrophages.

Esophageal squamous cell carcinoma (ESCC) is a malignant tumor derived from esophageal squamous epithelial cells and is the major pathological type of esophageal cancer. TAMs potentiate neoplastic invasion through paracrine secretion of protumorigenic factors while simultaneously driving VEGF‐A‐driven neovascularization. TAM abundance exhibits a linear correlation with CD31^+^ microvessel density indices, suggesting their dual role in stromal remodeling and angiogenic switch regulation during ESCC progression [167]. The M2‐polarized phenotype of TAMs is an important feature of the ESCC microenvironment, and M2‐type macrophages promote tumor immune escape and angiogenesis by secreting factors such as VEGFA and IL‐10 [168]. And, CAFs recruit and induce tumor cell invasion and angiogenesis by secreting PAI‐1, CCL2, and other factors to recruit and induce macrophage M2 polarization while enhancing migration and invasion of ESCC cells [169]. Therapeutic strategies that target the Hippo/YAP–CD24/Siglec‐15 signaling nexus exhibit bimodal therapeutic efficacy in ESCC by suppressing Hippo/YAP‐driven oncogenic signaling cascades and enhancing macrophage‐mediated efferocytosis through the blockade of the STAT6‐mediated “don't eat me” signal (CD47/SIRPα axis). This mechanism holds significant translational potential in the context of precision oncology for ESCC [170].

### 5.4 Liver Diseases

#### 5.4.1 Viral Hepatitis

Viral hepatitis is a liver disease caused by hepatitis viruses and is classified as an infectious disease. The primary clinical manifestations include decreased appetite, nausea, upper abdominal discomfort, liver pain, and fatigue. Some patients may also experience jaundice, fever, and liver enlargement, often accompanied by impaired liver function. A subset of patients may develop chronic hepatitis, which can lead to cirrhosis, and in a few cases, progress to liver cancer. Diagnosis of viral hepatitis primarily relies on identifying the etiology, which encompasses five types: A, B, C, D, and E. We will focus specifically on hepatitis B and C viruses [171].

Despite their distinct virological profiles, hepatitis B virus (HBV) and hepatitis C virus (HCV) share immunopathogenic mechanisms, where the balance between hepatic viral eradication and persistence is governed by the dynamic equilibrium between innate PRRs and adaptive lymphocyte‐mediated immune surveillance [172]. KCs, the liver's specialized macrophages strategically located in sinusoidal endothelial fenestrae, function as immunological gatekeepers. They paradoxically maintain defense against hepatotropic pathogens while also mediating inflammatory cascades that promote fibrosis [172].

Both HBV and HCV are primarily transmitted through percutaneous and sexual routes, with perinatal transmission occurring predominantly in the case of HBV [173, 174, 175]. Infections caused by these viruses may either resolve spontaneously or progress to chronic liver disease characterized by ongoing viral replication within hepatocytes [176]. Persistent hepatic inflammation serves as a critical pathophysiological driver of hepatic fibrogenesis, the progression of cirrhosis, and ultimately hepatocarcinogenesis [177]. Immunocompetent hosts who achieve spontaneous viral resolution exhibit a broad‐spectrum adaptive immunity characterized by polyfunctional CD4^+^ T helper (Th1/Tfh) cells, cytotoxic CD8^+^ T effector memory re‐expressing CD45RA (TEMRA) cells, and neutralizing antibody‐producing B cell clones that target conserved viral epitopes. In contrast, chronic HBV/HCV carriers demonstrate attenuated adaptive immunity [178]. This observation underscores the importance of strong, multiepitope‐specific T and B cell responses in the clearance of infection, which can only be achieved following effective innate immune responses [179].

HBV‐infected hepatocytes release viral progeny particles, HBsAg, and the nonstructural secretory variant HBeAg into systemic circulation, with these viral components being routinely quantified in patient serum [174]. Nevertheless, conclusive evidence of productive HBV replication in nonhepatocytic cell populations has yet to be definitively established. Crucially, the potential intracellular retention of HBV antigens within KCs under physiological conditions, along with the capacity of human KCs to internalize intact virions or viral subcomponents ex vivo, remain uncharacterized. In contrast, in vitro models employing THP‐1 monocytes, PBMCs, and dendritic cells have demonstrated HBV ligand–receptor interactions that trigger downstream immunostimulatory cascades. Mechanistic studies reveal TLR2‐mediated recognition of HBcAg–hsp70 complexes and heparan sulfate proteoglycan (HSPG)‐dependent viral attachment in THP‐1 cells, culminating in NF‐κB‐dependent cytokine production (IL‐6, IL‐12, TNF‐α) and inflammasome priming through ASC speck formation [180]. However, since HBcAg is exclusively found within infected hepatocytes or viral particles, the potential interaction of HBcAg with KCs via HSPG or other extracellular receptors such as TLR2 remains uncertain. Multiple receptor–ligand interactions between KC surface molecules and HBV components across experimental systems. Notably, HBsAg demonstrates CD14‐dependent binding to peripheral monocytes and mannose receptor‐mediated engagement with dendritic cells [174]. Furthermore, HBsAg–albumin complexes may enhance scavenger receptor‐mediated endocytosis of viral antigens by sinusoidal endothelial cells and KCs through opsonization mechanisms [173, 174]. Within the hepatic immunological niche, dendritic cells and MoMFs coordinate adaptive immunity via antigen cross‐presentation while secreting cytokines that modulate HBV cccDNA transcriptional activity [181]. Crucially, M1‐polarized macrophages produce IL‐1β and IL‐6 that suppress HBV replication through JAK/STAT1 pathway activation and proteasomal degradation of viral core particles.

The HCV contains a 9.6 kilobase positive‐sense RNA genome that translates into a polyprotein precursor subsequently cleaved into structural components (core, E1/E2 glycoproteins) and nonstructural regulators (NS1–NS5). Postreplicative assembly yields 55–65 nm enveloped virions packaging genomic RNA within nucleocapsid complexes [171]. In contrast to HBV, HCV employs a multistep entry pathway involving hepatocyte surface molecules beyond claudin‐1/occludin tight junctions—including EGFR, EphA2, HSPG, LDL‐R, SR‐B1, and tetraspanin CD81. Notably, only a subset of these receptors (e.g., CD81, SR‐B1) are functionally expressed on KCs. Experimental evidence indicates HCV–E2 glycoprotein engages KCs through CD81‐mediated interactions [171], while DC‐SIGN—a hepatocyte‐absent C‐type lectin receptor—mediates viral attachment to KC surface glycans through high‐mannose N‐linked glycosylation sites on HCV envelope proteins. [176, 177]. Although it is unlikely that HCV can replicate within KC, activation of these cells by HCV and its proteins has been documented. Specifically, HCV core and NS3 proteins stimulate CD14^+^ KCs and MoMFs, derived from human liver perfusate, via TLR2, resulting in the production of proinflammatory cytokines such as IL‐1β, IL‐6, and TNF, as well as the immunosuppressive cytokine IL‐10 [182, 183]. Contemporary studies have elucidated that TLR4 mediates NS3 detection in purified liver‐derived KCs through differential centrifugation and adherence‐based isolation protocols, triggering MyD88‐dependent TNF‐α secretion [182]. Nevertheless, the limited secretory capacity of HCV core and NS3 proteins from infected hepatocytes curtails their extracellular availability for KC pattern recognition via canonical TLR pathways. A plausible alternative hypothesis posits that phagocytic clearance of HCV‐infected hepatocytes by KCs could facilitate intracellular exposure to viral RNA‐derived PAMPs within endosomal compartments.

#### 5.4.2 Alcohol‐Associated Liver Disease

**FIGURE 5:**
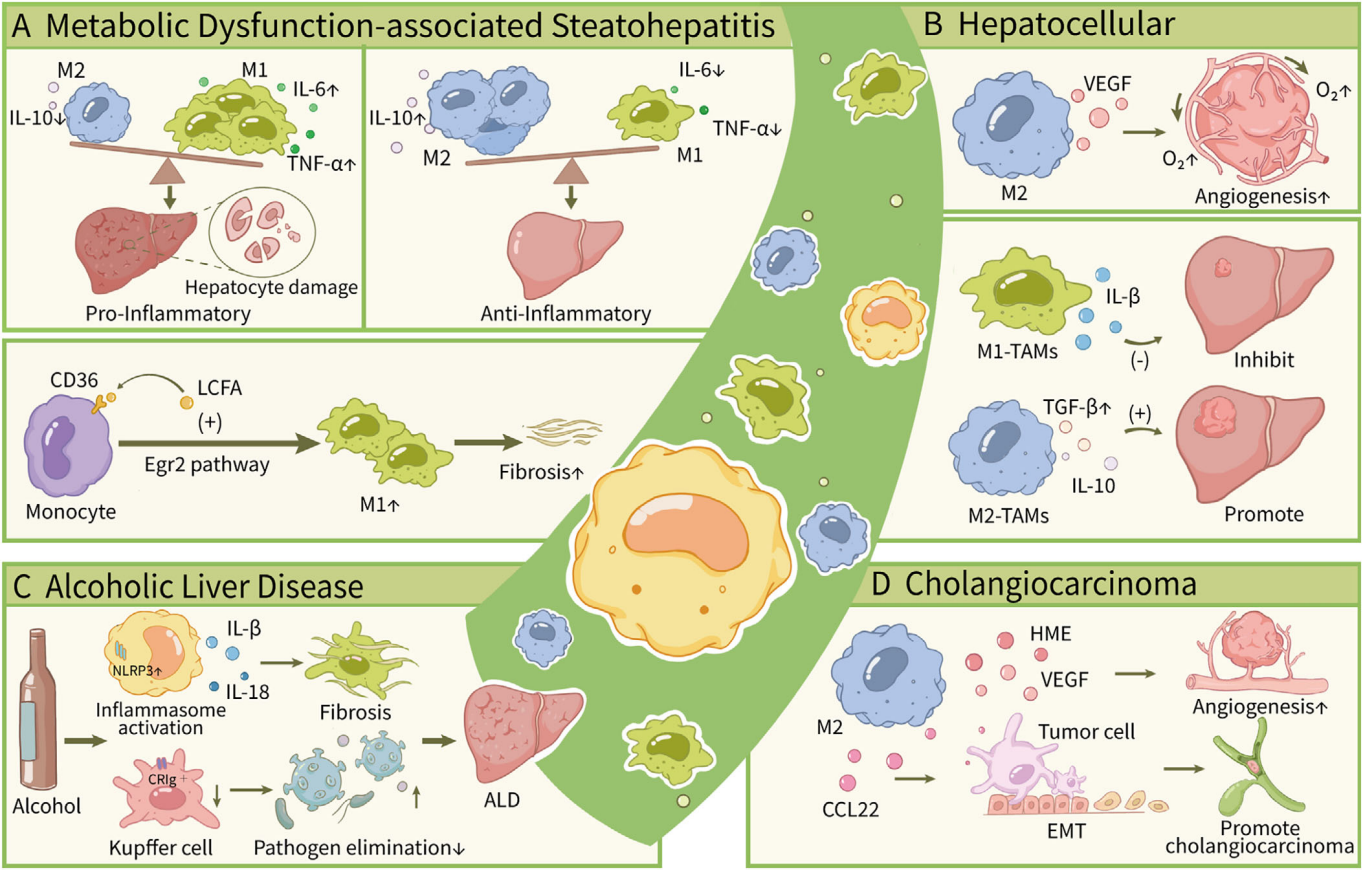
Diverse roles of immune cells in liver diseases and their pathological mechanisms. The interactions of immune cells in various liver diseases, including (A) metabolic dysfunction‐associated steatohepatitis (MASH), (B) hepatocellular carcinoma (HCC), (C) alcoholic liver disease (ALD), and (D) cholangiocarcinoma (CCA), and their impact on disease progression, highlighting the functional transitions of M1 and M2 macrophage types across different pathological conditions and their effects on hepatocyte injury, fibrosis, inflammatory responses, and tumor angiogenesis. These processes are mediated by the secretion of cytokines such as IL‐6, IL‐10, and TNF‐α. Furthermore, the role of alcohol intake in promoting hepatic fibrosis through the activation of inflammatory vesicles is discussed, alongside the mechanisms by which tumor cells in cholangiocarcinoma facilitate angiogenesis and tumor progression via EMT.

Alcohol‐associated liver disease (ALD) represents a preeminent global health burden, accounting for the majority of chronic hepatopathies worldwide [184, 185, 186, 187] and constituting the principal indication for hepatic transplantation in Western healthcare systems [188]. Within its clinical spectrum, alcoholic hepatitis emerges as a critical syndrome of acute‐on‐chronic hepatic decompensation, exhibiting a 28‐day mortality risk exceeding 30% and 90‐day mortality rates reaching 50% in severe presentations. Orthotopic liver transplantation persists as the sole disease‐modifying intervention for eligible candidates meeting stringent abstinence criteria, though its application remains constrained by donor organ scarcity and complex ethical considerations [189, 190, 191]. The pathophysiological continuum of ALD is inextricably linked to ethanol‐induced enteric dysbiosis, characterized by Bacteroidetes depletion and Proteobacteria expansion [192, 193, 194]. These microbial community perturbations potentiate gut barrier dysfunction, driving endotoxin translocation and hepatic inflammasome activation [195, 196, 197]. The dysbiosis of intestinal flora, in turn, induces intestinal barrier dysfunction and permits the transfer of live bacteria from the gut to the liver [198, 199]. Alcohol‐associated liver injury has been shown to induce changes in the composition and phenotype of hepatic macrophages and a decrease in the number of CRIg^+^ KC. These changes have been demonstrated to impair the clearance of pathogenic bacteria. The detrimental effects of ethanol‐induced CRIg suppression in KCs were effectively neutralized through soluble CRIg‐Ig supplementation, demonstrating hepatoprotective benefits in murine models of alcohol‐related hepatic injury. Alcohol has been shown to reduce CRIg expression in the liver by altering the composition and phenotype of hepatic macrophages, thereby compromising the hepatic firewall [200]. This compromised CRIg expression directly impairs hepatic clearance mechanisms for gut‐derived pathogens, establishing a pathophysiological link to aggravated hepatic pathologies ranging from terminal hepatic conditions (such as cirrhotic degeneration) to elevated risks of disseminated infections in alcohol‐associated hepatitis. These cascading effects position CRIg modulation as a novel therapeutic frontier in managing ethanol‐related hepatic disorders [201, 202] (Figure 5).

#### 5.4.3 Metabolic Dysfunction‐Associated Steatohepatitis

Formerly classified as NAFLD, the newly defined MASLD diagnostic entity describes hepatic disorders characterized by aberrant intracellular lipid accumulation [203]. The disease continuum progresses from benign steatotic manifestations through inflammatory MASH phases toward irreversible fibrotic damage [204]. Despite established correlations between fibrotic burden and clinical outcomes in MASLD [205], the fundamental mechanisms mediating disease advancement persist as unresolved scientific questions, creating critical barriers to developing effective antifibrotic MASH interventions [206]. In light of these observations, macrophages have been shown to exhibit both proinflammatory and antiinflammatory functions in the context of MASH [207, 208]. Specifically, m1‐type macrophages have been observed to secrete cytokines such as TNF‐α and IL‐6, which have been implicated in the induction of hepatocellular injury and fibrosis. Conversely, M2‐polarized macrophages demonstrate antiinflammatory potential via IL‐10 secretion, concurrently facilitating tissue regeneration through cytokine‐mediated pathways. Within hepatic fibrogenesis, hepatic stellate cells (HSC) and their transdifferentiated myofibroblast derivatives serve as central mediators. Contemporary research reveals that macrophage‐mediated regulation of HSC activation states constitutes a critical immunofibrogenic axis driving ECM deposition [209].

A defining pathological hallmark of MASH involves extensive infiltration of circulatory monocytes that undergo terminal differentiation into macrophage populations, ultimately displacing embryonically‐derived KCs [210]. These infiltrating myeloid cells, classified as scar‐associated macrophages (SAMs) [211, 212], exhibit phenotypic parallels with lipid‐encapsulating macrophages (LAMs) observed encircling necrotic adipocytes in obese adipose depots, warranting their designation as hepatic LAMs [212]. Mechanistic studies confirm SAMs mediate cellular crosstalk with stromal constituents, directly propelling MASH‐to‐fibrosis transition across species [212, 213]. Crucially, the zinc‐finger transcription factor Egr2 governs monocyte‐to‐hLAM differentiation in MASH microenvironments. Pathological accumulation of saturated long‐chain fatty acids (LCFAs) activates macrophage Egr2 signaling, a metabolic reprogramming process suppressed by unsaturated LCFA counterparts. Pharmacological modulation of this monocyte‐specific saturated LCFA–Egr2 pathway consequently represents a rational therapeutic strategy for MASH intervention [214].

XBP1 expression was found to be significantly increased in liver samples from MASH patients. In a mouse model, the deletion of hepatocyte‐specific Xbp1 in subjects fed a high‐fat or methionine/choline‐deficient diet resulted in a substantial inhibition of steatohepatitis development [215]. Furthermore, the study observed that macrophage‐specific Xbp1 knockout mice exhibited reduced sensitivity to high‐fat and methionine/choline‐deficient diets in comparison with wild‐type Xbp1^FL/FL^ mice. Notably, the study observed an anti‐inflammatory M2 polarization in the macrophages of Xbp1‐deficient mice. The severity of steatohepatitis was found to be reduced in Xbp1‐deficient macrophages through decreased NLRP3 expression and proinflammatory cytokine secretion [215]. Furthermore, the severity of steatohepatitis was also reduced in Xbp1‐deficient Nlrp3 knockout mice compared with wild‐type Nlrp3^FL/FL^ mice. Furthermore, the study observed that xbp1‐deficient macrophages inhibited hepatic stellate cell activation by decreasing TGF‐β1 expression, leading to a reduced incidence of fibrotic changes in macrophage‐specific Xbp1 knockout mice compared with wild‐type Xbp1^FL/FL^ mice.

#### 5.4.4 Hepatocellular Carcinoma

Hepatocellular carcinoma (HCC) represents the most prevalent form of primary liver cancer, constituting between 75 and 85% of cases. It is marked by a high morbidity and mortality rate, with the majority of patients encountering a lack of surgical candidates due to delayed diagnosis and treatment facing challenges such as an immunosuppressive microenvironment and drug resistance [216]. Within the TME of HCC, TAMs function as pivotal immunomodulators, orchestrating disease progression through polarization (M1/M2 phenotypic switch) and intricate intercellular interactions [217].

In the context of HCC, M1 has been shown to impede tumor progression through a variety of mechanisms [218, 219, 220]. Current research endeavors have centered on the regulation of genes or proteins capable of inducing macrophage polarization or infiltration. For instance, stromal cell protein spondin2 (SPON2) has been shown to promote M1‐like macrophage infiltration by activating RhoA and Rac1 and increasing F‐actin reorganization through SPON2‐α4β1 integrin signaling [221]. Conversely, high expression of sirtuin1 (SIRT1) in HCC cells has been observed to regulate M1 polarization through the NF‐κB pathway [222]. Furthermore, elevated expression of retinoic acid‐inducible gene I (RIG‐I) has been shown to induce apoptosis in HCC by promoting M1 polarization in mouse peritoneal macrophages via the RIG‐I/MAVS/TRAF2/NF‐κB pathway [223]. IL‐12‐hyperexpressing monocytes exhibit suppressed phosphorylation of STAT3 (p‐STAT3) and diminished c‐Myc signaling activity, a molecular reprogramming that drives macrophage polarization toward proinflammatory M1 states while exerting tumor‐suppressive effects on HCC progression [224]. Furthermore, activated (M2) macrophages have been observed to secrete the cytokine CCL22, which has been implicated in enhancing tumor invasion and inducing EMT through Smad2/3 and Smad1/5/8 activation and Snail upregulation [225]. Furthermore, CCL17, another cytokine secreted by M2 macrophages, has been shown to be closely associated with tumor stemness and EMT in the TGFβ1 and Wnt/β‐catenin signaling pathways [226].

#### 5.4.5 Cholangiocarcinoma

Cholangiocarcinoma (CCA) is a highly aggressive malignant neoplasm that originates from the epithelium of the bile ducts. CCA is characterized by difficulties in early diagnosis, poor prognosis, and limited therapeutic options. The M2‐polarized TAM population in TME demonstrates bifunctional capacity: First, through immunosuppressive cytokine secretion (notably IL‐10/TGF‐β), they establish an immune‐evasive milieu; second, via sustained production of proangiogenic mediators (VEGF) and MMPs, they actively potentiate CCA growth, local infiltration, and distant metastasis. Furthermore, the interaction between TAM and CCA cells can form an immunosuppressive microenvironment, thereby weakening the efficacy of chemotherapy and immunotherapy. Consequently, targeting the polarization state or signaling pathway of TAM has emerged as a novel strategy for CCA treatment.

Experimental evidence reveals M2‐polarized TAMs remodel the immune landscape of intrahepatic CCA (iCCA) through multifactorial mechanisms: cytokine release (including TNF‐α, ICAM‐1, IL‐6) and cancer cell EMT induction [227]. Complementary in vivo studies demonstrate iCCA‐derived factors accelerate M2 differentiation of human monocytic leukemia‐derived THP‐1 cells, with resultant IL‐10‐secreting macrophages potentiating HCC proliferation, metastatic propensity, and mesenchymal transition cascades [228]. Macrophage population and phenotype are positively correlated with angiogenesis and clinical prognosis in HCC. CD14 + CD16 + monocytes from CCA patients express high levels of angiogenic factor‐related genes (epithelial regulatory proteins, VEGF‐A and CXCL3) and predict tissue invasiveness in iCCA [229]. The angiogenic cascade in neoplasms is mechanistically linked to macrophage‐derived metalloelastase (HME) and VEGF signaling pathways. Intriguingly, M2‐polarized TAMs generated through ICC‐secreted cytokine stimulation exhibit dual proangiogenic effects: enhanced microvessel density and transcriptional upregulation of VEGF biosynthesis [230].

### 5.5 Cardiovascular Disease

Cardiovascular disease (CVD) is a complex group of disorders characterized by atherosclerosis, myocardial infarction (MI), and stroke, with core pathological mechanisms involving vascular endothelial damage, lipid deposition, chronic inflammation, and immune cell infiltration. Macrophages are the major immune cell type within atherosclerotic plaques. They play a dual role in CVD through phenotypic polarization. In particular, macrophages with a TAM‐like phenotype (similar to M2 type) may promote tissue repair in atherosclerotic plaques by secreting antiinflammatory factors and stabilize plaques by inhibiting inflammation through phagocytosis of apoptotic cells. In contrast, M1‐type macrophages release proinflammatory factors and MMPs, accelerating the risk of plaque rupture. Recent studies have focused on modulating macrophage polarization homeostasis or targeting their metabolic reprogramming (e.g., cholesterol metabolism, glycolysis) to inhibit plaque progression and stabilize the vascular microenvironment, providing new strategies for the treatment of CVD.

Angiopoietin‐like proteins 3 (ANGPTL3) demonstrates potent atherogenesis‐promoting activity through multitiered mechanisms. Akt pathway activation via phosphorylation mediates ANGPTL3‐driven upregulation of TLR4, potentiating robust NF‐κB nuclear translocation upon LPS challenge. This signaling cascade culminates in proinflammatory cytokine biosynthesis and M1‐skewed polarization in human THP‐1 macrophages. In vivo validation using ANGPTL3‐overexpressing murine models reveals amplified TLR4‐mediated plaque inflammation driving atherosclerotic lesion progression [231].

The mTOR signaling network serves as a multifunctional regulator of cellular homeostasis, coordinating processes including growth regulation, metabolic adaptation, and survival pathways [232]. While mTOR hyperactivation demonstrates pathological correlations with atherogenesis, its cell‐specific contributions to plaque evolution and macrophage functionality remain mechanistically elusive. Paradoxically, whereas pharmacological mTORC1 inhibition confers vascular protection [233], genetic ablation of mTORC2 in macrophages precipitates exacerbated atherosclerotic manifestations characterized by complex lesion morphology and elevated apoptotic indices [234]. In vitro mechanistic studies reveal mTORC2‐mediated suppression of FoxO1 transcriptional activity, effectively constraining inflammasome assembly and IL‐1β production—central mediators of vascular inflammation. Importantly, pharmacological FoxO1 antagonism demonstrates therapeutic efficacy in mitigating inflammation driven by mTORC2 deficiency across experimental models. Notably, the collective deletion of macrophage mTOR disrupts mTORC1‐ and mTORC2‐dependent pathways, resulting in minimal changes in plaque size or complexity, suggesting a balanced yet opposing role of these signaling arms [231].

25‐Hydroxycholesterol (25‐HC) is an oxidized product of cholesterol, produced by the enzyme cholesterol 25‐hydroxylase. Classified within a family of bioactive oxysterols, 25‐HC is endogenously synthesized by cells during cholesterol homeostasis dysregulation and immunostimulation [235]. Macrophage‐derived 25‐HC exacerbates atherosclerotic progression through dual mechanisms: autocrine/paracrine‐mediated plaque destabilization and intraplaque smooth muscle cell migration impairment. Mechanistically, 25‐HC potentiates proinflammatory activation in lipid‐engorged macrophages while restricting vascular smooth muscle cell motility. Experimental investigations further reveal 5‐HC modulates membrane cholesterol dynamics, disrupting TLR4 signaling architecture to amplify NF‐κB‐driven inflammatory transcriptomes and augment apoptotic susceptibility in lesional macrophages [236].

## 6 Potential Therapeutic Targets to Cure Liver Injury by Modulating Macrophages

An additional crucial function of macrophage polarization in liver injury, specific pathways, and chemokines within macrophages significantly impacts the progression of NAFLD, MASH, ALF, and acute‐on‐chronic liver failure (ACLF). The following therapeutic targets may be explored for the modulation of macrophages to facilitate the treatment of liver injury (Table 2).

**TABLE 2:** Different drugs at different targets in liver injury.

Targets	Names	Effects related to MASH or distinguishing features	Clinical trial stage	References
CCR2/CCR5	Cenicriviroc	Antifibrotic effects in animal models, potential fibrosis regression	III	[307]
	Gemcabene	Anti‐inflammation, decrease LDL‐C, CRP, IL‐6, IL‐1 levels, significant reduction in fibrosis progression	III	[308, 309]
PPAR	Lanifibranor	Improve liver enzymes, with more frequent diarrhea, nausea, anemia, etc. than placebo group	III	[310, 311, 312]
	Saroglitazar	Improve ALT and LFC, improvement in insulin resistance and atherogenic dyslipidemia	Launched	[313, 314]
	MBX 8025	Adverse effects: more transaminase elevation at higher doses	II	[315]
	PXL065	Improve adiponectin level, with no peripheral oedema or anemia	IV	[316]
	Pioglitazone	Improve insulin resistance and lipid metabolism	II	[317]
GLP‐1R	Semaglutide	No significant difference in patients with improvement in fibrosis stage, great improvements in liver biochemistry and steatosis combined with semaglutide and firsocostat	III	[318, 319]
	Liraglutide	Enhance insulin secretion, inhibit glucagon secretion anti‐inflammatory and antioxidant	III	[320]
FXR	Obeticholic acid	Fibrosis improvement, with pruritus more frequently	III	[321]
	Cilofexor	Great improvements in liver biochemistry and steatosis combined with semaglutide and firsocostat	II	[318]
	Tropifexor	Sustained decreases in ALT and HFF, with pruritus more frequently	II	[322]
Galectin‐3	Belapectin	Safe but no significant reduction in fibrosis	II	[323]
THR‐β	Resmetirom	Significant reduction in hepatic fat, expedited approval from the US FDA	III	[324]
FGF21	Pegozafermin	Clinically meaningful reductions in liver fat, measures of liver function, and circulating lipids	II	[325, 326]
	Efruxifermin	Reduce HFF and liver injury markers, with longer‐duration studies warranted	II	[327]
SCD	Aramchol	The primary endpoint of a reduction in liver fat did not meet the prespecified significance level with Aramchol 600 mg	III	[328]
ASK1	Selonsertib	No significant effect of liver biomarkers	III	[215]
ACC	Firsocostat	Great improvements in liver biochemistry and steatosis combined with semaglutide and firsocostat	II	[318]
	Clesacostat	The first clinical research to assess histological endpoints using diacylglycerol acyltransferase 2 inhibitor (DGAT2i) and DGAT2i + acetyl‐coenzyme A carboxylase (ACC) inhibitor	II	[238, 329]
DGAT‐2			Ervogasta	[238]

### 6.1 CCL2, CCR2/5 Antagonist

In MASLD, macrophages are recognized as playing a critical role, with elevated periportal macrophages serving as an early histological indicator. Specifically, periportal CCR2^+^ inflammatory macrophages accumulate in the periportal areas of patients with MASH, which correlates with the severity of the disorder and the presence of fibrosis [237]. Upon activation, macrophages develop secretory competence, enabling the production of a diverse array of immunomodulatory molecules. Notably, these include chemokines such as CCL2 (MCP‐1), CCL3 (MIP‐1α), and CXCL8 (IL‐8), as well as proinflammatory cytokines like IL‐1β, IL‐6, and TNF‐α. This multifaceted repertoire of mediators plays a crucial role in orchestrating the pathological inflammatory cascades that underlie disease pathogenesis.

A notable trend of upregulation in chemokines and their receptors has been identified in patients diagnosed with MASH. This trend encompasses the upregulation of CCL3‐5/CCR‐5, as well as the chemokine CCL2. Furthermore, hepatic expression of additional cytokines, such as CD44 and CD62E (E‐Selectin), has been significantly elevated in MASH patients, indicating their involvement in leukocyte recruitment to inflammatory sites. CD44 interacts with components of the ECM, including osteopontin, to regulate macrophage recruitment to the liver [238]. Additionally, it plays a role in macrophage activation triggered by DAMPs, PAMPs, and saturated fatty acids. Given that the CCL2/CCR2 axis is crucial for recruitment of inflammatory monocytes to the injured liver and for driving the progression of hepatic fibrosis, various studies have explored strategies to mitigate MASH‐induced liver injury by blocking this axis. The administration of CCL2 inhibitors in mouse models of steatohepatitis induced by a methionine–choline‐deficient diet and chronic liver injury (CCl4‐induced) has demonstrated a reduction in monocyte/macrophage infiltration and an improvement in MASH progression [239].

Cenicriviroc (CVC), an orally bioavailable dual chemokine receptor antagonist targeting CCR2/CCR5 with optimal pharmacokinetic properties, was originally developed for HIV management [240] through suppression of Ly6C^+^ monocyte infiltration, CVC depletes hepatic macrophage reservoirs, consequently attenuating fibrotic progression in chronic liver diseases [241]. Clinical validation across randomized placebo‐controlled studies and phase IIb trials confirms its therapeutic efficacy in fibrosis, demonstrating acceptable tolerability and sustained hepatic benefits.

Previous studies have investigated the function of CCR2 in concanavalin A‐induced immune liver damage with a particular focus on the dual CCR2/CCR5 antagonist, CVC. Demonstrated to be safe in clinical trials and effective in various animal disease models, CVC was evaluated for its effects on liver macrophage populations. Flow cytometry analysis revealed that CVC treatment significantly reduced the overall proportion of liver macrophages in Con A‐administered mice, specifically decreasing the levels of MoMFs while leaving KCs unaffected. CVC intervention in Con A‐challenged murine models elicited marked depletion of hepatic proinflammatory CX3CR1^+^CCR2^+^Ly6C^+^ macrophage subpopulations. Complementary immunohistochemical quantification revealed attenuated CCR2+ monocyte accumulation in therapeutic cohorts. These congruent findings establish CVC's dual mechanism, suppression of CCR2^+^ monocyte activation, and blockade of hepatic leukocyte trafficking. The therapeutic efficacy of CVC was also assessed through the measurement of serum transaminase levels and the evaluation of liver tissue necrosis, both of which demonstrated a positive response, indicating that CVC significantly mitigates liver injury induced by Con A. Complementary experimental evidence from TUNEL assays and ROS histochemical detection revealed CVC‐mediated attenuation of hepatocellular apoptosis and oxidative damage in Con A‐challenged murine livers. Extended pharmacological investigations validate CVC's multifunctional hepatoprotective profile, demonstrating antifibrotic efficacy and steatosis suppression across diverse hepatic pathologies [242, 243]. Overall, these findings validate CVC's pharmacological promise in mitigating Con A‐triggered immune‐mediated hepatic injury.

In murine studies, pharmacological targeting of the CCL2–CCR2 pathway has been shown to reduce monocyte infiltration and the accumulation of MoMF in the injured liver. Consequently, the use of CCL2 inhibitors, in conjunction with CCR2/5 antagonists, has the potential to enhance fibrosis outcomes by diminishing monocyte infiltration and modifying hepatic macrophage subsets. The CCR2/5 pathway has emerged as a prominent goal in the treatment of MASH [244].

### 6.2 PPAR Agonist

PPARs are ligand‐activated core transcription factors that adjust adipocyte disintegration, lipid storage, and insulin sensitivity. This transcription factor facilitates hepatic lipogenesis and steatosis, promotes fatty acid absorption and triglyceride synthesis in hepatocytes, and enhances the secretion of adipokines via SREBP‐1c [245]. Conversely, PPAR‐γ also protects the liver by inhibiting the proinflammatory polarization of macrophages, thereby reducing oxidative stress and reversing the activation of HSCs [246, 247].

The inactivation of PPAR expression results in the activation of HSCs, an inflammatory response, and increased lipogenesis; conversely, the activation of PPARs can prevent and suppress liver fibrosis. Notably, targeted therapies aimed at activating PPAR expression have demonstrated potential efficacy in the treatment of hepatic diseases associated with liver fibrosis [248].

Multiple synthetic PPAR‐α agonists have undergone rigorous clinical assessment. Notably, fenofibrate exhibited therapeutic efficacy in ameliorating histopathological features within experimental murine steatohepatitis models [249]. Concurrently, bezafibrate administration demonstrated dual pharmacodynamic effects—modulating lipometabolic pathways and restoring hepatic architecture in monosodium glutamate‐induced rodent models substantiating its therapeutic potential for MASLD management [250].

Certain synthetic PPAR‐γ agonists have been shown to mitigate liver inflammation and activate HSCs. Pioglitazone, in particular, has the potential to improve hepatic steatosis, inflammation, and fibrosis in MASLD by downregulating platelet‐derived growth factor and TIMP‐2 [251]. Pioglitazone demonstrates geroprotective potential with therapeutic implications for senescent hepatic fibrogenesis [251]. Mechanistic parallels emerge for rosiglitazone, which ameliorates acetaminophen‐induced acute hepatotoxicity while attenuating diet/age‐driven hepatic lipid accumulation and MASH progression [252, 253]. Notably, the PPARβ/δ agonist KD3010 exerts ECM remodeling inhibition in experimental fibrosis models (CCL4 cholestatic), achieved through dual suppression of HSC activation and inflammatory cascade potentiation [254]. L‐165041, a synthetic PPAR‐β/δ ligand, has also been shown to prevent hepatic steatosis by ameliorating liver inflammation and lipid accumulation in mice subjected to a Western diet [255]. Consequently, there is a pressing need to explore additional PPAR‐β/δ agonists for the treatment of liver diseases. Experimental evidence of Wy‐14,643‐mediated oxidative damage attenuation implicates PPARα‐regulated genomic targets that modulate hepatic redox homeostasis and/or sustain mitochondrial β‐oxidative flux as critical mediators of APAP hepatoprotection [255]. Further support for the hypothesis that Wy‐14,643 mediates protection against ischemia–reperfusion injury (IRI) in fatty livers through the attenuation of the inflammatory response has been provided by studies examining adhesion molecule expression.

Research has revealed a previously unrecognized hepatoprotective mechanism mediated by PPAR‐α during acetaminophen‐induced liver injury. In this experimental paradigm, prophylactic treatment with the synthetic PPAR‐α activator Wy‐14,643 effectively prevented all signs of hepatic injury in genetically unmodified mice following administration of a hepatotoxic APAP dosage. The safeguarding effect was confirmed through multiple parameters: preserved macroscopic hepatic appearance, microscopic evaluation of hematoxylin–eosin‐stained tissue samples showing absence of notable pathological alterations, and substantially reduced plasma concentrations of hepatocellular enzymes (AST/ALT). Furthermore, in the context of IRI, pretreatment with Wy‐14,643 demonstrated protective effects in both MASH and steatosis models, leading to a significant reduction in ALT levels and areas of hepatocellular necrosis [256, 257].

### 6.3 Glucagon‐Like Peptide‐1 Receptor Agonists

Gut‐derived hormones are currently utilized in the clinical management of metabolic diseases, exemplified by glucagon‐like peptide‐1 receptor (GLP‐1R) agonists such as semaglutide and liraglutide, as well as GLP‐1/GIP (glucose‐dependent insulinotropic polypeptide) dual agonists like tirzepatide. Additionally, GLP‐1/glucagon dual agonists, including cotadutide, are under advanced clinical development [258, 259]. Their influence on liver inflammation and fibrosis is primarily considered to be indirect, potentially through mechanisms such as reduced energy supply and improved hepatocyte metabolism. Sort of evidence proposes that hepatic KCs may possess functional GLP‐1 receptors, implying possible susceptibility to pharmacological activation through GLP‐1R agonists [260]. Of particular clinical relevance, therapeutic targeting of this pathway has demonstrated efficacy in achieving histological improvement of MASH in phase II clinical trials [261], findings that have stimulated expanded clinical evaluation of this molecular mechanism in subsequent large‐scale investigations. Furthermore, tirzepatide exhibits a pronounced dose‐dependent effect on body weight reduction, particularly when compared with semaglutide and dulaglutide [262]. Given this body of evidence, certain GLP‐1R agonists and tirzepatide are emerging as promising therapeutic options for MASLD and MASH, especially in individuals with concurrent type 2 diabetes mellitus or obesity [263, 264].

The GLP‐1R demonstrates extensive expression throughout multiple anatomical systems, spanning pancreatic tissue, central nervous networks, gastrointestinal components, cardiovascular structures, hepatic parenchyma, adipose deposits, and musculoskeletal elements [265]. Binding of GLP‐1 to its receptor stimulates insulin secretion while inhibiting glucagon secretion, leading to improved glucose metabolism and lower blood glucose levels. Furthermore, GLP‐1R plays a role in regulating gastric emptying, gastric acid secretion, and gastric motility; it also suppresses food and water intake, thereby contributing to weight loss. Recently, GLP‐1R agonists and GLP‐1R/glucagon receptor (GCGR) dual‐target agonists have demonstrated efficacy in the treatment of MASH [266].

Studies have evaluated the potential beneficial effects of GLP‐1R and GCGR dual agonists on liver fibrosis in rodent models. These dual‐target agonists have been shown to improve liver conditions in rats and mice with CCl4‐induced hepatic fibrosis. The underlying mechanism involves the inhibition of TGF‐β and the downstream Smad signaling pathways, which ultimately contribute to the reversal of hepatic fibrosis [266]. Furthermore, the histological features of MASH, such as steatosis and ballooning, have also been found to improve with the administration of GLP‐1R agonists, including liraglutide and semaglutide [262]. Experimental evidence illustrates liraglutide's capacity to regulate immune cell differentiation patterns. Demonstrated through preclinical models, the GLP‐1R agonist lixisenatide ameliorates atherosclerotic pathology in insulin‐resistant murine subjects through phenotypic alteration of macrophages [267]. Research by Bruen et al. has documented liraglutide's dual capacity to direct monocyte‐derived cell phenotypes while influencing cellular differentiation toward M2‐resolution specialized macrophages [268. Complementing these findings, in vitro investigations have revealed liraglutide administration induces antiinflammatory polarization of KCs [269].

There is emerging evidence suggesting that GLP‐1RAs may confer benefits in liver fibrosis associated with MASLD. While the precise mechanisms by which GLP‐1RAs may reduce liver fibrosis remain unclear, several experimental studies indicate that these agents could modulate ECM homeostasis. This regulatory mechanism appears to be mediated via dual suppression of oxidative stress mediators and downregulation of MAPK cascade activity, thereby impeding the assembly of BATF/JUN transcriptional complexes involving basic leucine zipper ATF‐like transcription factors [270]. However, it remains uncertain whether this mechanism is applicable to human livers [271]. A recent systematic review and network meta‐analysis highlighted that semaglutide, liraglutide, and the combination of vitamin E with pioglitazone exhibited the highest probability of ranking as the most effective interventions for achieving resolution of MASH [272].

### 6.4 Thyroid Hormone Receptor‐Beta Agonist

As previously mentioned, PPARs, liver X receptors, FXRs, and thyroid hormone receptors (THRs) are recognized as prominent therapeutic target receptors. While FXRs have been extensively studied, this review will primarily focus on THRs, with a particular emphasis on THR‐β. THR‐β is predominantly expressed in the liver, and THR‐β agonists have shown efficacy in reducing LDL levels, TG, and hepatic steatosis in humans. Additionally, THR‐β facilitates fatty acid catabolism and stimulates mitochondrial biogenesis, resulting in decreased lipotoxicity and enhanced liver function, which ultimately leads to a reduction in liver fat [273].

Resmetirom (MGL‐3196) represents a novel investigational orally bioavailable selective THR‐β agonist with pioneering therapeutic potential [274]. Data from mechanistic studies implicate macrophage‐mediated processes in its anti‐MASH activity [275]. Practically positioned downstream of PI3K/AKT signaling, the NF‐κB cascade constitutes a principal regulatory axis for inflammatory mediators, whose aberrant activation is fundamentally linked to MASH pathogenesis. Concurrently, STAT3 stimulation exerts context‐dependent immunomodulatory effects, demonstrating dual functionality in both fibrogenic progression and inflammatory modulation during MASH evolution [274]. Through Gene Ontology and Kyoto Encyclopedia of Genes and Genomes enrichment analyses conducted in both the resmetirom and control groups, Wang et al. [276] suggest that resmetirom has the potential to ameliorate MASH by restoring the expression of RGS5, which subsequently deactivates the STAT3 and NF‐κB signaling pathways.

### 6.5 Fibroblast Growth Factor 21

Fibroblast growth factor 21 (FGF21) is a member of the FGF superfamily and serves as an endocrine factor closely related to FGF19 and FGF23, which are secreted by the liver to regulate the intake of simple sugars and the preference for sweet foods [277]. This regulatory function is mediated through signaling via FGF21 receptors located in the paraventricular nucleus of the hypothalamus. FGF21 promotes thermogenesis through endocrine signaling to adipose tissue, which helps prevent adipose tissue dysfunction and reduces lipid spillage into the liver [278]. Additionally, it enhances fatty acid oxidation and cholesterol clearance through autocrine signaling to the liver, thereby mitigating hepatic lipotoxicity [279]. Furthermore, FGF21 inhibits the activation of KCs, monocyte recruitment, and the formation of LAMs and SAMs, which may impede collagen accumulation and reduce hepatic fibrosis. Recent studies have demonstrated that FGF21 can diminish alcohol preference and cravings, leading to a reduction in alcoholic liver injury [280]. Consequently, FGF21 represents a promising target for mitigating alcohol‐induced hepatic damage.

During the development of MASH, evidence suggests that FGF21 inhibits the activation of KCs and MoMFs. This inhibition prevents the accumulation of CD36^high^ KCs and CD36^high^CD9^high^ MoMFs under dietary conditions that promote MASH. These findings indicate that FGF21 reduces LAMs and SAMs, thereby inhibiting the synthesis of collagen type I alpha 1 and preventing fibrogenesis [281].

Distinct from canonical fibroblast growth factors, FGF21 exhibits absent heparin affinity, enabling systemic circulation as an endocrine mediator. This pleiotropic hormone exerts profound regulatory effects on whole‐body metabolic homeostasis while demonstrating hepatoprotective capacities [282]. Preclinical and clinical investigations collectively validate FGF21's cytoprotective function in hepatic systems [283]. Experimental models employing FGF21‐deficient rodents fed lipotoxic diets display aggravated hepatocellular injury, a phenotype rescued through exogenous FGF21 administration [284, 285]. Mechanistic studies further reveal FGF21's capacity to attenuate carbon tetrachloride‐induced acute hepatic damage via SIRT1‐dependent autophagic induction [285]. Clinical observations demonstrate significantly elevated circulating FGF21 concentrations in ACLF patients, showing positive correlations with hepatic injury biomarkers including CRP and transaminases. Particularly, FGF21 transcription is regulated by CHOP—a terminal effector of endoplasmic reticulum (ER) stress cascades [286]. Paradoxically, increased FGF21 expression reciprocally inhibits the eIF2α–ATF4–CHOP signaling axis, creating negative feedback to mitigate ER stress‐mediated hepatocyte injury [281]. Beyond ER stress modulation, oxidative challenges induce dramatic upregulation of both hepatic FGF21 production and systemic secretion. This adaptive response potentially represents the liver's endogenous mechanism to bolster antioxidant defenses and counteract toxic insults [287]. Moreover, clinical evidence indicates diverse changes in serum FGF21 levels among HBV‐infected patients under varying clinical conditions. Serum levels of FGF21 were positively correlated with serum aminotransferases, suggesting that FGF21 may serve as an indicator of liver damage associated with HBV infection. CHB patients demonstrate diminished circulating FGF21 concentrations, particularly pronounced in cases of HBV‐induced cirrhotic complications, a phenomenon potentially reflecting impaired hepatic biosynthetic capabilities. Conversely, the paradoxical FGF21 surge detected in CHB subjects progressing to HCC suggests diagnostic value for this hepatokine in tracking malignant transformation within this clinical cohort [288].

The intrinsic elevation of FGF21 across multiple pathological states positions this hepatokine as a compelling candidate for emerging as a multipurpose diagnostic and prognostic biomarker. Clinical investigations revealed a 25‐fold surge in circulating FGF21 concentrations within 2 h posttransplantation, whereas maximal ALT/AST concentrations emerged at 24‐h postprocedural intervals [289]. Temporal trajectory analysis identified concordance between 2‐h FGF21 peaks and subsequent 24‐h transaminase elevations, establishing its potential as an early‐warning indicator with high predictive accuracy for IRI in transplant recipients [290]. While further mechanistic investigations and population‐specific validation remain prerequisites for clinical implementation, this emerging pleiotropic hormone exhibits substantial therapeutic potential for both managing and monitoring diverse metabolic pathologies.

The therapeutic potential of natural FGF21 is constrained by its short half‐life of approximately 2 h. Consequently, researchers are actively developing a long‐acting glycosylated and polyglycolated FGF21 analogue known as pegozafermin, which exhibits an extended in vivo half‐life. This analogue demonstrates promise for the treatment of MASH and severe hypertriglyceridemia. In clinical trials, pegozafermin successfully achieved both primary endpoints, indicating potential efficacy in treating MASH. Notably, all three dosage groups (15 mg or 30 mg weekly, or 44 mg biweekly) exhibited one phase of improvement in defibrase, without exacerbation of MASH or worsening of fibrosis, and did so without significant side effects [279, 291].

### 6.6 Galectin‐3 Antagonist

Galectin‐3 (Gal ‐ 3), a protein of significant importance, is instrumental in the pathogenesis of MASH and fibrosis, as well as in various fibrotic conditions affecting the lungs, heart, kidneys, liver, and vascular system. It interacts with receptors such as TLR4, TGF ‐ β, dectin‐1, and TREM2, leading to a range of intracellular effects. Within cells, Galectin‐3 is associated with AKT phosphorylation, transcriptional regulation, Wnt/β‐catenin signaling, and the regulation of apoptosis. Extracellularly, it plays a role in inflammation, cell proliferation, differentiation, and migration. Additionally, Galectin‐3 promotes morphogenesis in endothelial cells and facilitates tumor angiogenesis [292].

As an upstream factor of TREM2, Gal‐3 act a pivotal part in modulating macrophage polarization, specifically reducing M1 polarization while promoting M2 polarization [293]. Additionally, Gal‐3 activates NLRP3 inflammatory vesicles by interacting with TLR4 in the liver through its carbohydrate‐recognition‐binding domains, thereby facilitating the development and expression of inflammatory cells. This research finds a significant association between Gal‐3 and progression of chronic MASH in mice subjected to a high‐fat diet [294].

Accumulating research identifies Gal‐3 as a pathogenic factor in hepatic fibrogenesis, where pharmacological blockade demonstrates therapeutic efficacy against fibrotic resolution [295, 296]. The multifactorial pathogenesis of tissue fibrogenesis involves intricate crosstalk between immunocytes and activated myofibroblasts, with Gal‐3 emerging as a pivotal molecular bridge [294]. Functioning as a pleiotropic modulator, Gal‐3 orchestrates cellular responses in both stromal (myofibroblasts) and innate immune compartments (macrophages), critically influencing fibrotic progression. Models of carbon tetrachloride‐induced hepatic fibrosis reveal that genetic ablation of Gal‐3 suppresses stellate cell transdifferentiation and collagen biosynthesis, demonstrating impaired TGF‐β responsiveness in Gal‐3‐deficient cells compared with wild‐type counterparts. Mechanistically, Gal‐3 deficiency disrupts TGF‐β receptor membrane localization while attenuating β‐catenin phosphorylation and nuclear trafficking, though Smad2/3 activation remains unaffected. These findings establish Gal‐3's indispensable role in TGF‐β‐driven ECM remodeling through noncanonical signaling pathways. Specifically, Gal‐3 mediates IL‐4‐dependent macrophage polarization toward profibrotic phenotypes [297], with IL‐4/IL‐13‐stimulated macrophages amplifying fibrogenic gene networks that perpetuate collagen deposition and disease advancement. This molecular triad positions Gal‐3 as a critical nexus integrating macrophage–fibroblast interactions within the fibrotic microenvironment. A deeper understanding of the mechanisms regulating tissue fibrosis, along with targeted strategies to inhibit Gal‐3 in the liver, provides a compelling rationale for developing novel therapeutic approaches for patients suffering from liver fibrosis.

Galectin therapeutics has engineered GR‐MD‐02, a modified pectic polysaccharide derived from Malus domestica, through synthetic modification processes. This galactoarabino–rhamnogalacturonan polymer contains a heterogeneous sugar composition dominated by galacturonic acid, galactose, arabinose, and rhamnose residues. Preclinical analyses demonstrate its superior binding specificity for Gal‐3 over related galectin isoforms. Initial clinical evaluation in a 2015 Phase 1 trial (NCT01899859) involving 31 MASH patients with progressive fibrosis demonstrated acceptable safety profiles and biological activity. Subsequent Phase 2 investigations comprised two parallel cohorts: the MASH FX study (NCT02421094, *n* = 30) enrolling subjects with significant fibrosis (F3) and the larger MASH CX trial (NCT02462967, *n* = 162) focusing on cirrhotic patients. Despite these extensive evaluations, GR‐MD‐02 failed to achieve statistically significant improvements in the primary histological endpoint of fibrosis regression through quantitative MRI‐based assessment (LiverMultiSca). Secondary biomarkers including magnetic resonance elastography and vibration‐controlled transient elastography (FibroScan) measurements similarly showed no clinically meaningful alterations. The pleiotropic involvement of Gal‐3 in chronic inflammatory pathologies has solidified its status as a viable therapeutic target across multiple disease states. This molecular rationale has stimulated substantial commercial investment, with numerous biopharmaceutical entities pursuing diverse targeting approaches including small molecule inhibitors, monoclonal antibodies, and carbohydrate‐based antagonists. These strategies include the design and synthesis of powerful small molecule antagonists (such as glycomimetics) and larger biologics derived from natural sources, all aimed at therapeutic intervention for various cancers, fibrosis, and other liver diseases [298]. Gal3‐targeting therapies may be utilized either as standalone treatments or in combination with existing medications.

The pivotal involvement of Gal‐3 in orchestrating inflammatory cascades and fibrotic remodeling across multiple organ systems has positioned its pharmacological targeting as a priority in novel therapeutic development. Preclinical investigations and emerging clinical evidence collectively substantiate the antifibrotic potential of Gal‐3 blockade. In concanavalin A‐challenged murine hepatitis models, prophylactic administration of the Gal‐3 antagonist TD139 attenuated hepatic inflammatory responses through immunomodulatory mechanisms [299]. DAVANAT—a carbohydrate‐based Gal‐3 inhibitor demonstrated therapeutic efficacy in experimental cholangitis induced by Novosphingobium aromaticivorans [300] and dextran sulfate sodium‐mediated colitis [301] via suppression of NLRP3 inflammasome activation and IL‐1β downregulation in macrophage populations. Further supporting this therapeutic paradigm, the orally bioavailable Gal‐3 inhibitor GB1211 exhibited dual antifibrotic activity in murine models of hepatotoxic (CCl4‐induced) and pulmonary (bleomycin‐induced) fibrosis through distinct pathway modulation [302]. Clinically, elevated Gal‐3 expression correlates with hepatic decompensation risks, particularly in cirrhotic progression and chronic HBV infections. Patients with HBV‐associated ACLF displaying Gal‐3 promoter methylation exhibited accelerated disease trajectories characterized by reduced survival durations, elevated 3‐month mortality indices, and higher MELD prognostic scores [303]. Moreover, given the involvement of Gal‐3 in nearly all inflammatory processes activated during acute intravascular hemolysis, this molecule may represent a viable therapeutic target. Future research is essential to elucidate the precise role of Gal‐3 in the development of acute and chronic tissue injuries caused by acute intravascular hemolysis and to further explore its previously demonstrated therapeutic effects in fibrotic diseases, thereby positioning Gal‐3 as a potential therapeutic target in hemolysis‐induced organ damage.

Furthermore, investigations have revealed temporal elevation of Gal‐3 expression during initial hepatic insult phases following α‐galactosylceramide administration, detected systemically in circulation and hepatic compartments. Immunohistochemical analyses identified Gal‐3‐expressing macrophages migrating to inflammatory foci with distinct aggregation patterns. These observations propose macrophage‐derived Gal‐3 as a promising diagnostic indicator for acute hepatocellular damage in histopathological evaluations [304].

Numerous medications are available for the management of liver inflammation. Inhibitors of apoptosis signal‐regulating kinase 1 (ASK1) () have shown efficacy in halting disease progression, which encompasses inflammation, liver fibrosis, insulin resistance, and hepatic lipid accumulation [305, 306]. Additionally, agonists of the A3 adenosine receptor can induce apoptosis in inflammatory cells and inhibit the NF‐κB signaling pathway. Below outlines the various drugs, their corresponding targets, and their clinical trial statuses (Table 2).

## 7 Conclusion and Prospectives

As indicated above, macrophages, a pivotal component of the immune system, fulfill a variety of functions. These include maintaining body homeostasis, regulating immune responses, and participating in numerous disease processes. The complexity and diversity of their signaling pathways determine the plasticity and functional diversity of macrophages in different microenvironments. A comprehensive examination of these pathways and their roles in health and disease will facilitate a more profound comprehension of their functions in physiological and pathological processes. This, in turn, will provide new targets and strategies for the treatment of various diseases.

Signaling pathways such as JAK–STAT, NF‐κB, and PI3K–Akt–mTOR play a pivotal role in the regulation of macrophage polarization and function, influencing the proinflammatory (M1) and antiinflammatory (M2) phenotypic transitions of macrophages by regulating the activities of key transcription factors. For instance, the JAK–STAT pathway drives M1‐ and M2‐type polarization through the phosphorylation of STAT1 and STAT6, respectively. The NF‐κB pathway promotes M1‐type proinflammatory responses and M2‐type tissue repair functions through classical and nonclassical pathways, respectively. The PI3K–Akt–mTOR pathway affects macrophage energy acquisition and functional status through metabolic reprogramming.

Maladaptive polarization patterns and functional impairment of macrophages represent key pathophysiological hallmarks across various pathological conditions. In autoimmune disorders such as SLE and RA, disturbances in the homeostatic equilibrium of macrophages, specifically the M1/M2 ratio, drive pathogenic cascades by triggering excessive immune responses and resulting in parenchymal injury. In neurodegenerative contexts like AD, prolonged activation of microglial populations—macrophages residing in the CNS—perpetuates neuroinflammatory cascades through the continuous release of proinflammatory mediators. In TMEs, TAMs promote neoplastic progression and immune evasion via paracrine signaling of proinflammatory cytokines and angiogenic factors. Cardiovascular pathologies illustrate the role of macrophages in regulating atherosclerotic plaque dynamics, where the states of polarization critically influence lesion stability and pathological evolution.

Despite the relatively deep understanding of macrophage signaling pathways and their roles in disease, many questions remain to be further investigated, and future research needs to be focused on. With the development of new technologies, future studies can utilize these technologies to deeply resolve macrophage signaling pathways and their roles in disease. For example, single‐cell sequencing technology can help us more precisely identify the heterogeneity and functional status of macrophages in different disease states. While advances in basic research have identified numerous therapeutic candidates through the elucidation of mechanisms, significant translational barriers remain in clinical implementation. Enhancing the continuity of translation between experimental models and human trials is vital for the advancement of precision treatment paradigms. Pharmacological agents that modulate key signaling nodes, such as the JAK–STAT and NF‐κB pathways, have demonstrated preclinical efficacy across various disease models. However, these agents necessitate rigorous molecular optimization and multicenter validation to establish their clinical relevance.

## Author Contributions

Yongquan Chi and Haipeng Jiang: writing—original draft; Yongquan Chi, Haipeng Jiang, Jianhua Rao, and Yongsheng Li: writing—review and editing; Yiyuan Yin, Xinyu Zhou, and Yiyouyou Shao: formal analysis and resources; Jianhua Rao and Yongsheng Li: conceptualization; Jianhua Rao: funding acquisition. All authors have read and approved the final manuscript.

## Ethics Statement

The authors have nothing to report.

## Conflicts of Interest

The authors disclose no conflicts of interest.

## Data Availability

No data were used for the research described in the article.

## References

[1] S. Su , J. Zhao , Y. Xing , et al., “Immune Checkpoint Inhibition Overcomes ADCP‐induced Immunosuppression by Macrophages,” Cell 175, no. 2 (2018): 442–457. e23. 30290143 10.1016/j.cell.2018.09.007

[2] V. Kiseleva , P. Vishnyakova , A. Elchaninov , et al., “Biochemical and Molecular Inducers and Modulators of M2 Macrophage Polarization in Clinical Perspective,” International Immunopharmacology 122 (2023): 110583. 37423155 10.1016/j.intimp.2023.110583

[3] D. M. Underhill , S. Gordon , B. A. Imhof , et al., “Élie Metchnikoff (1845–1916): Celebrating 100 Years of Cellular Immunology and Beyond,” Nature Reviews Immunology 16, no. 10 (2016): 651–656. 10.1038/nri.2016.89 27477126

[4] I. Vitale , G. Manic , L. M. Coussens , et al., “Macrophages and Metabolism in the Tumor Microenvironment,” Cell Metabolism 30, no. 1 (2019): 36–50. 31269428 10.1016/j.cmet.2019.06.001

[5] The Lancet . Editorial: Ballon v. bladder. The Lancet 1974;2(7873):141–142. 4135508

[6] T. A. Wynn and K. M. Vannella , “Macrophages in Tissue Repair, Regeneration, and Fibrosis,” Immunity 44, no. 3 (2016): 450–462. 26982353 10.1016/j.immuni.2016.02.015 PMC4794754

[7] L. Honold and M. Nahrendorf , “Resident and Monocyte‐derived Macrophages in Cardiovascular Disease,” Circulation Research 122, no. 1 (2018): 113–127. 29301844 10.1161/CIRCRESAHA.117.311071 PMC5777215

[8] D. van der Heide , R. Weiskirchen , and R. Bansal , “Therapeutic Targeting of Hepatic Macrophages for the Treatment of Liver Diseases,” Frontiers in Immunology 10 (2019): 2852. 31849997 10.3389/fimmu.2019.02852 PMC6901832

[9] M. Guilliams , F. Ginhoux , C. Jakubzick , et al., “Dendritic Cells, Monocytes and Macrophages: A Unified Nomenclature Based on Ontogeny,” Nature Reviews Immunology 14, no. 8 (2014): 571–578. 10.1038/nri3712 PMC4638219 25033907

[10] R. M. Rodrigues , J. Boeckmans , and T. Vanhaecke , “Macrophages Clear out Necrotic Liver Lesions: A New Magic Trick Revealed,” Egastroenterology 1, no. 2 (2023): e100024. 39944003 10.1136/egastro-2023-100024 PMC11741187

[11] S. A. MacParland , J. C. Liu , X.‐Z. Ma , et al., “Single Cell RNA Sequencing of human Liver Reveals Distinct Intrahepatic Macrophage Populations,” Nature Communications 9, no. 1 (2018): 4383. 10.1038/s41467-018-06318-7 PMC6197289 30348985

[12] E. Papalexi and R. Satija , “Single‐cell RNA Sequencing to Explore Immune Cell Heterogeneity,” Nature Reviews Immunology 18, no. 1 (2018): 35–45. 10.1038/nri.2017.76 28787399

[13] Y. Wu and K. K. Hirschi , “Tissue‐resident Macrophage Development and Function,” Frontiers in Cell and Developmental Biology 8 (2020): 617879. 33490082 10.3389/fcell.2020.617879 PMC7820365

[14] A. Guillot and F. Tacke , “Spatial Dimension of Macrophage Heterogeneity in Liver Diseases,” Egastroenterology 1, no. 1 (2023): e000003. 39944246 10.1136/egastro-2023-000003 PMC11770455

[15] Y. Ma , J. Wang , Y. Wang , et al., “The Biphasic Function of Microglia in Ischemic Stroke,” Progress in Neurobiology 157 (2017): 247–272. 26851161 10.1016/j.pneurobio.2016.01.005

[16] A. Dey , J. Allen , and P. A. Hankey‐Giblin , “Ontogeny and Polarization of Macrophages in Inflammation: Blood Monocytes versus Tissue Macrophages,” Frontiers in Immunology 5 (2014): 683. 25657646 10.3389/fimmu.2014.00683 PMC4303141

[17] M. Delfini , N. Stakenborg , M. F. Viola , et al., “Macrophages in the Gut: Masters in Multitasking,” Immunity 55, no. 9 (2022): 1530–1548. 36103851 10.1016/j.immuni.2022.08.005

[18] M. Casanova‐Acebes , E. Dalla , A. M. Leader , et al., “Tissue‐resident macrophages provide a pro‐tumorigenic niche to early NSCLC cells,” Nature 595, no. 7868 (2021): 578–584. 10.1038/s41586-021-03651-8. 34135508 PMC8923521

[19] H. Ding , J.‐J. Wang , X.‐Y. Zhang , et al., “Lycium Barbarum Polysaccharide Antagonizes LPS‐induced Inflammation by Altering the Glycolysis and Differentiation of Macrophages by Triggering the Degradation of PKM2,” Biological & Pharmaceutical Bulletin 44, no. 3 (2021): 379–388. 33390389 10.1248/bpb.b20-00752

[20] P.‐S. Liu , Y.‐T. Chen , X. Li , et al., “CD40 signal Rewires Fatty Acid and Glutamine Metabolism for Stimulating Macrophage Anti‐tumorigenic Functions,” Nature Immunology 24, no. 3 (2023): 452–462. 36823405 10.1038/s41590-023-01430-3 PMC9977680

[21] H. Guo , Y. Song , F. Li , et al., “ACT001 suppressing M1 Polarization Against Inflammation via NF‐κB and STAT1 Signaling Pathways Alleviates Acute Lung Injury in Mice,” International Immunopharmacology 110 (2022): 108944. 35728304 10.1016/j.intimp.2022.108944

[22] A. Sica and A. Mantovani , “Macrophage Plasticity and Polarization: In Vivo Veritas,” Journal of Clinical Investigation 122, no. 3 (2012): 787–795. 22378047 10.1172/JCI59643 PMC3287223

[23] J. Rao , H. Wang , M. Ni , et al., “FSTL1 promotes Liver Fibrosis by Reprogramming Macrophage Function Through Modulating the Intracellular Function of PKM2,” Gut 71, no. 12 (2022): 2539–2550. 35140065 10.1136/gutjnl-2021-325150 PMC9664121

[24] Q. Zhang and M. Sioud , “Tumor‐associated Macrophage Subsets: Shaping Polarization and Targeting,” International Journal of Molecular Sciences 24, no. 8 (2023): 7493. 37108657 10.3390/ijms24087493 PMC10138703

[25] M. Stein , S. Keshav , N. Harris , et al., “Interleukin 4 Potently Enhances Murine Macrophage Mannose Receptor Activity: A Marker of Alternative Immunologic Macrophage Activation,” Journal of Experimental Medicine 176, no. 1 (1992): 287–292. 1613462 10.1084/jem.176.1.287 PMC2119288

[26] L. Lefèvre , A. Galès , D. Olagnier , et al., “PPARγ Ligands Switched High Fat Diet‐induced Macrophage M2b Polarization Toward M2a Thereby Improving Intestinal Candida Elimination,” PLoS ONE 5, no. 9 (2010): e12828. 20877467 10.1371/journal.pone.0012828 PMC2942900

[27] A. H. Sikkema , J. M. J. Stoffels , P. Wang , et al., “Fibronectin Aggregates Promote Features of a Classically and Alternatively Activated Phenotype in Macrophages,” Journal of Neuroinflammation 15, no. 1 (2018): 218. 30071854 10.1186/s12974-018-1238-x PMC6091019

[28] R. L. Gieseck , M. S. Wilson , and T. A. Wynn , “Type 2 Immunity in Tissue Repair and Fibrosis,” Nature Reviews Immunology 18, no. 1 (2018): 62–76. 10.1038/nri.2017.90 28853443

[29] H. Li , Y. Meng , S. He , et al., “Macrophages, Chronic Inflammation, and Insulin Resistance,” Cells 11, no. 19 (2022): 3001. 36230963 10.3390/cells11193001 PMC9562180

[30] L.‐X. Wang , S.‐X. Zhang , H.‐J. Wu , et al., “M2b macrophage Polarization and Its Roles in Diseases,” Journal of Leukocyte Biology 106, no. 2 (2019): 345–358. 30576000 10.1002/JLB.3RU1018-378RR PMC7379745

[31] Q. Cao , N. Wang , J. Qi , et al., “Long Non‑Coding RNA‑GAS5 Acts as a Tumor Suppressor in Bladder Transitional Cell Carcinoma via Regulation of Chemokine (C‑C motif) Ligand 1 Expression,” Molecular Medicine Reports 13, no. 1 (2016): 27–34. 26548923 10.3892/mmr.2015.4503 PMC4686088

[32] A. Asai , K. Nakamura , M. Kobayashi , et al., “CCL1 released From M2b Macrophages Is Essentially Required for the Maintenance of Their Properties,” Journal of Leukocyte Biology 92, no. 4 (2012): 859–867. 22730547 10.1189/jlb.0212107

[33] A. Shapouri‐Moghaddam , S. Mohammadian , H. Vazini , et al., “Macrophage Plasticity, Polarization, and Function in Health and Disease,” Journal of Cellular Physiology 233, no. 9 (2018): 6425–6440. 10.1002/jcp.26429 PMC13482560 29319160

[34] J. Null , Y.‐S. Lai , H. T. Nguyen , et al., “MERTK+/Hi M2c Macrophages Induced by baicalin Alleviate Non‐alcoholic Fatty Liver Disease,” International Journal of Molecular Sciences 22, no. 19 (2021): 10604. 34638941 10.3390/ijms221910604 PMC8508959

[35] M. Horckmans , L. Ring , J. Duchene , et al., “Neutrophils Orchestrate Post‐myocardial Infarction Healing by Polarizing Macrophages towards a Reparative Phenotype,” European Heart Journal 38, no. 3 (2017): 187–197. 28158426 10.1093/eurheartj/ehw002

[36] X. Huang , Y. Li , M. Fu , et al. Polarizing Macrophages in vitro. In: Rousselet G , editor. New York, NY: Springer New York. “Methods in Molecular Biology” (New York, NY): Springer New York, (2018): 119–126. 10.1007/978-1-4939-7837-3_12 PMC8875934 29761394

[37] G. Zizzo , B. A. Hilliard , M. Monestier , et al., “Efficient Clearance of Early Apoptotic Cells by human Macrophages Requires M2c Polarization and MerTK Induction,” Journal of Immunology 189, no. 7 (2012): 3508–3520. 10.4049/jimmunol.1200662 PMC3465703 22942426

[38] Q. Wang , H. Ni , L. Lan , et al., “Fra‐1 Protooncogene Regulates IL‐6 Expression in Macrophages and Promotes the Generation of M2d Macrophages,” Cell Research 20, no. 6 (2010): 701–712. 20386569 10.1038/cr.2010.52

[39] W. Lai , C. Xian , M. Chen , et al., “Single‐cell and Bulk Transcriptomics Reveals M2d Macrophages as a Potential Therapeutic Strategy for Mucosal Healing in Ulcerative Colitis,” International Immunopharmacology 121 (2023): 110509. 37369160 10.1016/j.intimp.2023.110509

[40] X. Hu , J. Li , M. Fu , et al., “The JAK/STAT Signaling Pathway: From Bench to Clinic,” Signal Transduction and Targeted Therapy 6, no. 1 (2021): 402. 34824210 10.1038/s41392-021-00791-1 PMC8617206

[41] S. Zhang , Y. Meng , L. Zhou , et al., “Targeting Epigenetic Regulators for Inflammation: Mechanisms and Intervention Therapy,” MedComm 3, no. 4 (2022): e173. 36176733 10.1002/mco2.173 PMC9477794

[42] L. B. Ivashkiv , “IFNγ: Signalling, Epigenetics and Roles in Immunity, Metabolism, Disease and Cancer Immunotherapy,” Nature Reviews Immunology 18, no. 9 (2018): 545–558. 10.1038/s41577-018-0029-z PMC6340644 29921905

[43] K. Kang , M. Bachu , S. H. Park , et al., “IFN‐γ Selectively Suppresses a Subset of TLR4‐activated Genes and Enhancers to Potentiate Macrophage Activation,” Nature Communications 10, no. 1 (2019): 3320. 10.1038/s41467-019-11147-3 PMC6658531 31346169

[44] D. E. Johnson , R. A. O'Keefe , and J. R. Grandis , “Targeting the IL‐6/JAK/STAT3 Signalling Axis in Cancer,” Nature Reviews Clinical oncology 15, no. 4 (2018): 234–248. 10.1038/nrclinonc.2018.8 PMC5858971 29405201

[45] M. S. Hayden and S. Ghosh , “Shared Principles in NF‐kappaB Signaling,” Cell 132, no. 3 (2008): 344–362. 18267068 10.1016/j.cell.2008.01.020

[46] C. Liu , F. Hu , G. Jiao , et al., “Dental Pulp Stem Cell‐derived Exosomes Suppress M1 Macrophage Polarization Through the ROS‐MAPK‐NFκB P65 Signaling Pathway After Spinal Cord Injury,” Journal of Nanobiotechnology 20, no. 1 (2022): 65. 35109874 10.1186/s12951-022-01273-4 PMC8811988

[47] H.‐F. Zhou , C. Yang , J.‐Y. Li , et al., “Quercetin Serves as the Major Component of Xiang‐lian Pill to Ameliorate Ulcerative Colitis via Tipping the Balance of STAT1/PPARγ and Dictating the Alternative Activation of Macrophage,” Journal of Ethnopharmacology 313 (2023): 116557. 37142141 10.1016/j.jep.2023.116557

[48] T. Zhang , C. Ma , Z. Zhang , et al., “NF‐κB Signaling in Inflammation and Cancer,” MedComm 2, no. 4 (2021): 618–653. 34977871 10.1002/mco2.104 PMC8706767

[49] V. Y.‐F. Wang , Y. Li , D. Kim , et al., “Bcl3 phosphorylation by Akt, Erk2, and IKK Is Required for Its Transcriptional Activity,” Molecular Cell 67, no. 3 (2017): 484–497. e5. 28689659 10.1016/j.molcel.2017.06.011 PMC6571149

[50] S. Khanmohammadi and M. S. Kuchay , “Toll‐Like Receptors and Metabolic (dysfunction)‐associated Fatty Liver Disease,” Pharmacological Research 185 (2022): 106507. 36252773 10.1016/j.phrs.2022.106507

[51] K. Miura , E. Seki , H. Ohnishi , et al., “Role of Toll‐Like Receptors and Their Downstream Molecules in the Development of Nonalcoholic Fatty Liver Disease,” Gastroenterology Research and Practice 2010 (2010): 362847. 21274430 10.1155/2010/362847 PMC3026974

[52] J. Petrasek , T. Csak , and G. Szabo , “Toll‐Like Receptors in Liver Disease,” Advances in Clinical Chemistry 59 (2013): 155–201. 23461136 10.1016/b978-0-12-405211-6.00006-1

[53] C. S. Mantsounga , C. Lee , J. Neverson , et al., “Macrophage IL‐1β Promotes Arteriogenesis by Autocrine STAT3‐ and NF‐κB‐mediated Transcription of Pro‐angiogenic VEGF‐A,” Cell Reports 38, no. 5 (2022): 110309. 35108537 10.1016/j.celrep.2022.110309 PMC8865931

[54] C. Porta , M. Rimoldi , G. Raes , et al., “Tolerance and M2 (alternative) Macrophage Polarization Are Related Processes Orchestrated by p50 Nuclear Factor kappaB,” PNAS 106, no. 35 (2009): 14978–14983. 19706447 10.1073/pnas.0809784106 PMC2736429

[55] Y. Zhang , M. Xia , K. Jin , et al., “Function of the c‐Met Receptor Tyrosine Kinase in Carcinogenesis and Associated Therapeutic Opportunities,” Molecular Cancer 17, no. 1 (2018): 45. 29455668 10.1186/s12943-018-0796-y PMC5817860

[56] T. Ersahin , N. Tuncbag , and R. Cetin‐Atalay , “The PI3K/AKT/mTOR Interactive Pathway,” Molecular Biosystems 11, no. 7 (2015): 1946–1954. 25924008 10.1039/c5mb00101c

[57] S.‐J. Zhao , F.‐Q. Kong , J. Jie , et al., “Macrophage MSR1 Promotes BMSC Osteogenic Differentiation and M2‐Like Polarization by Activating PI3K/AKT/GSK3β/β‐catenin Pathway,” Theranostics 10, no. 1 (2020): 17–35. 31903103 10.7150/thno.36930 PMC6929615

[58] J. Liu , F. Xing , Q. Fu , et al., “hUC‐MSCs Exosomal miR‐451 Alleviated Acute Lung Injury by Modulating Macrophage M2 Polarization via Regulating MIF‐PI3K‐AKT Signaling Pathway,” Environmental Toxicology 37, no. 12 (2022): 2819–2831. 35997581 10.1002/tox.23639

[59] Y. Wang , X. Zhang , J. Wang , et al., “Inflammatory Periodontal Ligament Stem Cells Drive M1 Macrophage Polarization via Exosomal miR‐143‐3p‐mediated Regulation of PI3K/AKT/NF‐κB Signaling,” Stem Cells 41, no. 2 (2023): 184–199. 36520505 10.1093/stmcls/sxac087

[60] Z. Zhang , S. Peng , T. Xu , et al., “Retinal Microenvironment‐protected rhein‐GFFYE Nanofibers Attenuate Retinal Ischemia‐reperfusion Injury via Inhibiting Oxidative Stress and Regulating Microglial/Macrophage M1/M2 Polarization,” Advancement of Science 10, no. 30 (2023): 2302909. 10.1002/advs.202302909 PMC10602545 37653617

[61] X. Xu , L. Cui , L. Zhang , et al., “Saikosaponin D Modulates the Polarization of Tumor‐associated Macrophages by Deactivating the PI3K/AKT/mTOR Pathway in Murine Models of Pancreatic Cancer,” International Immunopharmacology 122 (2023): 110579. 37433245 10.1016/j.intimp.2023.110579

[62] T. Lawrence and G. Natoli , “Transcriptional Regulation of Macrophage Polarization: Enabling Diversity With Identity,” Nature Reviews Immunology 11, no. 11 (2011): 750–761. 10.1038/nri3088 22025054

[63] T. Tamura , H. Yanai , D. Savitsky , et al., “The IRF family Transcription Factors in Immunity and Oncogenesis,” Annual Review of Immunology 26 (2008): 535–584. 10.1146/annurev.immunol.26.021607.090400 18303999

[64] R. Zhang , Q. Li , P. Y. Chuang , et al., “Regulation of Pathogenic Th17 Cell Differentiation by IL‐10 in the Development of Glomerulonephritis,” American Journal of Pathology 183, no. 2 (2013): 402–412. 23747510 10.1016/j.ajpath.2013.05.001 PMC3730759

[65] Z. Ahmad , W. Kahloan , and E. D. Rosen , “Transcriptional Control of Metabolism by Interferon Regulatory Factors,” Nature reviews Endocrinology 20, no. 10 (2024): 573–587. 10.1038/s41574-024-00990-0 PMC11392651 38769435

[66] R. Günthner and H.‐J. Anders , “Interferon‐regulatory Factors Determine Macrophage Phenotype Polarization,” Mediators of Inflammation 2013 (2013): 731023. 24379524 10.1155/2013/731023 PMC3863528

[67] Y.‐J. Chen , J. Li , N. Lu , et al., “Interferon Regulatory Factors: A Key to Tumour Immunity,” International Immunopharmacology 49 (2017): 1–5. 28531759 10.1016/j.intimp.2017.05.010

[68] M. Sharifiaghdam , E. Shaabani , R. Faridi‐Majidi , et al., “Macrophages as a Therapeutic Target to Promote Diabetic Wound Healing,” Molecular Therapy 30, no. 9 (2022): 2891–2908. 35918892 10.1016/j.ymthe.2022.07.016 PMC9482022

[69] G. Lu , R. Zhang , S. Geng , et al., “Myeloid Cell‐derived Inducible Nitric Oxide Synthase Suppresses M1 Macrophage Polarization,” Nature Communications 6 (2015): 6676. 10.1038/ncomms7676 PMC4389243 25813085

[70] J. Eguchi , X. Kong , M. Tenta , et al., “Interferon Regulatory Factor 4 Regulates Obesity‐induced Inflammation Through Regulation of Adipose Tissue Macrophage Polarization,” Diabetes 62, no. 10 (2013): 3394–3403. 23835343 10.2337/db12-1327 PMC3781469

[71] I. Espinoza and L. Miele , “Deadly Crosstalk: Notch Signaling at the Intersection of EMT and Cancer Stem Cells,” Cancer Letters 341, no. 1 (2013): 41–45. 23973264 10.1016/j.canlet.2013.08.027

[72] R. A. Kovall , B. Gebelein , D. Sprinzak , et al., “The Canonical Notch Signaling Pathway: Structural and Biochemical Insights Into Shape, Sugar, and Force,” Developmental Cell 41, no. 3 (2017): 228–241. 28486129 10.1016/j.devcel.2017.04.001 PMC5492985

[73] P. Kongkavitoon , P. Butta , A. Sanpavat , et al., “Regulation of Periostin Expression by Notch Signaling in Hepatocytes and Liver Cancer Cell Lines,” Biochemical and Biophysical Research Communications 506, no. 3 (2018): 739–745. 30384995 10.1016/j.bbrc.2018.10.144

[74] S. F. Aval , H. Lotfi , R. Sheervalilou , et al., “Tuning of Major Signaling Networks (TGF‐β, Wnt, Notch and Hedgehog) by miRNAs in human Stem Cells Commitment to Different Lineages: Possible Clinical Application,” Biomedicine & Pharmacotherapy 91 (2017): 849–860. 28501774 10.1016/j.biopha.2017.05.020

[75] G. Wu , G. Wilson , J. George , et al., “Modulation of Notch Signaling as a Therapeutic Approach for Liver Cancer,” Current Gene Therapy 15, no. 2 (2015): 171–181. 25537772 10.2174/1566523214666141224100319

[76] E. Keewan and S. A. Naser , “The Role of Notch Signaling in Macrophages During Inflammation and Infection: Implication in Rheumatoid Arthritis?,” Cells 9, no. 1 (2020): 111. 31906482 10.3390/cells9010111 PMC7016800

[77] W. Wongchana , P. Kongkavitoon , P. Tangtanatakul , et al., “Notch Signaling Regulates the Responses of Lipopolysaccharide‐stimulated Macrophages in the Presence of Immune Complexes,” PLoS ONE 13, no. 6 (2018): e0198609. 29889863 10.1371/journal.pone.0198609 PMC5995379

[78] E. Keewan and S. A. Naser , “Notch‐1 Signaling Modulates Macrophage Polarization and Immune Defense Against Mycobacterium Avium Paratuberculosis Infection in Inflammatory Diseases,” Microorganisms 8, no. 7 (2020): 1006. 32635645 10.3390/microorganisms8071006 PMC7409363

[79] H. Xu , J. Zhu , S. Smith , et al., “Notch‐RBP‐J Signaling Regulates the Transcription Factor IRF8 to Promote Inflammatory Macrophage Polarization,” Nature Immunology 13, no. 7 (2012): 642–650. 22610140 10.1038/ni.2304 PMC3513378

[80] Y.‐C. Wang , F. He , F. Feng , et al., “Notch Signaling Determines the M1 versus M2 Polarization of Macrophages in Antitumor Immune Responses,” Cancer Research 70, no. 12 (2010): 4840–4849. 20501839 10.1158/0008-5472.CAN-10-0269

[81] W. Guo , Z. Li , G. Anagnostopoulos , et al., “Notch Signaling Regulates Macrophage‐mediated Inflammation in Metabolic Dysfunction‐associated Steatotic Liver Disease,” Immunity 57, no. 10 (2024): 2310–2327. e6. 39317200 10.1016/j.immuni.2024.08.016

[82] F. Vieceli Dalla Sega , F. Fortini , G. Aquila , et al., “Notch Signaling Regulates Immune Responses in Atherosclerosis,” Frontiers in immunology 10 (2019): 1130. 31191522 10.3389/fimmu.2019.01130 PMC6540611

[83] J. Xu , F. Chi , and H. Tsukamoto , “Notch Signaling and M1 Macrophage Activation in Obesity‐alcohol Synergism,” Clinics and Research in Hepatology and Gastroenterology 39, no. Suppl 10 1 (2015): S24–S28. 26189984 10.1016/j.clinre.2015.05.016 PMC4656026

[84] S. Gordon , “Phagocytosis: An Immunobiologic Process,” Immunity 44, no. 3 (2016): 463–475. 26982354 10.1016/j.immuni.2016.02.026

[85] M. A. Sanjuan , C. P. Dillon , S. W. G. Tait , et al., “Toll‐Like Receptor Signalling in Macrophages Links the Autophagy Pathway to Phagocytosis,” Nature 450, no. 7173 (2007): 1253–1257. 18097414 10.1038/nature06421

[86] D. Li , R. Wei , X. Zhang , et al., “Gut Commensal Metabolite Rhamnose Promotes Macrophages Phagocytosis by Activating SLC12A4 and Protects Against Sepsis in Mice,” Acta Pharmaceutica Sinica B 14, no. 7 (2024): 3068–3085. 39027244 10.1016/j.apsb.2024.03.025 PMC11252530

[87] S. Duan and J. C. Paulson , “Siglecs as Immune Cell Checkpoints in Disease,” Annual Review of Immunology 38 (2020): 365–395. 10.1146/annurev-immunol-102419-035900 31986070

[88] X. Zhuang , J. Ma , S. Xu , et al., “SHP‐1 Suppresses Endotoxin‐induced Uveitis by Inhibiting the TAK1/JNK Pathway,” Journal of Cellular and Molecular Medicine 25, no. 1 (2021): 147–160. 33207073 10.1111/jcmm.15888 PMC7810969

[89] A. A. Barkal , R. E. Brewer , M. Markovic , et al., “CD24 signalling Through Macrophage Siglec‐10 Is a Target for Cancer Immunotherapy,” Nature 572, no. 7769 (2019): 392–396. 31367043 10.1038/s41586-019-1456-0 PMC6697206

[90] J. Á. Freile , N. Ustyanovska Avtenyuk , M. G. Corrales , et al., “CD24 is a Potential Immunotherapeutic Target for Mantle Cell Lymphoma,” Biomedicines 10, no. 5 (2022): 1175. 35625912 10.3390/biomedicines10051175 PMC9138264

[91] S. M. G. Hayat , V. Bianconi , M. Pirro , et al., “CD47: Role in the Immune System and Application to Cancer Therapy,” Cellular Oncology 43, no. 1 (2020): 19–30. 31485984 10.1007/s13402-019-00469-5 PMC12990683

[92] Y. Fujioka , T. Matozaki , T. Noguchi , et al., “A Novel Membrane Glycoprotein, SHPS‐1, That Binds the SH2‐domain‐containing Protein Tyrosine Phosphatase SHP‐2 in Response to Mitogens and Cell Adhesion,” Molecular and Cellular Biology 16, no. 12 (1996): 6887–6899. 8943344 10.1128/mcb.16.12.6887 PMC231692

[93] A. Kharitonenkov , Z. Chen , I. Sures , et al., “A family of Proteins That Inhibit Signalling Through Tyrosine Kinase Receptors,” Nature 386, no. 6621 (1997): 181–186. 9062191 10.1038/386181a0

[94] A. N. Barclay and T. K. Van den Berg , “The Interaction Between Signal Regulatory Protein Alpha (SIRPα) and CD47: Structure, Function, and Therapeutic Target,” Annual Review of Immunology 32 (2014): 25–50. 10.1146/annurev-immunol-032713-120142 24215318

[95] R. K. Tsai and D. E. Discher , “Inhibition of ‘Self’ engulfment Through Deactivation of Myosin‐II at the Phagocytic Synapse Between human Cells,” Journal of Cell Biology 180, no. 5 (2008): 989–1003. 18332220 10.1083/jcb.200708043 PMC2265407

[96] J. F. Timms , K. D. Swanson , A. Marie‐Cardine , et al., “SHPS‐1 Is a Scaffold for Assembling Distinct Adhesion‐regulated Multi‐protein Complexes in Macrophages,” Current Biology 9, no. 16 (1999): 927–930. 10469599 10.1016/s0960-9822(99)80401-1

[97] A. Das , M. Sinha , S. Datta , et al., “Monocyte and Macrophage Plasticity in Tissue Repair and Regeneration,” American Journal of Pathology 185, no. 10 (2015): 2596–2606. 26118749 10.1016/j.ajpath.2015.06.001 PMC4607753

[98] Q. Zhang , M. Raoof , Y. Chen , et al., “Circulating Mitochondrial DAMPs Cause Inflammatory Responses to Injury,” Nature 464, no. 7285 (2010): 104–107. 20203610 10.1038/nature08780 PMC2843437

[99] T. Kisseleva and D. Brenner , “Molecular and Cellular Mechanisms of Liver Fibrosis and Its Regression,” Nature reviews Gastroenterology & Hepatology 18, no. 3 (2021): 151–166. 33128017 10.1038/s41575-020-00372-7

[100] S. Nattel , “Molecular and Cellular Mechanisms of Atrial Fibrosis in Atrial Fibrillation,” JACC: Clinical Electrophysiology 3, no. 5 (2017): 425–435. 29759598 10.1016/j.jacep.2017.03.002

[101] Y. Wei , H. Guo , S. Chen , et al., “Regulation of Macrophage Activation by Lactylation in Lung Disease,” Frontiers in Immunology 15 (2024): 1427739. 39026681 10.3389/fimmu.2024.1427739 PMC11254698

[102] P. M.‐K. Tang , D. J. Nikolic‐Paterson , and H.‐Y. Lan , “Macrophages: Versatile Players in Renal Inflammation and Fibrosis,” Nature Reviews Nephrology 15, no. 3 (2019): 144–158. 30692665 10.1038/s41581-019-0110-2

[103] L. Yan , J. Wang , X. Cai , et al., “Macrophage Plasticity: Signaling Pathways, Tissue Repair, and Regeneration,” MedComm 5, no. 8 (2024): e658. 39092292 10.1002/mco2.658 PMC11292402

[104] L. Guo , H. Akahori , E. Harari , et al., “CD163+ macrophages Promote Angiogenesis and Vascular Permeability Accompanied by Inflammation in Atherosclerosis,” Journal of Clinical Investigation 128, no. 3 (2018): 1106–1124. 29457790 10.1172/JCI93025 PMC5824873

[105] X. Cui , R.‐T. Tan Morales , W. Qian , et al., “Corrigendum to ‘Hacking Macrophage‐associated Immunosuppression for Regulating Glioblastoma Angiogenesis’ [Biomater. 161 (2018) 164‐178],” Biomaterials 287 (2022): 121667. 35797856 10.1016/j.biomaterials.2022.121667

[106] Y. Yang , Z. Guo , W. Chen , et al., “M2 macrophage‐derived Exosomes Promote Angiogenesis and Growth of Pancreatic Ductal Adenocarcinoma by Targeting E2F2,” Molecular Therapy 29, no. 3 (2021): 1226–1238. 33221435 10.1016/j.ymthe.2020.11.024 PMC7934635

[107] X. Guo , H. Zhang , C. He , et al., “RUNX1 promotes Angiogenesis in Colorectal Cancer by Regulating the Crosstalk Between Tumor Cells and Tumor Associated Macrophages,” Biomarker Research 12, no. 1 (2024): 29. 38419056 10.1186/s40364-024-00573-1 PMC10903076

[108] T. Zeng , K. Sun , L. Mai , et al., “Extracellular Vesicle‐associated miR‐ERIA Exerts the Anti‐angiogenic Effect of Macrophages in Diabetic Wound Healing,” Diabetes 74, no. 4 (2025): 596–610. 39854218 10.2337/db24-0701 PMC11926273

[109] R. Ramalho , M. Rao , C. Zhang , et al., “Immunometabolism: New Insights and Lessons From Antigen‐directed Cellular Immune Responses,” Seminars in Immunopathology 42, no. 3 (2020): 279–313. 32519148 10.1007/s00281-020-00798-w PMC7282544

[110] N. Luque‐Campos , F. A. Bustamante‐Barrientos , C. Pradenas , et al., “The Macrophage Response Is Driven by Mesenchymal Stem Cell‐mediated Metabolic Reprogramming,” Frontiers in Immunology 12 (2021): 624746. 34149687 10.3389/fimmu.2021.624746 PMC8213396

[111] E. M. Pålsson‐McDermott and L. A. J. O'Neill , “Targeting Immunometabolism as an Anti‐inflammatory Strategy,” Cell Research 30, no. 4 (2020): 300–314. 32132672 10.1038/s41422-020-0291-z PMC7118080

[112] M. Lacroix , R. Riscal , G. Arena , et al., “Metabolic Functions of the Tumor Suppressor p53: Implications in Normal Physiology, Metabolic Disorders, and Cancer,” Molecular Metabolism 33 (2020): 2–22. 31685430 10.1016/j.molmet.2019.10.002 PMC7056927

[113] K. Mehla and P. K. Singh , “Metabolic Regulation of Macrophage Polarization in Cancer,” Trends in cancer 5, no. 12 (2019): 822–834. 31813459 10.1016/j.trecan.2019.10.007 PMC7187927

[114] J. Behmoaras , “The Versatile Biochemistry of Iron in Macrophage Effector Functions,” Febs Journal 288, no. 24 (2021): 6972–6989. 33354925 10.1111/febs.15682

[115] H. Xiao , Y. Guo , B. Li , et al., “M2‐Like Tumor‐associated Macrophage‐targeted Codelivery of STAT6 Inhibitor and IKKβ siRNA Induces M2‐to‐M1 Repolarization for Cancer Immunotherapy With Low Immune Side Effects,” ACS Central Science 6, no. 7 (2020): 1208–1222. 32724855 10.1021/acscentsci.9b01235 PMC7379385

[116] M. Läsche , G. Emons , and C. Gründker , “Shedding New Light on Cancer Metabolism: A Metabolic Tightrope Between Life and Death,” Frontiers in Oncology 10 (2020): 409. 32300553 10.3389/fonc.2020.00409 PMC7145406

[117] M. Bosch , M. Sánchez‐Álvarez , A. Fajardo , et al., “Mammalian Lipid Droplets Are Innate Immune Hubs Integrating Cell Metabolism and Host Defense,” Science 370, no. 6514 (2020): eaay8085. 33060333 10.1126/science.aay8085

[118] L. Fortuny and C. Sebastián , “Sirtuins as Metabolic Regulators of Immune Cells Phenotype and Function,” Genes 12, no. 11 (2021): 1698. 34828304 10.3390/genes12111698 PMC8618532

[119] S. Chen , A. Saeed , Q. Liu , et al., “Macrophages in Immunoregulation and Therapeutics,” Signal Transduction and Targeted Therapy 8, no. 1 (2023): 207. 37211559 10.1038/s41392-023-01452-1 PMC10200802

[120] L. Yang , M.‐F. Fu , H.‐Y. Wang , et al., “Research Advancements in the Interplay Between T3 and Macrophages,” Current Medical Science 44, no. 5 (2024): 883–889. 39446284 10.1007/s11596-024-2935-6

[121] M. Tang , B. Chen , H. Xia , et al., “pH‐gated Nanoparticles Selectively Regulate Lysosomal Function of Tumour‐associated Macrophages for Cancer Immunotherapy,” Nature Communications 14, no. 1 (2023): 5888. 10.1038/s41467-023-41592-0 PMC10514266 37735462

[122] Y. Wang , Y. Fan , X. Zhang , et al., “In Situ Production and Precise Release of Bioactive GM‐CSF and siRNA by Engineered Bacteria for Macrophage Reprogramming in Cancer Immunotherapy,” Biomaterials 317 (2025): 123037. 39729775 10.1016/j.biomaterials.2024.123037

[123] H. Gao , J. Di , B. H. Clausen , et al., “Distinct Myeloid Population Phenotypes Dependent on TREM2 Expression Levels Shape the Pathology of Traumatic versus Demyelinating CNS Disorders,” Cell Reports 42, no. 6 (2023): 112629. 37289590 10.1016/j.celrep.2023.112629

[124] Y.‐Z. Lu , B. Nayer , S. K. Singh , et al., “CGRP Sensory Neurons Promote Tissue Healing via Neutrophils and Macrophages,” Nature 628, no. 8008 (2024): 604–611. 38538784 10.1038/s41586-024-07237-y PMC11023938

[125] R. Feng , V. Muraleedharan Saraswathy , M. H. Mokalled , et al., “Self‐renewing Macrophages in Dorsal Root Ganglia Contribute to Promote Nerve Regeneration,” PNAS 120, no. 7 (2023): e2215906120. 36763532 10.1073/pnas.2215906120 PMC9963351

[126] C. G. Peace , S. M. O'Carroll , and L. A. J. O'Neill , “Fumarate Hydratase as a Metabolic Regulator of Immunity,” Trends in Cell Biology 34, no. 6 (2024): 442–450. 37940417 10.1016/j.tcb.2023.10.005

[127] A. Hooftman , C. G. Peace , D. G. Ryan , et al., “Macrophage Fumarate Hydratase Restrains mtRNA‐mediated Interferon Production,” Nature 615, no. 7952 (2023): 490–498. 36890227 10.1038/s41586-019-0000-0 PMC10411300

[128] M. Arabpour , A. Saghazadeh , and N. Rezaei , “Anti‐inflammatory and M2 Macrophage Polarization‐promoting Effect of Mesenchymal Stem Cell‐derived Exosomes,” International Immunopharmacology 97 (2021): 107823. 34102486 10.1016/j.intimp.2021.107823

[129] A. Aguzzi , B. A. Barres , and M. L. Bennett , “Microglia: Scapegoat, Saboteur, or Something Else?,” Science 339, no. 6116 (2013): 156–161. 23307732 10.1126/science.1227901 PMC4431634

[130] C. D'Mello , K. Riazi , T. Le , et al., “P‐selectin‐mediated Monocyte‐cerebral Endothelium Adhesive Interactions Link Peripheral Organ Inflammation to Sickness Behaviors,” The Journal of Neuroscience 33, no. 37 (2013): 14878–14888. 24027287 10.1523/JNEUROSCI.1329-13.2013 PMC6705165

[131] C. N. Parkhurst , G. Yang , I. Ninan , et al., “Microglia Promote Learning‐dependent Synapse Formation Through Brain‐derived Neurotrophic Factor,” Cell 155, no. 7 (2013): 1596–1609. 24360280 10.1016/j.cell.2013.11.030 PMC4033691

[132] W. M. Song and M. Colonna , “The Identity and Function of Microglia in Neurodegeneration,” Nature Immunology 19, no. 10 (2018): 1048–1058. 30250185 10.1038/s41590-018-0212-1

[133] F. Matheis , P. A. Muller , C. L. Graves , et al., “Adrenergic Signaling in Muscularis Macrophages Limits Infection‐induced Neuronal Loss,” Cell 180, no. 1 (2020): 64–78. e16. 31923400 10.1016/j.cell.2019.12.002 PMC7271821

[134] X. Yin , K. Kim , H. Suetsugu , et al., “Meta‐analysis of 208370 East Asians Identifies 113 Susceptibility Loci for Systemic Lupus Erythematosus,” Annals of the Rheumatic Diseases 80, no. 5 (2021): 632–640. 33272962 10.1136/annrheumdis-2020-219209 PMC8053352

[135] M. D. Catalina , K. A. Owen , A. C. Labonte , et al., “The Pathogenesis of Systemic Lupus Erythematosus: Harnessing Big Data to Understand the Molecular Basis of Lupus,” Journal of Autoimmunity 110 (2020): 102359. 31806421 10.1016/j.jaut.2019.102359

[136] S. V. Parikh , S. Almaani , S. Brodsky , et al., “Update on Lupus Nephritis: Core Curriculum 2020,” American Journal of Kidney Diseases 76, no. 2 (2020): 265–281. 32220510 10.1053/j.ajkd.2019.10.017

[137] Y. Xin , C. Gao , L. Wang , et al., “Lipopolysaccharide Released From Gut Activates Pyroptosis of Macrophages via Caspase 11‐Gasdermin D Pathway in Systemic Lupus Erythematosus,” MedComm 5, no. 6 (2024): e610. 38881675 10.1002/mco2.610 PMC11176733

[138] C. Deng , Q. Zhang , P. He , et al., “Targeted Apoptosis of Macrophages and Osteoclasts in Arthritic Joints Is Effective Against Advanced Inflammatory Arthritis,” Nature Communications 12, no. 1 (2021): 2174. 10.1038/s41467-021-22454-z PMC8042091 33846342

[139] P. Di Benedetto , P. Ruscitti , Z. Vadasz , et al., “Macrophages With Regulatory Functions, a Possible New Therapeutic Perspective in Autoimmune Diseases,” Autoimmunity Reviews 18, no. 10 (2019): 102369. 31404701 10.1016/j.autrev.2019.102369

[140] L. C. Davies , S. J. Jenkins , J. E. Allen , et al., “Tissue‐resident Macrophages,” Nature Immunology 14, no. 10 (2013): 986–995. 24048120 10.1038/ni.2705 PMC4045180

[141] S. Culemann , A. Grüneboom , J. Á. Nicolás‐Ávila , et al., “Locally Renewing Resident Synovial Macrophages Provide a Protective Barrier for the Joint,” Nature 572, no. 7771 (2019): 670–675. 31391580 10.1038/s41586-019-1471-1 PMC6805223

[142] S. Jain , T.‐H. Tran , and M. Amiji , “Macrophage Repolarization With Targeted Alginate Nanoparticles Containing IL‐10 Plasmid DNA for the Treatment of Experimental Arthritis,” Biomaterials 61 (2015): 162–177. 26004232 10.1016/j.biomaterials.2015.05.028 PMC4464978

[143] N. Jia , Y. Gao , M. Li , et al., “Metabolic Reprogramming of Proinflammatory Macrophages by Target Delivered Roburic Acid Effectively Ameliorates Rheumatoid Arthritis Symptoms,” Signal Transduction and Targeted Therapy 8, no. 1 (2023): 280. 37500654 10.1038/s41392-023-01499-0 PMC10374631

[144] Y. Okabe and R. Medzhitov , “Tissue Biology Perspective on Macrophages,” Nature Immunology 17, no. 1 (2016): 9–17. 26681457 10.1038/ni.3320

[145] S. Watanabe , M. Alexander , A. V. Misharin , et al., “The Role of Macrophages in the Resolution of Inflammation,” Journal of Clinical Investigation 129, no. 7 (2019): 2619–2628. 31107246 10.1172/JCI124615 PMC6597225

[146] J. S. Smolen , D. Aletaha , and I. B. McInnes , “Rheumatoid Arthritis,” The Lancet 388, no. 10055 (2016): 2023–2038. 10.1016/S0140-6736(16)30173-8 27156434

[147] P. P. Tak , T. J. Smeets , M. R. Daha , et al., “Analysis of the Synovial Cell Infiltrate in Early Rheumatoid Synovial Tissue in Relation to Local Disease Activity,” Arthritis and Rheumatism 40, no. 2 (1997): 217–225. 9041933 10.1002/art.1780400206

[148] Y. Hao , S. Hao , E. Andersen‐Nissen , et al., “Integrated Analysis of Multimodal Single‐cell Data,” Cell 184, no. 13 (2021): 3573–3587. e29. 34062119 10.1016/j.cell.2021.04.048 PMC8238499

[149] J. D. Kean , J. Kaufman , J. Lomas , et al., “A Randomized Controlled Trial Investigating the Effects of a Special Extract of Bacopa monnieri (CDRI 08) on Hyperactivity and Inattention in Male Children and Adolescents: BACHI Study Protocol (ANZCTRN12612000827831),” Nutrients 7, no. 12 (2015): 9931–9945. 26633481 10.3390/nu7125507 PMC4690059

[150] X. Wu , J. A. Miller , B. T. K. Lee , et al., “Reducing Microglial Lipid Load Enhances β Amyloid Phagocytosis in an Alzheimer's Disease Mouse Model,” Science Advances 11, no. 6 (2025): eadq6038. 39908361 10.1126/sciadv.adq6038 PMC11797491

[151] Y. Wang , M. Cella , K. Mallinson , et al., “TREM2 lipid Sensing Sustains the Microglial Response in an Alzheimer's disease Model,” Cell 160, no. 6 (2015): 1061–1071. 25728668 10.1016/j.cell.2015.01.049 PMC4477963

[152] A. Griciuc , S. Patel , A. N. Federico , et al., “TREM2 acts Downstream of CD33 in Modulating Microglial Pathology in Alzheimer's Disease,” Neuron 103, no. 5 (2019): 820–835. e7. 31301936 10.1016/j.neuron.2019.06.010 PMC6728215

[153] E. N. Wilson , C. Wang , M. S. Swarovski , et al., “TREM1 disrupts Myeloid Bioenergetics and Cognitive Function in Aging and Alzheimer Disease Mouse Models,” Nature Neuroscience 27, no. 5 (2024): 873–885. 38539014 10.1038/s41593-024-01610-w PMC11102654

[154] R. Dvir‐Szternfeld , G. Castellani , M. Arad , et al., “Alzheimer's Disease Modification Mediated by Bone Marrow‐derived Macrophages via a TREM2‐independent Pathway in Mouse Model of Amyloidosis,” Nat Aging 2, no. 1 (2022): 60–73. 37118355 10.1038/s43587-021-00149-w

[155] S. Moonen , M. J. Koper , E. Van Schoor , et al., “Pyroptosis in Alzheimer's Disease: Cell Type‐specific Activation in Microglia, Astrocytes and Neurons,” Acta Neuropathologica, (Berlin) 145, no. 2 (2023): 175–195. 36481964 10.1007/s00401-022-02528-y

[156] Alzheimer's & Dementia . 2023 Alzheimer's Disease Facts and Figures. Alzheimers Dement 2023;19(4):1598–1695. 36918389 10.1002/alz.13016

[157] S. Su , Q. Liu , J. Chen , et al., “A Positive Feedback Loop Between Mesenchymal‐Like Cancer Cells and Macrophages Is Essential to Breast Cancer Metastasis,” Cancer Cell 25, no. 5 (2014): 605–620. 24823638 10.1016/j.ccr.2014.03.021

[158] C. Wei , C. Yang , S. Wang , et al., “Crosstalk Between Cancer Cells and Tumor Associated Macrophages Is Required for Mesenchymal Circulating Tumor Cell‐mediated Colorectal Cancer Metastasis,” Molecular Cancer 18, no. 1 (2019): 64. 30927925 10.1186/s12943-019-0976-4 PMC6441214

[159] Y. Yin , S. Yao , Y. Hu , et al., “The Immune‐microenvironment Confers Chemoresistance of Colorectal Cancer Through Macrophage‐derived IL6,” Clinical Cancer Research 23, no. 23 (2017): 7375–7387. 28928161 10.1158/1078-0432.CCR-17-1283

[160] X. Wang , G. Luo , K. Zhang , et al., “Hypoxic Tumor‐derived Exosomal miR‐301a Mediates M2 Macrophage Polarization via PTEN/PI3Kγ to Promote Pancreatic Cancer Metastasis,” Cancer Research 78, no. 16 (2018): 4586–4598. 29880482 10.1158/0008-5472.CAN-17-3841

[161] H. Li , P. Yang , J. Wang , et al., “HLF Regulates Ferroptosis, Development and Chemoresistance of Triple‐negative Breast Cancer by Activating Tumor Cell‐macrophage Crosstalk,” Journal of Hematology & Oncology 15, no. 1 (2022): 2. 34991659 10.1186/s13045-021-01223-x PMC8740349

[162] E. Timperi , P. Gueguen , M. Molgora , et al., “Lipid‐associated Macrophages Are Induced by Cancer‐associated Fibroblasts and Mediate Immune Suppression in Breast Cancer,” Cancer Research 82, no. 18 (2022): 3291–3306. 35862581 10.1158/0008-5472.CAN-22-1427

[163] J. Xia , L. Zhang , X. Peng , et al., “IL1R2 blockade Alleviates Immunosuppression and Potentiates Anti‐PD‐1 Efficacy in Triple‐negative Breast Cancer,” Cancer Research 84, no. 14 (2024): 2282–2296. 38657120 10.1158/0008-5472.CAN-23-3429

[164] S. Singh , N. Lee , D. A. Pedroza , et al., “Chemotherapy Coupled to Macrophage Inhibition Induces T‐cell and B‐cell Infiltration and Durable Regression in Triple‐negative Breast Cancer,” Cancer Research 82, no. 12 (2022): 2281–2297. 35442423 10.1158/0008-5472.CAN-21-3714 PMC9219596

[165] Y. Yan , D. Sun , J. Hu , et al., “Multi‐omic Profiling Highlights Factors Associated With Resistance to Immuno‐chemotherapy in Non‐small‐cell Lung Cancer,” Nature Genetics 57, no. 1 (2025): 126–139. 39658657 10.1038/s41588-024-01998-y

[166] G. Chiesa , M. Torre , M. Ravini , et al., “[Agenesis of the right pulmonary artery and systemic pulmonary vascularization in 4 cases observed in childhood],” Minerva Chirurgica 42, no. 1–2 (1987): 39–44. 3561828

[167] C. Wang , Y. Li , L. Wang , et al., “SPP1 represents a Therapeutic Target That Promotes the Progression of Oesophageal Squamous Cell Carcinoma by Driving M2 Macrophage Infiltration,” British Journal of Cancer 130, no. 11 (2024): 1770–1782. 38600327 10.1038/s41416-024-02683-x PMC11130281

[168] Y. Zheng , Z. Chen , Y. Han , et al., “Immune Suppressive Landscape in the human Esophageal Squamous Cell Carcinoma Microenvironment,” Nature Communications 11, no. 1 (2020): 6268. 10.1038/s41467-020-20019-0 PMC7722722 33293583

[169] H. Sakamoto , Y.‐I. Koma , N. Higashino , et al., “PAI‐1 Derived From Cancer‐associated Fibroblasts in Esophageal Squamous Cell Carcinoma Promotes the Invasion of Cancer Cells and the Migration of Macrophages,” Laboratory Investigation; A Journal of Technical Methods and Pathology 101, no. 3 (2021): 353–368. 33311557 10.1038/s41374-020-00512-2 PMC7892342

[170] X. Zhou , Z. Yan , J. Hou , et al., “The Hippo‐YAP Signaling Pathway Drives CD24‐mediated Immune Evasion in Esophageal Squamous Cell Carcinoma via Macrophage Phagocytosis,” Oncogene 43, no. 7 (2024): 495–510. 38168654 10.1038/s41388-023-02923-z PMC10857940

[171] G. Taha , L. Ezra , and N. Abu‐Freha , “Hepatitis C Elimination: Opportunities and Challenges in 2023,” Viruses. 15, no. 7 (2023): 1413. 37515101 10.3390/v15071413 PMC10386528

[172] D. Lavanchy , “Evolving Epidemiology of hepatitis C Virus,” Clinical Microbiology and Infection 17, no. 2 (2011): 107–115. 21091831 10.1111/j.1469-0691.2010.03432.x

[173] C. P. Chan , H. Uemura , T. H. Kwan , et al., “Review on the Molecular Epidemiology of Sexually Acquired hepatitis C Virus Infection in the Asia‐Pacific region,” Journal of the International AIDS Society 23, no. 9 (2020): e25618. 32969173 10.1002/jia2.25618 PMC7511596

[174] C. Trépo , H. L. Y. Chan , and A. Lok , “Hepatitis B Virus Infection,” The Lancet 384, no. 9959 (2014): 2053–2063. 10.1016/S0140-6736(14)60220-8 24954675

[175] O. Paccoud , L. Surgers , and K. Lacombe , “[Hepatitis B virus infection: Natural history, clinical manifestations and therapeutic approach],” Revue de Médecine Interne 40, no. 9 (2019): 590–598. 30982550 10.1016/j.revmed.2019.03.333

[176] C.‐H. Liu and J.‐H. Kao , “Acute hepatitis C Virus Infection: Clinical Update and Remaining Challenges,” Clinical and Molecular Hepatology 29, no. 3 (2023): 623–642. 36800699 10.3350/cmh.2022.0349 PMC10366792

[177] D. I. Chigbu , R. Loonawat , M. Sehgal , et al., “Hepatitis C Virus Infection: Host^−^Virus Interaction and Mechanisms of Viral Persistence,” Cells 8, no. 4 (2019): 376. 31027278 10.3390/cells8040376 PMC6523734

[178] M. H. Nguyen , G. Wong , E. Gane , et al., “Hepatitis B Virus: Advances in Prevention, Diagnosis, and Therapy,” Clinical Microbiology Reviews 33, no. 2 (2020): e00046–19. 10.1128/CMR.00046-19 PMC7048015 32102898

[179] C. L. Day , G. M. Lauer , G. K. Robbins , et al., “Broad Specificity of Virus‐specific CD4+ T‐helper‐cell Responses in Resolved hepatitis C Virus Infection,” Journal of Virology 76, no. 24 (2002): 12584–12595. 12438584 10.1128/JVI.76.24.12584-12595.2002 PMC136690

[180] T. Tu and M. W. Douglas , “Hepatitis B Virus Infection: From Diagnostics to Treatments,” Viruses. 12, no. 12 (2020): 1366. 33265922 10.3390/v12121366 PMC7761508

[181] C. Mehrfeld , S. Zenner , M. Kornek , et al., “The Contribution of Non‐professional Antigen‐presenting Cells to Immunity and Tolerance in the Liver,” Frontiers in Immunology 9 (2018): 635. 29643856 10.3389/fimmu.2018.00635 PMC5882789

[182] D. Loureiro , I. Tout , S. Narguet , et al., “Mitochondrial Stress in Advanced Fibrosis and Cirrhosis Associated With Chronic hepatitis B, Chronic hepatitis C, or Nonalcoholic Steatohepatitis,” Hepatology 77, no. 4 (2023): 1348–1365. 35971873 10.1002/hep.32731 PMC10026976

[183] N. Nasser , P. Tonnerre , A. Mansouri , et al., “Hepatitis‐B Virus: Replication Cycle, Targets, and Antiviral Approaches,” Current Opinion in Virology 63 (2023): 101360. 37696687 10.1016/j.coviro.2023.101360

[184] R. Lozano , M. Naghavi , K. Foreman , et al., “Global and Regional Mortality From 235 Causes of Death for 20 Age Groups in 1990 and 2010: A Systematic Analysis for the Global Burden of Disease Study 2010,” The Lancet 380, no. 9859 (2012): 2095–2128. 10.1016/S0140-6736(12)61728-0 PMC10790329 23245604

[185] J. Rehm , A. V. Samokhvalov , and K. D. Shield , “Global Burden of Alcoholic Liver Diseases,” Journal of Hepatology 59, no. 1 (2013): 160–168. 23511777 10.1016/j.jhep.2013.03.007

[186] A. K. Singal , R. Bataller , J. Ahn , et al., “ACG Clinical Guideline: Alcoholic Liver Disease,” American Journal of Gastroenterology 113, no. 2 (2018): 175–194. 29336434 10.1038/ajg.2017.469 PMC6524956

[187] H. K. Seitz , R. Bataller , H. Cortez‐Pinto , et al., “Alcoholic Liver Disease,” Nature Reviews Disease Primers 4, no. 1 (2018): 16. 10.1038/s41572-018-0014-7 30115921

[188] B. P. Lee , E. Vittinghoff , J. L. Dodge , et al., “National Trends and Long‐term Outcomes of Liver Transplant for Alcohol‐Associated Liver Disease in the United States,” JAMA Internal Medicine 179, no. 3 (2019): 340–348. 30667468 10.1001/jamainternmed.2018.6536 PMC6439700

[189] N. Hosseini , J. Shor , and G. Szabo , “Alcoholic hepatitis: A Review,” Alcohol and Alcoholism 54, no. 4 (2019): 408–416. 31219169 10.1093/alcalc/agz036 PMC6671387

[190] P. Kasper , S. Lang , H. Steffen , et al., “Management of Alcoholic hepatitis: A Clinical Perspective,” Liver International 43, no. 10 (2023): 2078–2095. 37605624 10.1111/liv.15701

[191] M. R. Thursz , P. Richardson , M. Allison , et al., “Prednisolone or Pentoxifylline for Alcoholic hepatitis,” New England Journal of Medicine 372, no. 17 (2015): 1619–1628. 25901427 10.1056/NEJMoa1412278

[192] B. Gao , Y. Duan , S. Lang , et al., “Functional Microbiomics Reveals Alterations of the Gut Microbiome and Host co‐metabolism in Patients With Alcoholic hepatitis,” Hepatology Communications 4, no. 8 (2020): 1168–1182. 32766476 10.1002/hep4.1537 PMC7395072

[193] L. Jiang , S. Lang , Y. Duan , et al., “Intestinal Virome in Patients With Alcoholic hepatitis,” Hepatology 72, no. 6 (2020): 2182–2196. 32654263 10.1002/hep.31459 PMC8159727

[194] S. Lang , Y. Duan , J. Liu , et al., “Intestinal Fungal Dysbiosis and Systemic Immune Response to Fungi in Patients With Alcoholic hepatitis,” Hepatology 71, no. 2 (2020): 522–538. 31228214 10.1002/hep.30832 PMC6925657

[195] S. Lang , B. Fairfied , B. Gao , et al., “Changes in the Fecal Bacterial Microbiota Associated With Disease Severity in Alcoholic hepatitis Patients,” Gut Microbes 12, no. 1 (2020): 1785251. 32684075 10.1080/19490976.2020.1785251 PMC7524371

[196] H. Chu , Y. Duan , S. Lang , et al., “The Candida albicans Exotoxin Candidalysin Promotes Alcohol‐associated Liver Disease,” Journal of Hepatology 72, no. 3 (2020): 391–400. 31606552 10.1016/j.jhep.2019.09.029 PMC7031049

[197] W. Zhong , X. Wei , L. Hao , et al., “Paneth Cell Dysfunction Mediates Alcohol‐related Steatohepatitis Through Promoting Bacterial Translocation in Mice: Role of Zinc Deficiency,” Hepatology 71, no. 5 (2020): 1575–1591. 31520476 10.1002/hep.30945 PMC7069794

[198] P. Stärkel and B. Schnabl , “Bidirectional Communication Between Liver and Gut During Alcoholic Liver Disease,” Seminars in Liver Disease 36, no. 4 (2016): 331–339. 27997973 10.1055/s-0036-1593882

[199] S. Lang and B. Schnabl , “Microbiota and Fatty Liver Disease‐the Known, the Unknown, and the Future,” Cell Host & Microbe 28, no. 2 (2020): 233–244. 32791115 10.1016/j.chom.2020.07.007 PMC7467841

[200] Y. Duan , H. Chu , K. Brandl , et al., “CRIg on Liver Macrophages Clears Pathobionts and Protects Against Alcoholic Liver Disease,” Nature Communications 12, no. 1 (2021): 7172. 10.1038/s41467-021-27385-3 PMC8660815 34887405

[201] A. K. Singal and P. Mathurin , “Diagnosis and Treatment of Alcohol‐associated Liver Disease: A Review,” Jama 326, no. 2 (2021): 165–176. 34255003 10.1001/jama.2021.7683

[202] R. Bataller , J. P. Arab , and V. H. Shah , “Alcohol‐associated hepatitis,” New England Journal of Medicine 387, no. 26 (2022): 2436–2448. 36577100 10.1056/NEJMra2207599

[203] M. E. Rinella , J. V. Lazarus , V. Ratziu , et al., “A Multisociety Delphi Consensus Statement on New Fatty Liver Disease Nomenclature,” Hepatology 78, no. 6 (2023): 1966–1986. 37363821 10.1097/HEP.0000000000000520 PMC10653297

[204] T. Huby and E. L. Gautier , “Immune Cell‐mediated Features of Non‐alcoholic Steatohepatitis,” Nature Reviews Immunology 22, no. 7 (2022): 429–443. 10.1038/s41577-021-00639-3 PMC8570243 34741169

[205] H. Hagström , P. Nasr , M. Ekstedt , et al., “Fibrosis Stage but Not NASH Predicts Mortality and Time to Development of Severe Liver Disease in Biopsy‐proven NAFLD,” Journal of Hepatology 67, no. 6 (2017): 1265–1273. 28803953 10.1016/j.jhep.2017.07.027

[206] A. J. Sanyal , M. L. Van Natta , J. Clark , et al., “Prospective Study of Outcomes in Adults With Nonalcoholic Fatty Liver Disease,” New England Journal of Medicine 385, no. 17 (2021): 1559–1569. 34670043 10.1056/NEJMoa2029349 PMC8881985

[207] S. Khanmohammadi , B. Ramos‐Molina , and M. S. Kuchay , “NOD‐Like Receptors in the Pathogenesis of Metabolic (dysfunction)‐associated Fatty Liver Disease: Therapeutic Agents Targeting NOD‐Like Receptors,” Diabetology & Metabolic Syndrome 17, no. 7 (2023): 102788. 10.1016/j.dsx.2023.102788 37302383

[208] L. Yu , F. Gao , Y. Li , et al., “Role of Pattern Recognition Receptors in the Development of MASLD and Potential Therapeutic Applications,” Biomed Pharmacother Biomedecine Pharmacother 175 (2024): 116724. 10.1016/j.biopha.2024.116724 38761424

[209] O. Krenkel and F. Tacke , “Liver Macrophages in Tissue Homeostasis and Disease,” Nature Reviews Immunology 17, no. 5 (2017): 306–321. 10.1038/nri.2017.11 28317925

[210] K. Kazankov , S. M. D. Jørgensen , K. L. Thomsen , et al., “The Role of Macrophages in Nonalcoholic Fatty Liver Disease and Nonalcoholic Steatohepatitis,” Nature reviews Gastroenterology & hepatology 16, no. 3 (2019): 145–159. 30482910 10.1038/s41575-018-0082-x

[211] J. S. Duffield , S. J. Forbes , C. M. Constandinou , et al., “Selective Depletion of Macrophages Reveals Distinct, Opposing Roles During Liver Injury and Repair,” Journal of Clinical Investigation 115, no. 1 (2005): 56–65. 15630444 10.1172/JCI22675 PMC539199

[212] P. Ramachandran , R. Dobie , J. R. Wilson‐Kanamori , et al., “Resolving the Fibrotic Niche of human Liver Cirrhosis at Single‐cell Level,” Nature 575, no. 7783 (2019): 512–518. 31597160 10.1038/s41586-019-1631-3 PMC6876711

[213] M. Parola and M. Pinzani , “Liver Fibrosis in NAFLD/NASH: From Pathophysiology towards Diagnostic and Therapeutic Strategies,” Molecular Aspects of Medicine 95 (2024): 101231. 38056058 10.1016/j.mam.2023.101231

[214] A. Iwata , J. Maruyama , S. Natsuki , et al., “Egr2 drives the Differentiation of Ly6Chi Monocytes Into Fibrosis‐promoting Macrophages in Metabolic Dysfunction‐associated Steatohepatitis in Mice,” Communications Biology 7, no. 1 (2024): 681. 38831027 10.1038/s42003-024-06357-5 PMC11148031

[215] Q. Wang , H. Zhou , Q. Bu , et al., “Role of XBP1 in Regulating the Progression of Non‐alcoholic Steatohepatitis,” Journal of Hepatology 77, no. 2 (2022): 312–325. 35292349 10.1016/j.jhep.2022.02.031

[216] T. Lan , F. Gao , Y. Cai , et al., “The Protein circPETH‐147aa Regulates Metabolic Reprogramming in Hepatocellular Carcinoma Cells to Remodel Immunosuppressive Microenvironment,” Nature Communications 16, no. 1 (2025): 333. 10.1038/s41467-024-55577-0 PMC11696079 39747873

[217] H. Guo , M. Wang , C. Ni , et al., “TREM2 promotes the Formation of a Tumor‐supportive Microenvironment in Hepatocellular Carcinoma,” Journal of Experimental & Clinical Cancer Research 44, no. 1 (2025): 20. 39838454 10.1186/s13046-025-03287-w PMC11748316

[218] Z.‐Z. Bai , H.‐Y. Li , C.‐H. Li , et al., “Retraction Note: M1 Macrophage‐derived Exosomal MicroRNA‐326 Suppresses Hepatocellular Carcinoma Cell Progression via Mediating NF‐κB Signaling Pathway,” Nanoscale Research Letters 17, no. 1 (2022): 122. 36512188 10.1186/s11671-022-03763-8 PMC9748008

[219] J. Zhao , H. Li , S. Zhao , et al., “Epigenetic Silencing of miR‐144/451a Cluster Contributes to HCC Progression via Paracrine HGF/MIF‐mediated TAM Remodeling,” Molecular cancer 20, no. 1 (2021): 46. 33658044 10.1186/s12943-021-01343-5 PMC7927270

[220] L. Wang , Y.‐Y. Hu , J.‐L. Zhao , et al., “Targeted Delivery of miR‐99b Reprograms Tumor‐associated Macrophage Phenotype Leading to Tumor Regression,” Journal for ImmunoTherapy of Cancer 8, no. 2 (2020): e000517. 32948650 10.1136/jitc-2019-000517 PMC7511616

[221] Y.‐L. Zhang , Q. Li , X.‐M. Yang , et al., “SPON2 promotes M1‐Like Macrophage Recruitment and Inhibits Hepatocellular Carcinoma Metastasis by Distinct Integrin‐Rho GTPase‐Hippo Pathways,” Cancer Research 78, no. 9 (2018): 2305–2317. 29440144 10.1158/0008-5472.CAN-17-2867

[222] B. Zhou , Y. Yang , and C. Li , “SIRT1 inhibits Hepatocellular Carcinoma Metastasis by Promoting M1 Macrophage Polarization via NF‐κB Pathway,” OncoTargets and Therapy 12 (2019): 2519–2529. 31040695 10.2147/OTT.S195234 PMC6452816

[223] B. Zhou , C. Li , Y. Yang , et al., “RIG‐I Promotes Cell Death in Hepatocellular Carcinoma by Inducing M1 Polarization of Perineal Macrophages Through the RIG‐I/MAVS/NF‐κB Pathway,” OncoTargets and Therapy 13 (2020): 8783–8794. 32982277 10.2147/OTT.S258450 PMC7493023

[224] Q. Wang , F. Cheng , T.‐T. Ma , et al., “Interleukin‐12 Inhibits the Hepatocellular Carcinoma Growth by Inducing Macrophage Polarization to the M1‐Like Phenotype Through Downregulation of Stat‐3,” Molecular and Cellular Biochemistry 415, no. 1–2 (2016): 157–168. 27003285 10.1007/s11010-016-2687-0

[225] O. W. H. Yeung , C.‐M. Lo , C.‐C. Ling , et al., “Alternatively Activated (M2) Macrophages Promote Tumour Growth and Invasiveness in Hepatocellular Carcinoma,” Journal of Hepatology 62, no. 3 (2015): 607–616. 25450711 10.1016/j.jhep.2014.10.029

[226] F. Zhu , X. Li , S. Chen , et al., “Tumor‐associated Macrophage or Chemokine Ligand CCL17 Positively Regulates the Tumorigenesis of Hepatocellular Carcinoma,” Medical Oncology 33, no. 2 (2016): 17. 26781124 10.1007/s12032-016-0729-9

[227] D. Sun , T. Luo , P. Dong , et al., “M2‐polarized Tumor‐associated Macrophages Promote Epithelial‐mesenchymal Transition via Activation of the AKT3/PRAS40 Signaling Pathway in Intrahepatic Cholangiocarcinoma,” Journal of Cellular Biochemistry 121, no. 4 (2020): 2828–2838. 31692069 10.1002/jcb.29514

[228] H. Yuan , Z. Lin , Y. Liu , et al., “Intrahepatic Cholangiocarcinoma Induced M2‐polarized Tumor‐associated Macrophages Facilitate Tumor Growth and Invasiveness,” Cancer cell international 20, no. 1 (2020): 586. 33372604 10.1186/s12935-020-01687-w PMC7720384

[229] C. Subimerb , S. Pinlaor , V. Lulitanond , et al., “Circulating CD14(+) CD16(+) Monocyte Levels Predict Tissue Invasive Character of Cholangiocarcinoma,” Clinical and Experimental Immunology 161, no. 3 (2010): 471–479. 20636398 10.1111/j.1365-2249.2010.04200.x PMC2962964

[230] H. Hasita , Y. Komohara , H. Okabe , et al., “Significance of Alternatively Activated Macrophages in Patients With Intrahepatic Cholangiocarcinoma,” Cancer Science 101, no. 8 (2010): 1913–1919. 20545696 10.1111/j.1349-7006.2010.01614.x PMC11158749

[231] Y. Zhang , C. Yan , Y. Dong , et al., “ANGPTL3 accelerates Atherosclerotic Progression via Direct Regulation of M1 Macrophage Activation in Plaque,” Journal of Advanced Research (2024). S2090‐1232(24)00201–7. 10.1016/j.jare.2024.05.011 PMC11976407 38740260

[232] A. Das , F. Reis , and P. K. Mishra , “mTOR Signaling in Cardiometabolic Disease, Cancer, and Aging 2018,” Oxidative Medicine and Cellular Longevity 2019 (2019): 9692528. 30863483 10.1155/2019/9692528 PMC6378800

[233] D. Ai , H. Jiang , M. Westerterp , et al., “Disruption of Mammalian Target of Rapamycin Complex 1 in Macrophages Decreases Chemokine Gene Expression and Atherosclerosis,” Circulation Research 114, no. 10 (2014): 1576–1584. 24687132 10.1161/CIRCRESAHA.114.302313 PMC4058053

[234] X. Zhang , I. Sergin , T. D. Evans , et al., “High‐protein Diets Increase Cardiovascular Risk by Activating Macrophage mTOR to Suppress Mitophagy,” Nature Metabolism 2, no. 1 (2020): 110–125. 10.1038/s42255-019-0162-4 PMC7053091 32128508

[235] X. Zhang , J. G. McDonald , B. Aryal , et al., “Desmosterol Suppresses Macrophage Inflammasome Activation and Protects Against Vascular Inflammation and Atherosclerosis,” PNAS 118, no. 47 (2021): e2107682118. 34782454 10.1073/pnas.2107682118 PMC8617522

[236] A. Canfrán‐Duque , N. Rotllan , X. Zhang , et al., “Macrophage‐derived 25‐hydroxycholesterol Promotes Vascular Inflammation, Atherogenesis, and Lesion Remodeling,” Circulation 147, no. 5 (2023): 388–408. 36416142 10.1161/CIRCULATIONAHA.122.059062 PMC9892282

[237] H. Wang , W. Mehal , L. E. Nagy , et al., “Immunological Mechanisms and Therapeutic Targets of Fatty Liver Diseases,” Cellular & Molecular Immunology 18, no. 1 (2021): 73–91. 33268887 10.1038/s41423-020-00579-3 PMC7852578

[238] Z. Du , Y. Wang , J. Liang , et al., “Association of Glioma CD44 Expression With Glial Dynamics in the Tumour Microenvironment and Patient Prognosis,” Computational and Structural Biotechnology Journal 20 (2022): 5203–5217. 36187921 10.1016/j.csbj.2022.09.003 PMC9508470

[239] C. Baeck , A. Wehr , K. R. Karlmark , et al., “Pharmacological Inhibition of the Chemokine CCL2 (MCP‐1) Diminishes Liver Macrophage Infiltration and Steatohepatitis in Chronic Hepatic Injury,” Gut 61, no. 3 (2012): 416–426. 21813474 10.1136/gutjnl-2011-300304

[240] M. Baba , H. Miyake , X. Wang , et al., “Isolation and Characterization of human Immunodeficiency Virus Type 1 Resistant to the Small‐molecule CCR5 Antagonist TAK‐652,” Antimicrobial Agents and Chemotherapy 51, no. 2 (2007): 707–715. 17116673 10.1128/AAC.01079-06 PMC1797735

[241] A. Ambade , P. Lowe , K. Kodys , et al., “Pharmacological Inhibition of CCR2/5 Signaling Prevents and Reverses Alcohol‐Induced Liver Damage, Steatosis, and Inflammation in Mice,” Hepatology (Baltimore, Md.) 69, no. 3 (2019): 1105–1121. 10.1002/hep.30249 PMC6393202 30179264

[242] O. Krenkel , T. Puengel , O. Govaere , et al., “Therapeutic Inhibition of Inflammatory Monocyte Recruitment Reduces Steatohepatitis and Liver Fibrosis,” Hepatology (Baltimore, Md.) 67, no. 4 (2018): 1270–1283. 10.1002/hep.29544 28940700

[243] Y. Guo , C. Zhao , W. Dai , et al., “C‐C Motif Chemokine Receptor 2 Inhibition Reduces Liver Fibrosis by Restoring the Immune Cell Landscape,” International Journal of Biological Sciences 19, no. 8 (2023): 2572–2587. 37215993 10.7150/ijbs.83530 PMC10197881

[244] Q. M. Anstee , B. A. Neuschwander‐Tetri , V. Wai‐Sun Wong , et al., “Cenicriviroc Lacked Efficacy to Treat Liver Fibrosis in Nonalcoholic Steatohepatitis: AURORA Phase III Randomized Study,” Clin Gastroenterol Hepatol Off Clin Pract J Am Gastroenterol Assoc 22, no. 1 (2024): 124–134. e1. 10.1016/j.cgh.2023.04.003 37061109

[245] L. Fan , R. Lai , N. Ma , et al., “miR‐552‐3p Modulates Transcriptional Activities of FXR and LXR to Ameliorate Hepatic Glycolipid Metabolism Disorder,” Journal of Hepatology 74, no. 1 (2021): 8–19. 32818571 10.1016/j.jhep.2020.07.048

[246] L. Parlati , M. Régnier , H. Guillou , et al., “New Targets for NAFLD,” JHEP Reports: Innovation in Hepatology 3, no. 6 (2021): 100346. 34667947 10.1016/j.jhepr.2021.100346 PMC8507191

[247] R. Liu , M. Scimeca , Q. Sun , et al., “Harnessing Metabolism of Hepatic Macrophages to Aid Liver Regeneration,” Cell Death & Disease 14, no. 8 (2023): 574. 37644019 10.1038/s41419-023-06066-7 PMC10465526

[248] X. Han , Y. Wu , Q. Yang , et al., “Peroxisome Proliferator‐activated Receptors in the Pathogenesis and Therapies of Liver Fibrosis,” Pharmacology & Therapeutics 222 (2021): 107791. 33321113 10.1016/j.pharmthera.2020.107791

[249] A. Nikam , J. V. Patankar , M. Somlapura , et al., “The PPARα Agonist Fenofibrate Prevents Formation of Protein Aggregates (Mallory‐Denk bodies) in a Murine Model of Steatohepatitis‐Like Hepatotoxicity,” Scientific Reports 8, no. 1 (2018): 12964. 30154499 10.1038/s41598-018-31389-3 PMC6113278

[250] B. Staels , A. Rubenstrunk , B. Noel , et al., “Hepatoprotective Effects of the Dual Peroxisome Proliferator‐activated Receptor Alpha/Delta Agonist, GFT505, in Rodent Models of Nonalcoholic Fatty Liver Disease/Nonalcoholic Steatohepatitis,” Hepatology (Baltimore, Md.) 58, no. 6 (2013): 1941–1952. 10.1002/hep.26461 23703580

[251] W. Deng , Z. Meng , A. Sun , et al., “Pioglitazone Suppresses Inflammation and Fibrosis in Nonalcoholic Fatty Liver Disease by Down‐regulating PDGF and TIMP‐2: Evidence From in Vitro Study,” Cancer Biomarkers: Section A of Disease Markers 20, no. 4 (2017): 411–415. 10.3233/CBM-170157 28946547

[252] D. Shen , H. Li , R. Zhou , et al., “Pioglitazone Attenuates Aging‐related Disorders in Aged Apolipoprotein E Deficient Mice,” Experimental Gerontology 102 (2018): 101–108. 29221940 10.1016/j.exger.2017.12.002

[253] V. Ratziu , S. A. Harrison , S. Francque , et al., “Elafibranor, an Agonist of the Peroxisome Proliferator‐Activated Receptor‐α and ‐δ, Induces Resolution of Nonalcoholic Steatohepatitis without Fibrosis Worsening,” Gastroenterology 150, no. 5 (2016): 1147–1159. e5. 26874076 10.1053/j.gastro.2016.01.038

[254] K. Iwaisako , M. Haimerl , Y.‐H. Paik , et al., “Protection From Liver Fibrosis by a Peroxisome Proliferator‐activated Receptor δ Agonist,” PNAS 109, no. 21 (2012): E1369–1376. 22538808 10.1073/pnas.1202464109 PMC3361396

[255] H.‐J. Lim , J.‐H. Park , S. Lee , et al., “PPARdelta Ligand L‐165041 Ameliorates Western Diet‐induced Hepatic Lipid Accumulation and Inflammation in LDLR‐/‐ mice,” European Journal of Pharmacology 622, no. 1–3 (2009): 45–51. 19766624 10.1016/j.ejphar.2009.09.002

[256] A. D. Patterson , Y. M. Shah , T. Matsubara , et al., “Peroxisome Proliferator‐activated Receptor Alpha Induction of Uncoupling Protein 2 Protects Against Acetaminophen‐induced Liver Toxicity,” Hepatology (Baltimore, Md.) 56, no. 1 (2012): 281–290. 10.1002/hep.25645 PMC3378765 22318764

[257] N. C. Teoh , J. Williams , J. Hartley , et al., “Short‐term Therapy With Peroxisome Proliferation‐activator Receptor‐alpha Agonist Wy‐14,643 Protects Murine Fatty Liver Against Ischemia‐reperfusion Injury,” Hepatology (Baltimore, Md.) 51, no. 3 (2010): 996–1006. 10.1002/hep.23420 20131406

[258] V. Ratziu , S. Francque , and A. Sanyal , “Breakthroughs in Therapies for NASH and Remaining Challenges,” Journal of Hepatology 76, no. 6 (2022): 1263–1278. 35589249 10.1016/j.jhep.2022.04.002

[259] A. M. Majzoub , T. Nayfeh , A. Barnard , et al., “Systematic Review With Network Meta‐analysis: Comparative Efficacy of Pharmacologic Therapies for Fibrosis Improvement and Resolution of NASH,” Alimentary pharmacology & therapeutics 54, no. 7 (2021): 880–889. 34435378 10.1111/apt.16583 PMC8711247

[260] T. L. Laursen , A. Mellemkjær , H. J. Møller , et al., “Spotlight on Liver Macrophages for Halting Injury and Progression in Nonalcoholic Fatty Liver Disease,” Expert Opinion on Therapeutic Targets 26, no. 8 (2022): 697–705. 36205054 10.1080/14728222.2022.2132145

[261] T. Karagiannis , I. Avgerinos , A. Liakos , et al., “Management of Type 2 Diabetes With the Dual GIP/GLP‐1 Receptor Agonist Tirzepatide: A Systematic Review and Meta‐analysis,” Diabetologia 65, no. 8 (2022): 1251–1261. 35579691 10.1007/s00125-022-05715-4 PMC9112245

[262] A. Mantovani , C. D. Byrne , and G. Targher , “Efficacy of Peroxisome Proliferator‐activated Receptor Agonists, Glucagon‐Like Peptide‐1 Receptor Agonists, or Sodium‐glucose Cotransporter‐2 Inhibitors for Treatment of Non‐alcoholic Fatty Liver Disease: A Systematic Review,” The Lancet Gastroenterology and Hepatology 7, no. 4 (2022): 367–378. 35030323 10.1016/S2468-1253(21)00261-2

[263] J. M. Yabut and D. J. Drucker Endocrine Reviews 44, no. 1 (2023): 14–32. 35907261 10.1210/endrev/bnac018

[264] G. Targher , “Tirzepatide Adds Hepatoprotection to Its Armoury,” The Lancet Diabetes & Endocrinology 10, no. 6 (2022): 374–375. 35468320 10.1016/S2213-8587(22)00074-2

[265] J. M. Yabut and D. J. Drucker , “Glucagon‐Like Peptide‐1 Receptor‐based Therapeutics for Metabolic Liver Disease,” Endocrine Reviews 44, no. 1 (2023): 14–32. 35907261 10.1210/endrev/bnac018

[266] N. Song , H. Xu , J. Liu , et al., “Design of a Highly Potent GLP‐1R and GCGR Dual‐agonist for Recovering Hepatic Fibrosis,” Acta Pharmaceutica Sinica B 12, no. 5 (2022): 2443–2461. 35646543 10.1016/j.apsb.2021.12.016 PMC9136578

[267] Y. Peng , H. Lin , S. Tian , et al., “Glucagon‐Like Peptide‐1 Receptor Activation Maintains Extracellular Matrix Integrity by Inhibiting the Activity of Mitogen‐activated Protein Kinases and Activator Protein‐1,” Free Radical Biology and Medicine 177 (2021): 247–259. 34737144 10.1016/j.freeradbiomed.2021.10.034

[268] R. Bruen , S. Curley , S. Kajani , et al., “Liraglutide dictates macrophage phenotype in apolipoprotein E null mice during early atherosclerosis,” Cardiovascular Diabetology 16, no. 1 (2017). 10.1186/s12933-017-0626-3. PMC5674826 29110715

[269] Á. Vinué , J. Navarro , A. Herrero‐Cervera , et al., “The GLP‐1 Analogue Lixisenatide Decreases Atherosclerosis in Insulin‐resistant Mice by Modulating Macrophage Phenotype,” Diabetologia 60, no. 9 (2017): 1801–1812. 28608285 10.1007/s00125-017-4330-3

[270] P. N. Newsome and P. Ambery , “Incretins (GLP‐1 receptor agonists and dual/triple agonists) and the Liver,” Journal of Hepatology 79, no. 6 (2023): 1557–1565. 37562748 10.1016/j.jhep.2023.07.033

[271] R. Nevola , R. Epifani , S. Imbriani , et al., “GLP‐1 Receptor Agonists in Non‐Alcoholic Fatty Liver Disease: Current Evidence and Future Perspectives,” International Journal of Molecular Sciences 24, no. 2 (2023): 1703. 36675217 10.3390/ijms24021703 PMC9865319

[272] F. Yang , X. Luo , J. Li , et al., “Application of Glucagon‐Like Peptide‐1 Receptor Antagonists in Fibrotic Diseases,” Biomed Pharmacother Biomedecine Pharmacother 152 (2022): 113236. 10.1016/j.biopha.2022.113236 35691154

[273] J. H. D. Bassett and G. R. Williams , “The Molecular Actions of Thyroid Hormone in Bone,” Trends in Endocrinology & Metabolism 14, no. 8 (2003): 356–364. 14516933 10.1016/s1043-2760(03)00144-9

[274] S. A. Harrison , M. R. Bashir , C. D. Guy , et al., “Resmetirom (MGL‐3196) for the Treatment of Non‐alcoholic Steatohepatitis: A Multicentre, Randomised, Double‐blind, Placebo‐controlled, Phase 2 Trial,” Lancet Lond Engl 394, no. 10213 (2019): 2012–2024. 10.1016/S0140-6736(19)32517-6 31727409

[275] T. Puengel , H. Liu , A. Guillot , et al., “Nuclear Receptors Linking Metabolism, Inflammation, and Fibrosis in Nonalcoholic Fatty Liver Disease,” International Journal of Molecular Sciences 23, no. 5 (2022): 2668. 35269812 10.3390/ijms23052668 PMC8910763

[276] X. Wang , L. Wang , L. Geng , et al., “Resmetirom Ameliorates NASH‐Model Mice by Suppressing STAT3 and NF‐κB Signaling Pathways in an RGS5‐Dependent Manner,” International Journal of Molecular Sciences 24, no. 6 (2023): 5843. 36982915 10.3390/ijms24065843 PMC10058113

[277] S. A. Kliewer and D. J. Mangelsdorf , “Fibroblast Growth Factor 21: From Pharmacology to Physiology,” American Journal of Clinical Nutrition 91, no. 1 (2010): 254S–257S. 19906798 10.3945/ajcn.2009.28449B PMC2793111

[278] Y. Liu , C. Zhao , J. Xiao , et al., “Fibroblast Growth Factor 21 Deficiency Exacerbates Chronic Alcohol‐induced Hepatic Steatosis and Injury,” Scientific Reports 6 (2016): 31026. 27498701 10.1038/srep31026 PMC4976373

[279] R. Loomba , A. J. Sanyal , K. V. Kowdley , et al., “Randomized, Controlled Trial of the FGF21 Analogue Pegozafermin in NASH,” New England Journal of Medicine 389, no. 11 (2023): 998–1008. 37356033 10.1056/NEJMoa2304286 PMC10718287

[280] K. H. Flippo , S. A. J. Trammell , M. P. Gillum , et al., “FGF21 suppresses Alcohol Consumption Through an Amygdalo‐striatal Circuit,” Cell metabolism 34, no. 2 (2022): 317–328. e6. 35108517 10.1016/j.cmet.2021.12.024 PMC9093612

[281] C. Liu , M. Schönke , B. Spoorenberg , et al., “FGF21 protects Against Hepatic Lipotoxicity and Macrophage Activation to Attenuate Fibrogenesis in Nonalcoholic Steatohepatitis,” Elife 12 (2023): e83075. 36648330 10.7554/eLife.83075 PMC9928421

[282] M. Cariello and A. Moschetta , “Fibroblast Growth Factor 21: A New Liver Safeguard,” Hepatology (Baltimore, Md.) 60, no. 3 (2014): 792–794. 10.1002/hep.27147 24668840

[283] N. Tanaka , S. Takahashi , Y. Zhang , et al., “Role of Fibroblast Growth Factor 21 in the Early Stage of NASH Induced by Methionine‐ and Choline‐deficient Diet,” Biochimica Et Biophysica Acta 1852, no. 7 (2015): 1242–1252. 25736301 10.1016/j.bbadis.2015.02.012 PMC4433820

[284] F. M. Fisher , P. C. Chui , I. A. Nasser , et al., “Fibroblast Growth Factor 21 Limits Lipotoxicity by Promoting Hepatic Fatty Acid Activation in Mice on Methionine and Choline‐deficient Diets,” Gastroenterology 147, no. 5 (2014): 1073–1083. e6. 25083607 10.1053/j.gastro.2014.07.044 PMC4570569

[285] X. Yang , Z. Jin , D. Lin , et al., “FGF21 alleviates Acute Liver Injury by Inducing the SIRT1‐autophagy Signalling Pathway,” Journal of Cellular and Molecular Medicine 26, no. 3 (2022): 868–879. 34984826 10.1111/jcmm.17144 PMC8817117

[286] X. Wan , X. Lu , Y. Xiao , et al., “ATF4‐ and CHOP‐dependent Induction of FGF21 Through Endoplasmic Reticulum Stress,” BioMed research international 2014 (2014): 807874. 24900988 10.1155/2014/807874 PMC4037570

[287] S. Jiang , C. Yan , Q. Fang , et al., “Fibroblast Growth Factor 21 Is Regulated by the IRE1α‐XBP1 Branch of the Unfolded Protein Response and Counteracts Endoplasmic Reticulum Stress‐induced Hepatic Steatosis,” Journal of Biological Chemistry 289, no. 43 (2014): 29751–29765. 25170079 10.1074/jbc.M114.565960 PMC4207989

[288] L. Wu , Q. Pan , G. Wu , et al., “Diverse Changes of Circulating Fibroblast Growth Factor 21 Levels in Hepatitis B Virus‐Related Diseases,” Scientific Reports 7, no. 1 (2017): 16482. 29184085 10.1038/s41598-017-16312-6 PMC5705770

[289] D. Ye , Y. Wang , H. Li , et al., “Fibroblast Growth Factor 21 Protects Against Acetaminophen‐induced Hepatotoxicity by Potentiating Peroxisome Proliferator‐activated Receptor Coactivator Protein‐1α‐mediated Antioxidant Capacity in Mice,” Hepatology (Baltimore, Md.) 60, no. 3 (2014): 977–989. 10.1002/hep.27060 24590984

[290] D. Ye , H. Li , Y. Wang , et al., “Circulating Fibroblast Growth Factor 21 Is a Sensitive Biomarker for Severe Ischemia/Reperfusion Injury in Patients With Liver Transplantation,” Scientific Reports 6 (2016): 19776. 26806156 10.1038/srep19776 PMC4726235

[291] R. Loomba , E. J. Lawitz , J. P. Frias , et al., “Safety, Pharmacokinetics, and Pharmacodynamics of pegozafermin in Patients With Non‐alcoholic Steatohepatitis: A Randomised, Double‐blind, Placebo‐controlled, Phase 1b/2a Multiple‐ascending‐dose Study,” The Lancet Gastroenterology and Hepatology 8, no. 2 (2023): 120–132. 36521501 10.1016/S2468-1253(22)00347-8

[292] A. C. Mackinnon , D. Tonev , B. Jacoby , et al., “Galectin‐3: Therapeutic Targeting in Liver Disease,” Expert Opinion on Therapeutic Targets 27, no. 9 (2023): 779–791. 37705214 10.1080/14728222.2023.2258280

[293] H. Liu , L. Zhang , Z. Liu , et al., “Galectin‐3 as TREM2 Upstream Factor Contributes to Lung Ischemia‐reperfusion Injury by Regulating Macrophage Polarization,” Iscience 26, no. 9 (2023): 107496. 37636061 10.1016/j.isci.2023.107496 PMC10448077

[294] H. Li , Z. Cao , L. Wang , et al., “Chronic High‐fat Diet Induces Galectin‐3 and TLR4 to Activate NLRP3 Inflammasome in NASH,” Journal of Nutritional Biochemistry 112 (2023): 109217. 36402251 10.1016/j.jnutbio.2022.109217

[295] S. A. de Oliveira , B. S. de Freitas Souza , E. P. Sá Barreto , et al., “Reduction of Galectin‐3 Expression and Liver Fibrosis After Cell Therapy in a Mouse Model of Cirrhosis,” Cytotherapy 14, no. 3 (2012): 339–349. 22149185 10.3109/14653249.2011.637668

[296] L. Li , J. Li , and J. Gao , “Functions of Galectin‐3 and Its Role in Fibrotic Diseases,” Journal of Pharmacology and Experimental Therapeutics 351, no. 2 (2014): 336–343. 25194021 10.1124/jpet.114.218370

[297] N. C. Henderson , A. C. Mackinnon , S. L. Farnworth , et al., “Galectin‐3 Regulates Myofibroblast Activation and Hepatic Fibrosis,” PNAS 103, no. 13 (2006): 5060–5065. 16549783 10.1073/pnas.0511167103 PMC1458794

[298] S. A. Harrison , A. Dennis , M. M. Fiore , et al., “Utility and Variability of Three Non‐invasive Liver Fibrosis Imaging Modalities to Evaluate Efficacy of GR‐MD‐02 in Subjects With NASH and Bridging Fibrosis During a Phase‐2 Randomized Clinical Trial,” PLoS ONE 13, no. 9 (2018): e0203054. 30192782 10.1371/journal.pone.0203054 PMC6128474

[299] A. C. MacKinnon , S. L. Farnworth , P. S. Hodkinson , et al., “Regulation of Alternative Macrophage Activation by Galectin‐3,” Journal of Immunology (Baltimore, Md.: 1950) 180, no. 4 (2008): 2650–2658. 18250477 10.4049/jimmunol.180.4.2650

[300] A. Arsenijevic , J. Milovanovic , B. Stojanovic , et al., “Gal‐3 Deficiency Suppresses Novosphyngobium Aromaticivorans Inflammasome Activation and IL‐17 Driven Autoimmune Cholangitis in Mice,” Frontiers in Immunology 10 (2019): 1309. 31231399 10.3389/fimmu.2019.01309 PMC6568238

[301] B. Simovic Markovic , A. Nikolic , M. Gazdic , et al., “Galectin‐3 Plays an Important Pro‐inflammatory Role in the Induction Phase of Acute Colitis by Promoting Activation of NLRP3 Inflammasome and Production of IL‐1β in Macrophages,” Journal of Crohn's and Colitis 10, no. 5 (2016): 593–606. 10.1093/ecco-jcc/jjw013 PMC4957458 26786981

[302] F. R. Zetterberg , A. MacKinnon , T. Brimert , et al., “Discovery and Optimization of the First Highly Effective and Orally Available Galectin‐3 Inhibitors for Treatment of Fibrotic Disease,” Journal of Medicinal Chemistry 65, no. 19 (2022): 12626–12638. 36154172 10.1021/acs.jmedchem.2c00660 PMC9574852

[303] J. Zhao , Y.‐C. Fan , X.‐Y. Liu , et al., “Hypermethylation of the Galectin‐3 Promoter Is Associated With Poor Prognosis of Acute‐on‐chronic hepatitis B Liver Failure,” Dig Liver Dis Off J Ital Soc Gastroenterol Ital Assoc Study Liver 49, no. 6 (2017): 664–671. 10.1016/j.dld.2017.01.158 28185839

[304] M. Matsuo , A. Kanbe , K. Noguchi , et al., “Time‐course Analysis of Liver and Serum Galectin‐3 in Acute Liver Injury After Alpha‐galactosylceramide Injection,” PLoS ONE 19, no. 2 (2024): e0298284. 38330036 10.1371/journal.pone.0298284 PMC10852258

[305] S. A. Harrison , V. W.‐S. Wong , T. Okanoue , et al., “Selonsertib for Patients With Bridging Fibrosis or Compensated Cirrhosis due to NASH: Results From Randomized Phase III STELLAR Trials,” Journal of Hepatology 73, no. 1 (2020): 26–39. 32147362 10.1016/j.jhep.2020.02.027

[306] T. Lan , Y. Hu , F. Hu , et al., “Hepatocyte Glutathione S‐transferase Mu 2 Prevents Non‐alcoholic Steatohepatitis by Suppressing ASK1 Signaling,” Journal of Hepatology 76, no. 2 (2022): 407–419. 34656650 10.1016/j.jhep.2021.09.040

[307] Y. Jiao , P. Xu , H. Shi , et al., “Advances on Liver Cell‐derived Exosomes in Liver Diseases,” Journal of Cellular and Molecular Medicine 25, no. 1 (2021): 15–26. 33247543 10.1111/jcmm.16123 PMC7810930

[308] T. Liwinski , M. Heinemann , and C. Schramm , “The Intestinal and Biliary Microbiome in Autoimmune Liver Disease‐current Evidence and Concepts,” Seminars in Immunopathology 44, no. 4 (2022): 485–507. 35536431 10.1007/s00281-022-00936-6 PMC9088151

[309] C. A. Mecoli , T. Igusa , M. Chen , et al., “Subsets of Idiopathic Inflammatory Myositis Enriched for Contemporaneous Cancer Relative to the General Population,” Arthritis & Rheumatology (Hoboken, N.J.) 75, no. 4 (2023): 620–629. 35878018 10.1002/art.42311 PMC9873833

[310] Y. H. Lee , H.‐J. Jang , S. Kim , et al., “Hepatic MIR20B Promotes Nonalcoholic Fatty Liver Disease by Suppressing PPARA,” Elife 10 (2021): e70472. 34964438 10.7554/eLife.70472 PMC8758141

[311] X. Zhang , J. Shen , K. Man , et al., “CXCL10 plays a Key Role as an Inflammatory Mediator and a Non‐invasive Biomarker of Non‐alcoholic Steatohepatitis,” Journal of Hepatology 61, no. 6 (2014): 1365–1375. 25048951 10.1016/j.jhep.2014.07.006

[312] L. Grønbæk , H. Vilstrup , and P. Jepsen , “Autoimmune hepatitis in Denmark: Incidence, Prevalence, Prognosis, and Causes of Death. A Nationwide Registry‐based Cohort Study,” Journal of Hepatology 60, no. 3 (2014): 612–617. 24326217 10.1016/j.jhep.2013.10.020

[313] L. Grønbaek , H. Otete , L. Ban , et al., “Incidence, Prevalence and Mortality of Autoimmune hepatitis in England 1997–2015. A Population‐based Cohort Study,” Liver Int Off J Int Assoc Study Liver 40, no. 7 (2020): 1634–1644. 10.1111/liv.14480 32304617

[314] G. J. Webb , R. P. Ryan , T. P. Marshall , et al., “The Epidemiology of UK Autoimmune Liver Disease Varies with Geographic Latitude,” Clin Gastroenterol Hepatol Off Clin Pract J Am Gastroenterol Assoc 19, no. 12 (2021): 2587–2596. 10.1016/j.cgh.2021.01.029 PMC8661127 33493696

[315] P. Muratori , C. Lalanne , E. Barbato , et al., “Features and Progression of Asymptomatic Autoimmune Hepatitis in Italy,” Clin Gastroenterol Hepatol Off Clin Pract J Am Gastroenterol Assoc 14, no. 1 (2016): 139–146. 10.1016/j.cgh.2015.07.017 26192146

[316] L. Grønbaek , H. Vilstrup , L. Pedersen , et al., “Extrahepatic Autoimmune Diseases in Patients With Autoimmune hepatitis and Their Relatives: A Danish Nationwide Cohort Study,” Liver Int Off J Int Assoc Study Liver 39, no. 1 (2019): 205–214. 10.1111/liv.13963 30218621

[317] S. Lefere , T. Puengel , J. Hundertmark , et al., “Differential Effects of Selective‐ and Pan‐PPAR Agonists on Experimental Steatohepatitis and Hepatic Macrophages☆,” Journal of Hepatology 73, no. 4 (2020): 757–770. 32360434 10.1016/j.jhep.2020.04.025

[318] P. Muratori , A. Fabbri , C. Lalanne , et al., “Autoimmune Liver Disease and Concomitant Extrahepatic Autoimmune Disease,” European Journal of Gastroenterology & Hepatology 27, no. 10 (2015): 1175–1179. 26148248 10.1097/MEG.0000000000000424

[319] Y. Cheng , P. Zhu , J. Yang , et al., “Ischaemic Preconditioning‐regulated miR‐21 Protects Heart Against Ischaemia/Reperfusion Injury via Anti‐apoptosis Through Its Target PDCD4,” Cardiovascular Research 87, no. 3 (2010): 431–439. 20219857 10.1093/cvr/cvq082 PMC2904662

[320] Z. Li , P.‐P. Feng , Z.‐B. Zhao , et al., “Liraglutide Protects Against Inflammatory Stress in Non‐alcoholic Fatty Liver by Modulating Kupffer Cells M2 Polarization via cAMP‐PKA‐STAT3 Signaling Pathway,” Biochemical and Biophysical Research Communications 510, no. 1 (2019): 20–26. 30683312 10.1016/j.bbrc.2018.12.149

[321] F. Tacke , T. Puengel , R. Loomba , et al., “An Integrated View of Anti‐inflammatory and Antifibrotic Targets for the Treatment of NASH,” Journal of Hepatology 79, no. 2 (2023): 552–566. 37061196 10.1016/j.jhep.2023.03.038 PMC12164378

[322] M. Vuerich , R. Harshe , L. A. Frank , et al., “Altered Aryl‐hydrocarbon‐receptor Signalling Affects Regulatory and Effector Cell Immunity in Autoimmune hepatitis,” Journal of Hepatology 74, no. 1 (2021): 48–57. 32663496 10.1016/j.jhep.2020.06.044 PMC7749856

[323] M. Mendizabal , S. Marciano , M. G. Videla , et al., “Fulminant Presentation of Autoimmune hepatitis: Clinical Features and Early Predictors of Corticosteroid Treatment Failure,” European Journal of Gastroenterology & Hepatology 27, no. 6 (2015): 644–648. 25923939 10.1097/MEG.0000000000000353

[324] Y. Ni , F. Zhuge , L. Ni , et al., “CX3CL1/CX3CR1 interaction Protects Against Lipotoxicity‐induced Nonalcoholic Steatohepatitis by Regulating Macrophage Migration and M1/M2 Status,” Metabolism 136 (2022): 155272. 35914622 10.1016/j.metabol.2022.155272

[325] F. Xu , M. Guo , W. Huang , et al., “Annexin A5 Regulates Hepatic Macrophage Polarization via Directly Targeting PKM2 and Ameliorates NASH,” Redox Biology 36 (2020): 101634. 32863213 10.1016/j.redox.2020.101634 PMC7369618

[326] W. Luo , Q. Xu , Q. Wang , et al., “Effect of Modulation of PPAR‐γ Activity on Kupffer Cells M1/M2 Polarization in the Development of Non‐alcoholic Fatty Liver Disease,” Scientific Reports 7 (2017): 44612. 28300213 10.1038/srep44612 PMC5353732

[327] N. Sonthalia , P. M. Rathi , S. S. Jain , et al., “Natural History and Treatment Outcomes of Severe Autoimmune Hepatitis,” Journal of Clinical Gastroenterology 51, no. 6 (2017): 548–556. 28272079 10.1097/MCG.0000000000000805

[328] X. Qu , T. Yang , X. Wang , et al., “Macrophage RIPK3 Triggers Inflammation and Cell Death via the XBP1‐Foxo1 Axis in Liver Ischaemia‐reperfusion Injury,” JHEP Reports: Innovation in Hepatology 5, no. 11 (2023): 100879. 37841640 10.1016/j.jhepr.2023.100879 PMC10568422

[329] K. Newton , D. L. Dugger , A. Maltzman , et al., “RIPK3 deficiency or Catalytically Inactive RIPK1 Provides Greater Benefit Than MLKL deficiency in Mouse Models of Inflammation and Tissue Injury,” Cell Death and Differentiation 23, no. 9 (2016): 1565–1576. 27177019 10.1038/cdd.2016.46 PMC5072432

